# Supplementary figures and images for: Target protein identification in live cells and organisms with a non-diffusive proximity tagging system
Source: eLife. 2024 Dec 27;13:RP102667. doi: 10.7554/eLife.102667 (PMC11677243; doi:10.7554/eLife.102667)

**Figure 1—figure supplement 1 B**

**B**

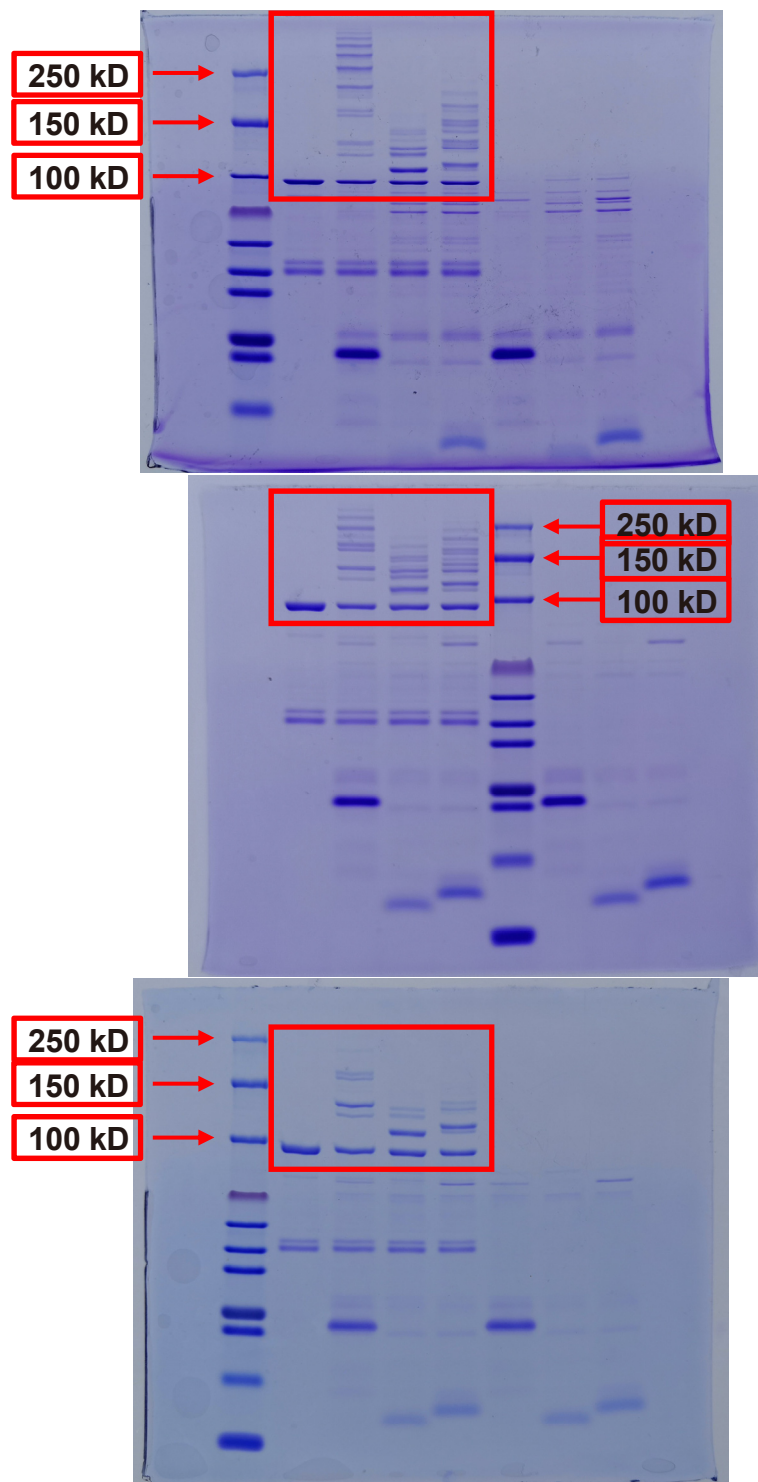

**Figure 1—figure supplement 1 C**

**C**

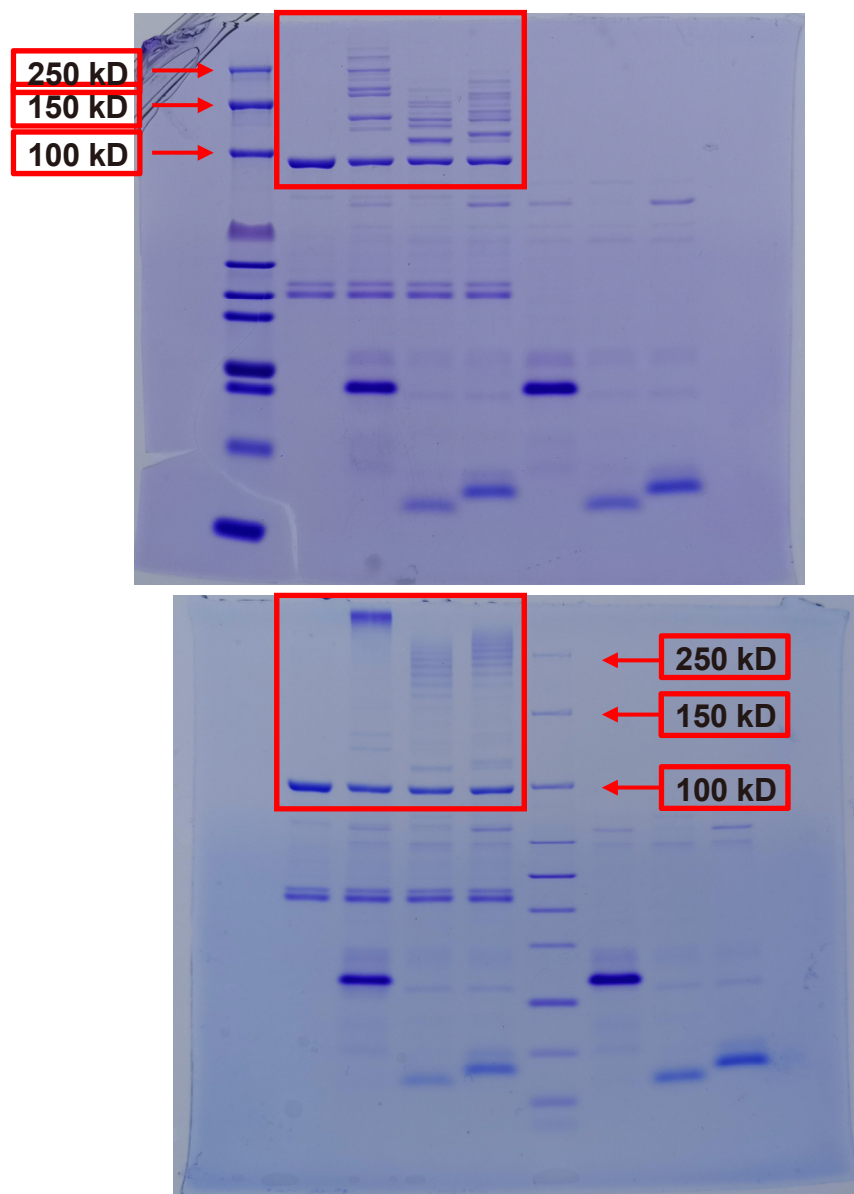

Supplement: Figure 1—figure supplement 1—source data 1. [file elife-102667-fig1-figsupp1-data1.zip › Figure 1—figure supplement 1-source data 1.pdf]

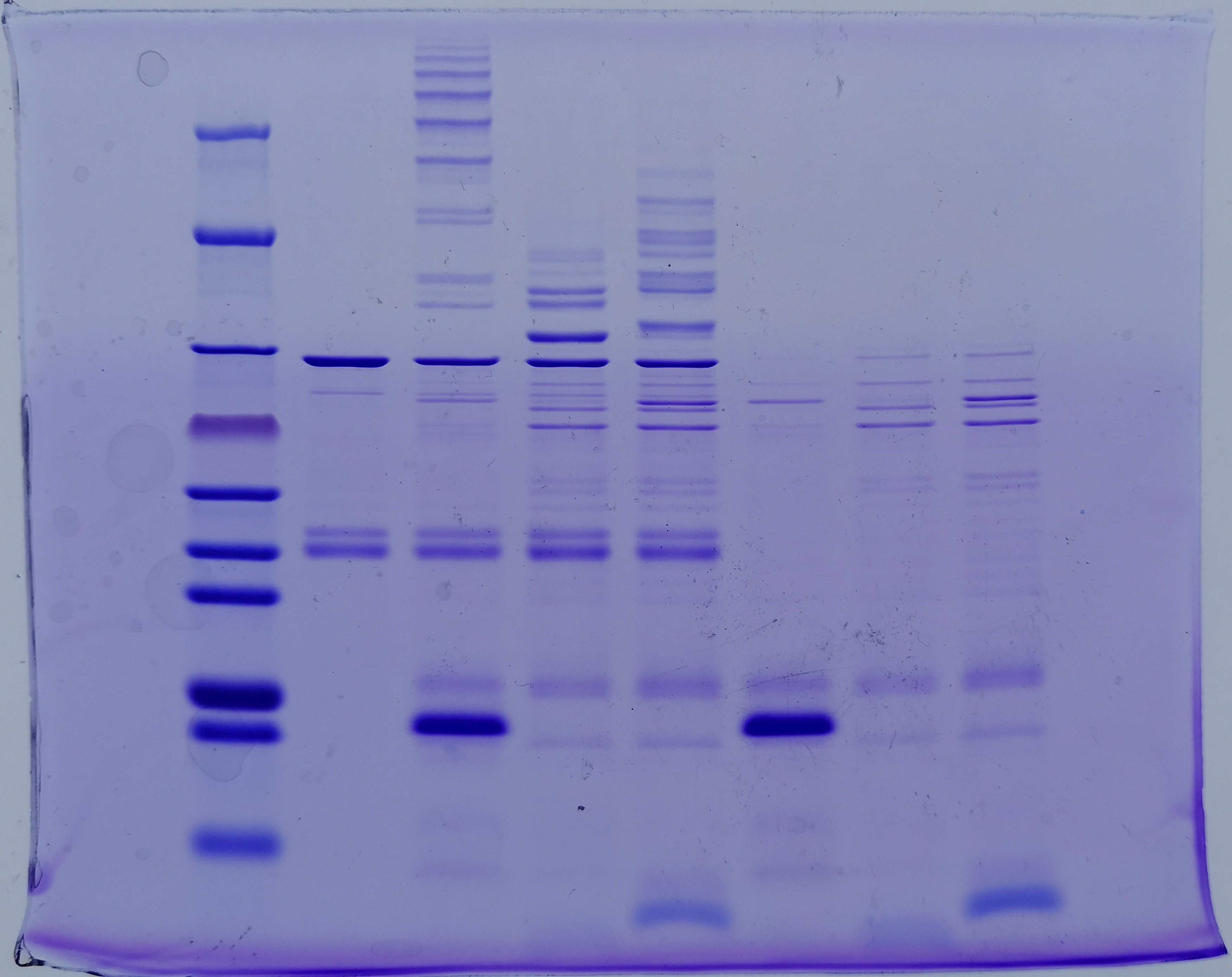

Supplement: Figure 1—figure supplement 1—source data 2. [file elife-102667-fig1-figsupp1-data2.zip › Figure 1—figure supplement 1-source data 2/Figure 1—figure supplement 1. B1.jpg]

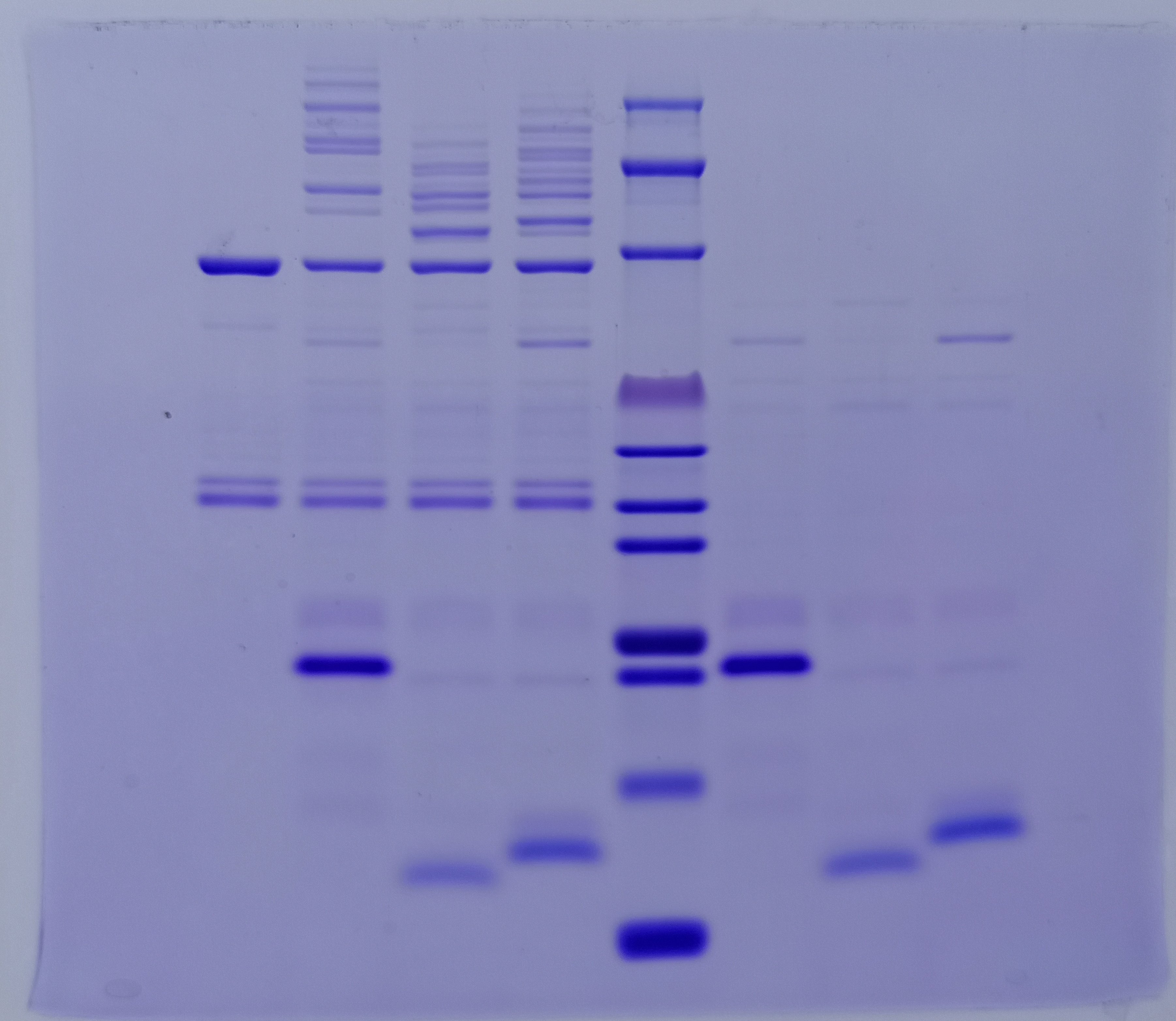

Supplement: Figure 1—figure supplement 1—source data 2. [file elife-102667-fig1-figsupp1-data2.zip › Figure 1—figure supplement 1-source data 2/Figure 1—figure supplement 1. B2.jpg]

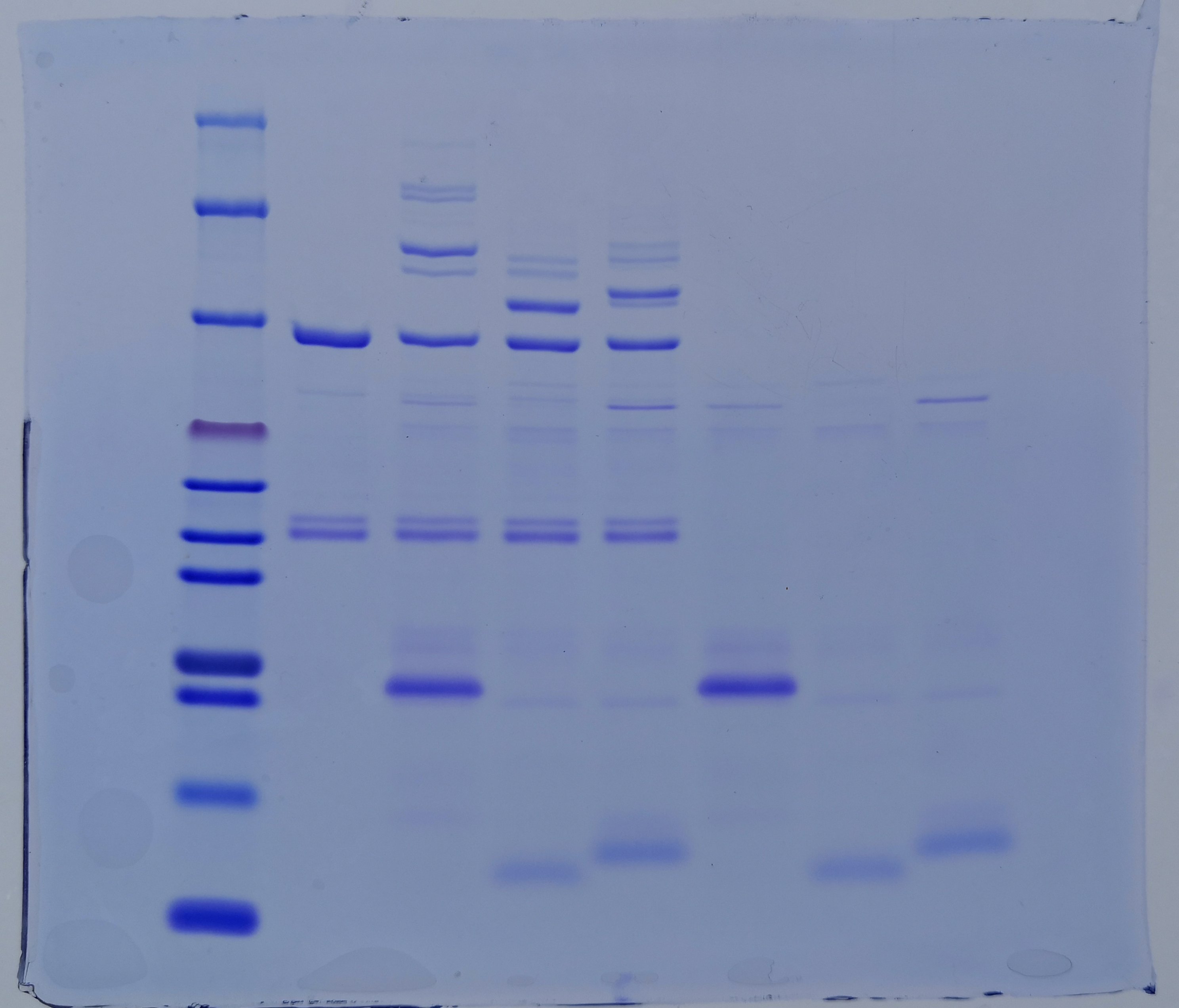

Supplement: Figure 1—figure supplement 1—source data 2. [file elife-102667-fig1-figsupp1-data2.zip › Figure 1—figure supplement 1-source data 2/Figure 1—figure supplement 1. B3.jpg]

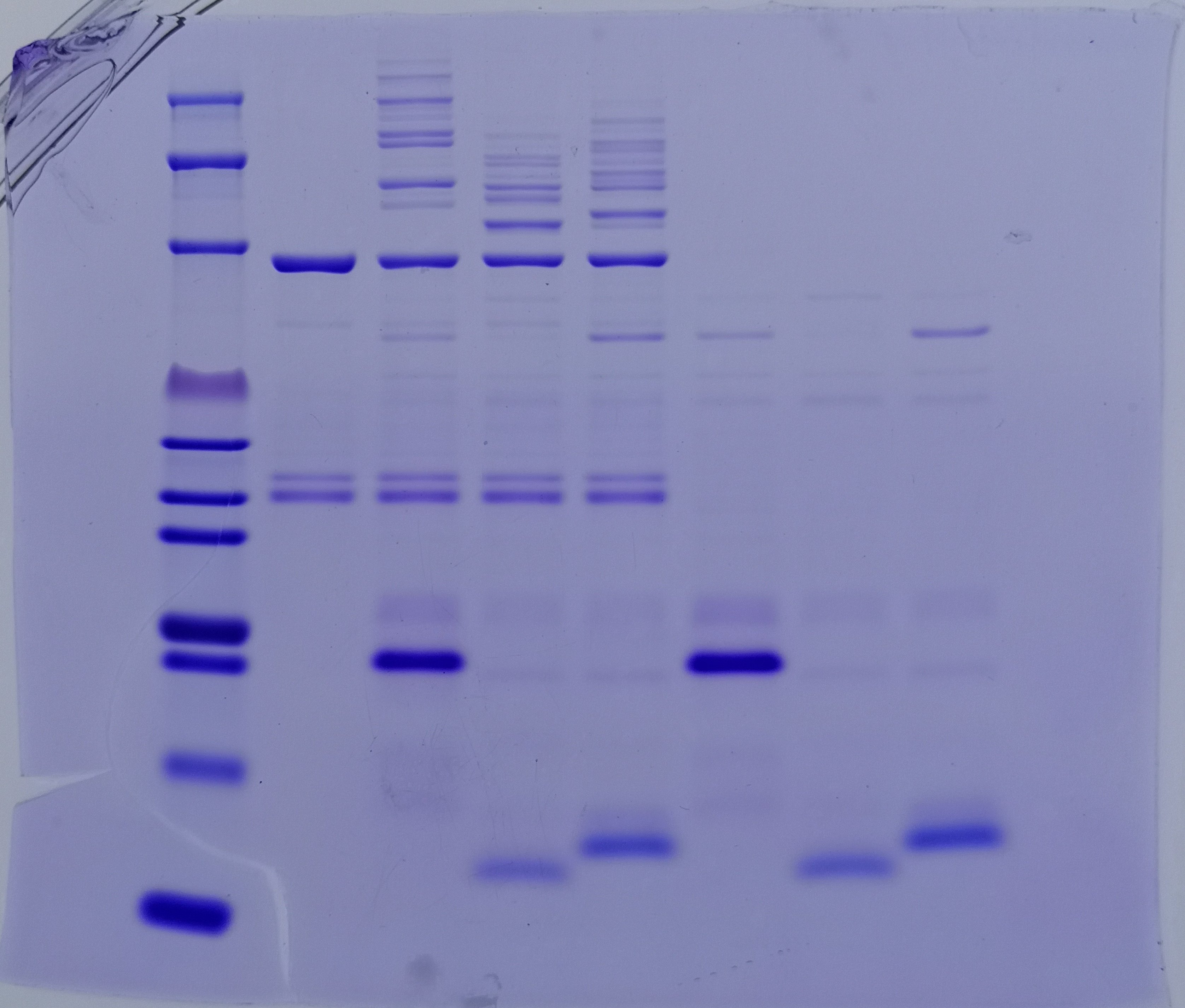

Supplement: Figure 1—figure supplement 1—source data 2. [file elife-102667-fig1-figsupp1-data2.zip › Figure 1—figure supplement 1-source data 2/Figure 1—figure supplement 1. C1.jpg]

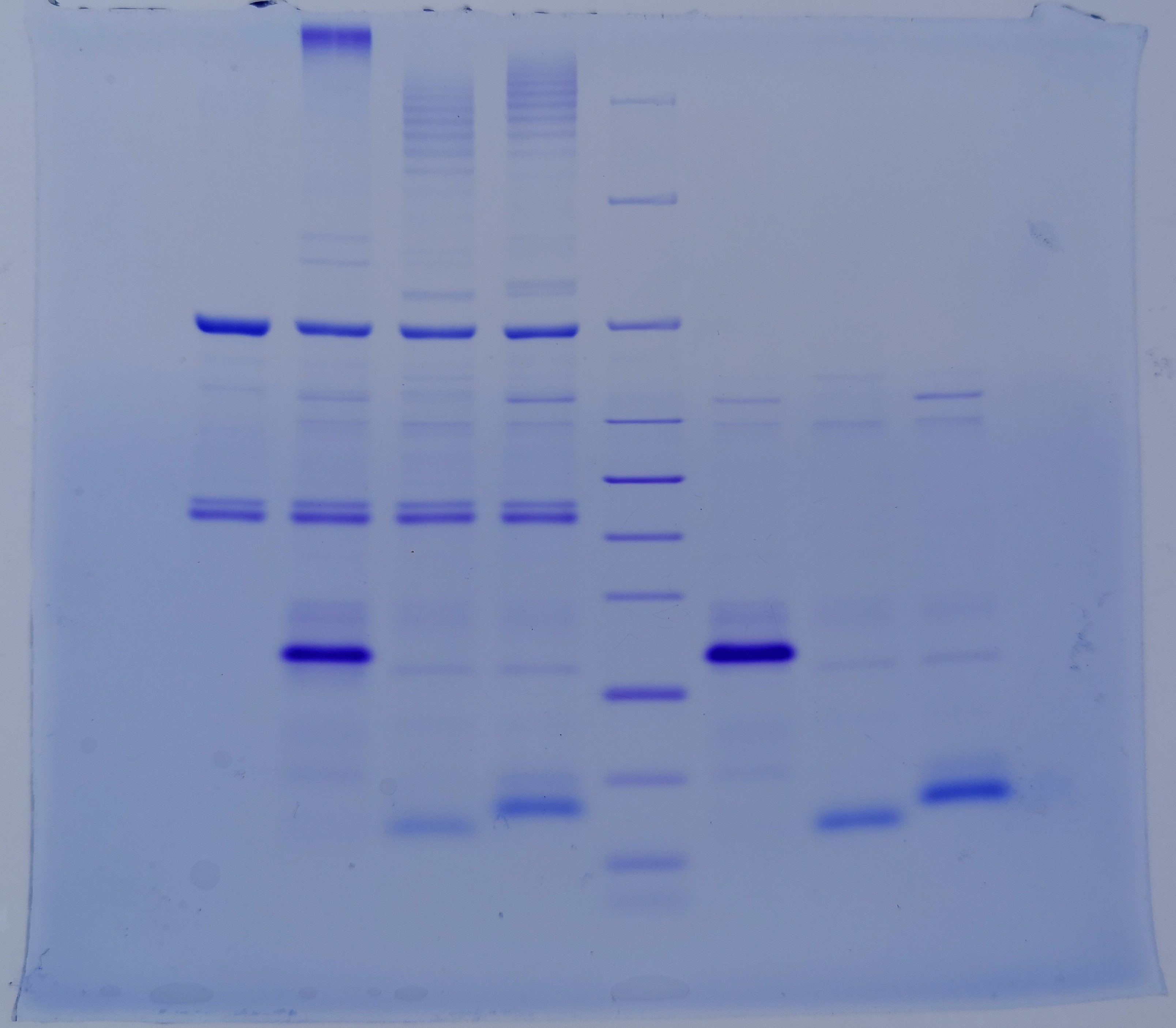

Supplement: Figure 1—figure supplement 1—source data 2. [file elife-102667-fig1-figsupp1-data2.zip › Figure 1—figure supplement 1-source data 2/Figure 1—figure supplement 1. C2.jpg]

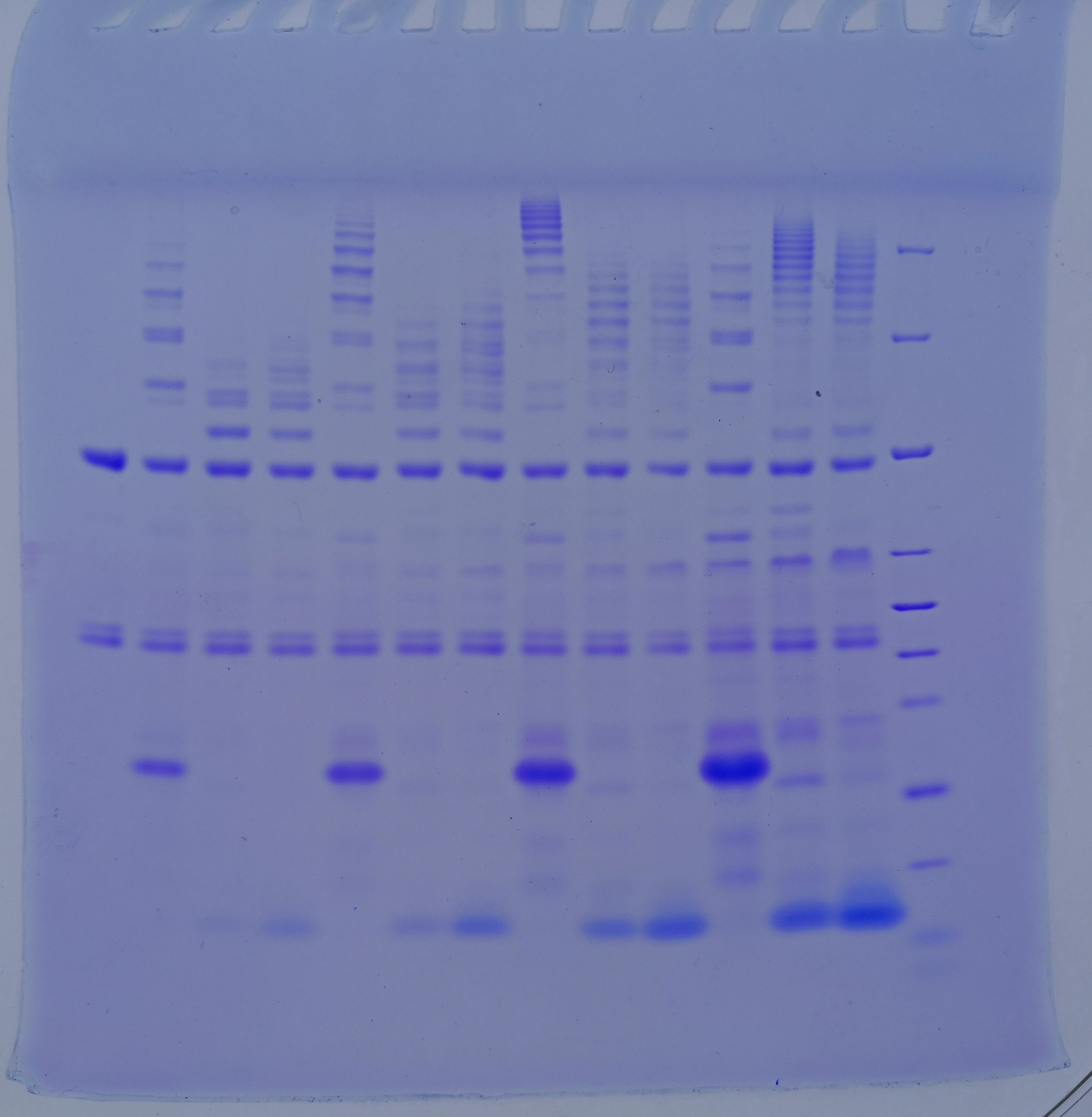

Supplement: Figure 1—figure supplement 2—source data 2. [file elife-102667-fig1-figsupp2-data2.zip › Figure 1—figure supplement 2-source data 2/Figure 1—figure supplement 2. B.jpg]

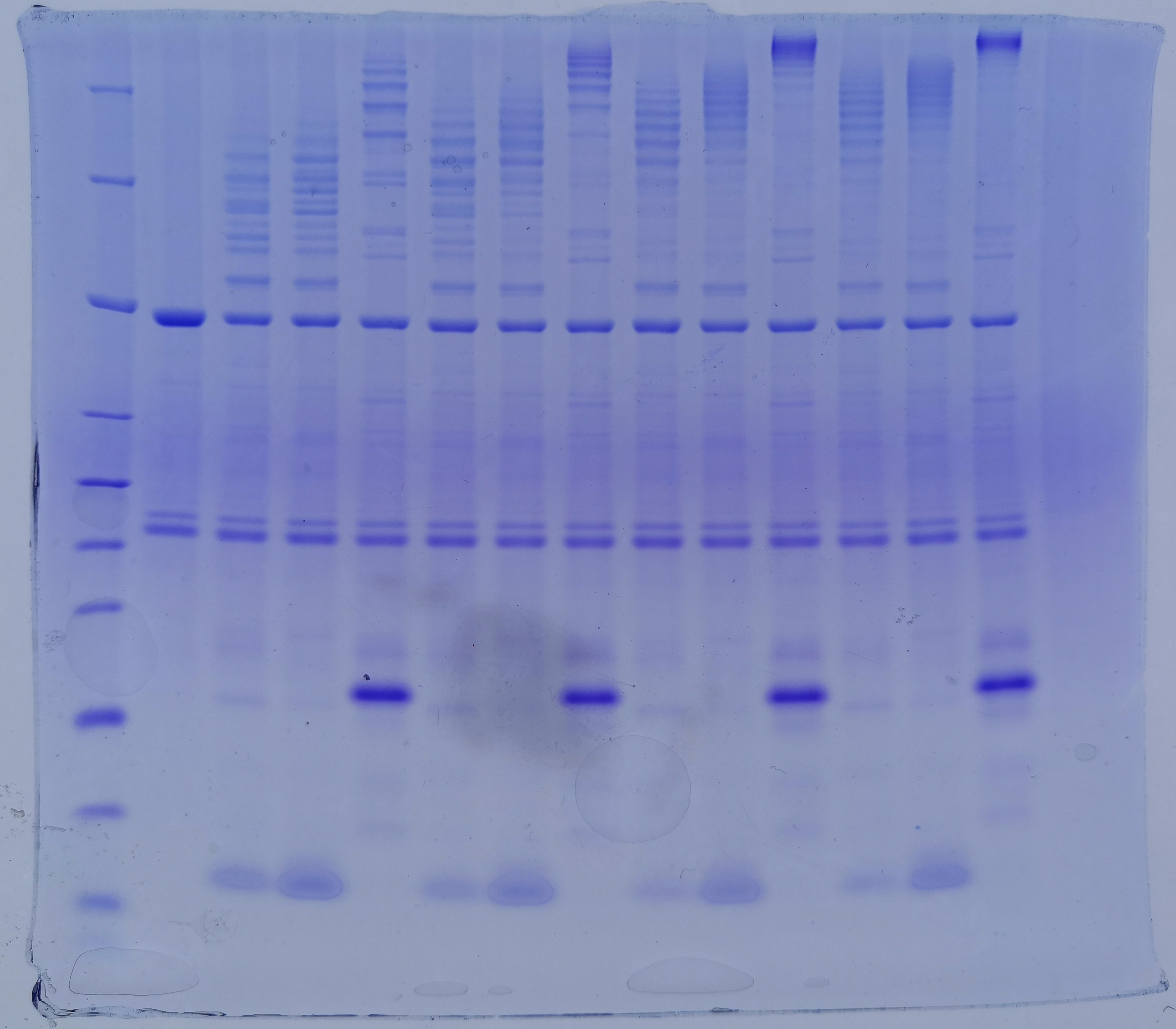

Supplement: Figure 1—figure supplement 2—source data 2. [file elife-102667-fig1-figsupp2-data2.zip › Figure 1—figure supplement 2-source data 2/Figure 1—figure supplement 2. C.jpg]

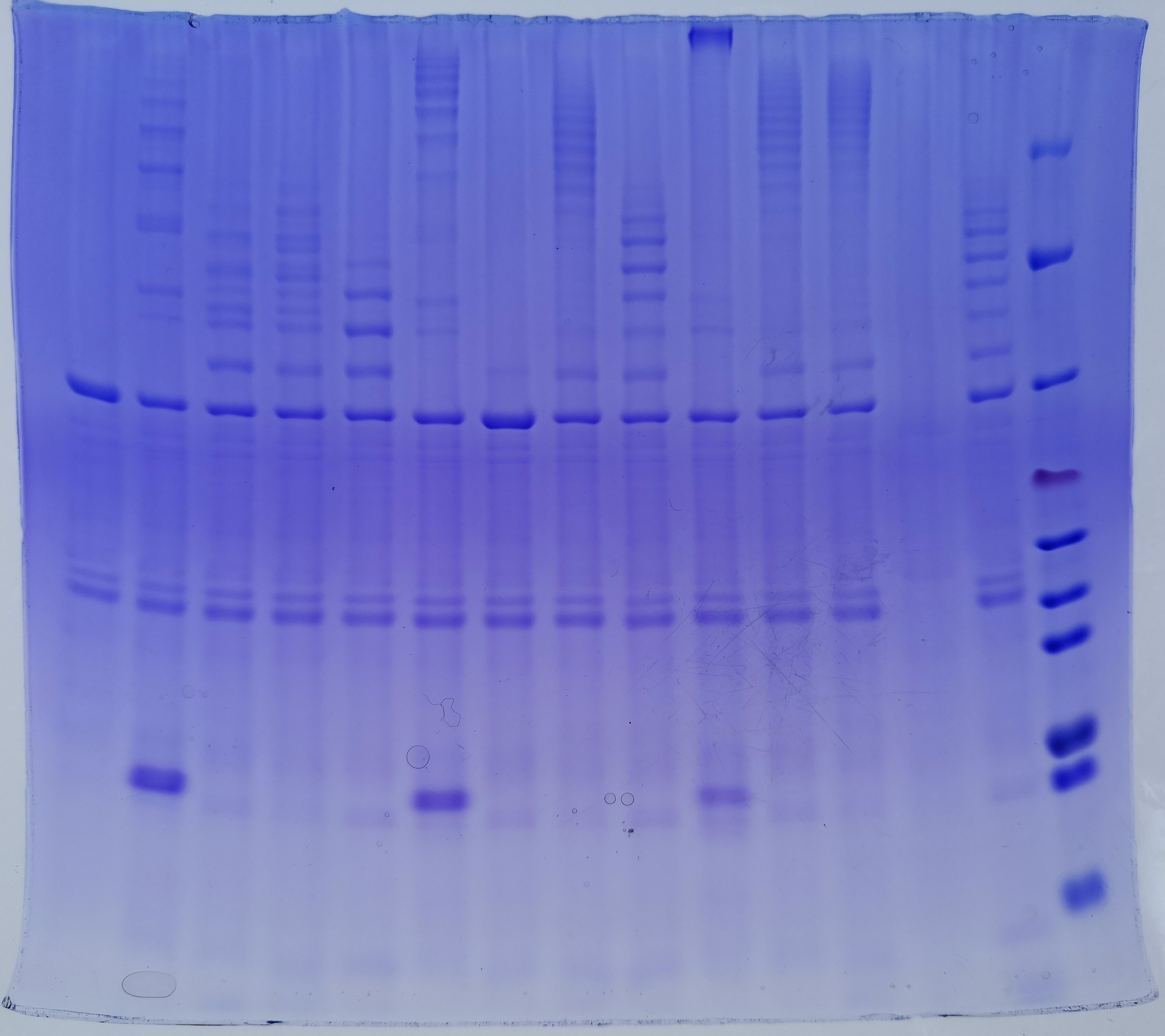

Supplement: Figure 1—figure supplement 2—source data 2. [file elife-102667-fig1-figsupp2-data2.zip › Figure 1—figure supplement 2-source data 2/Figure 1—figure supplement 2. D.jpg]

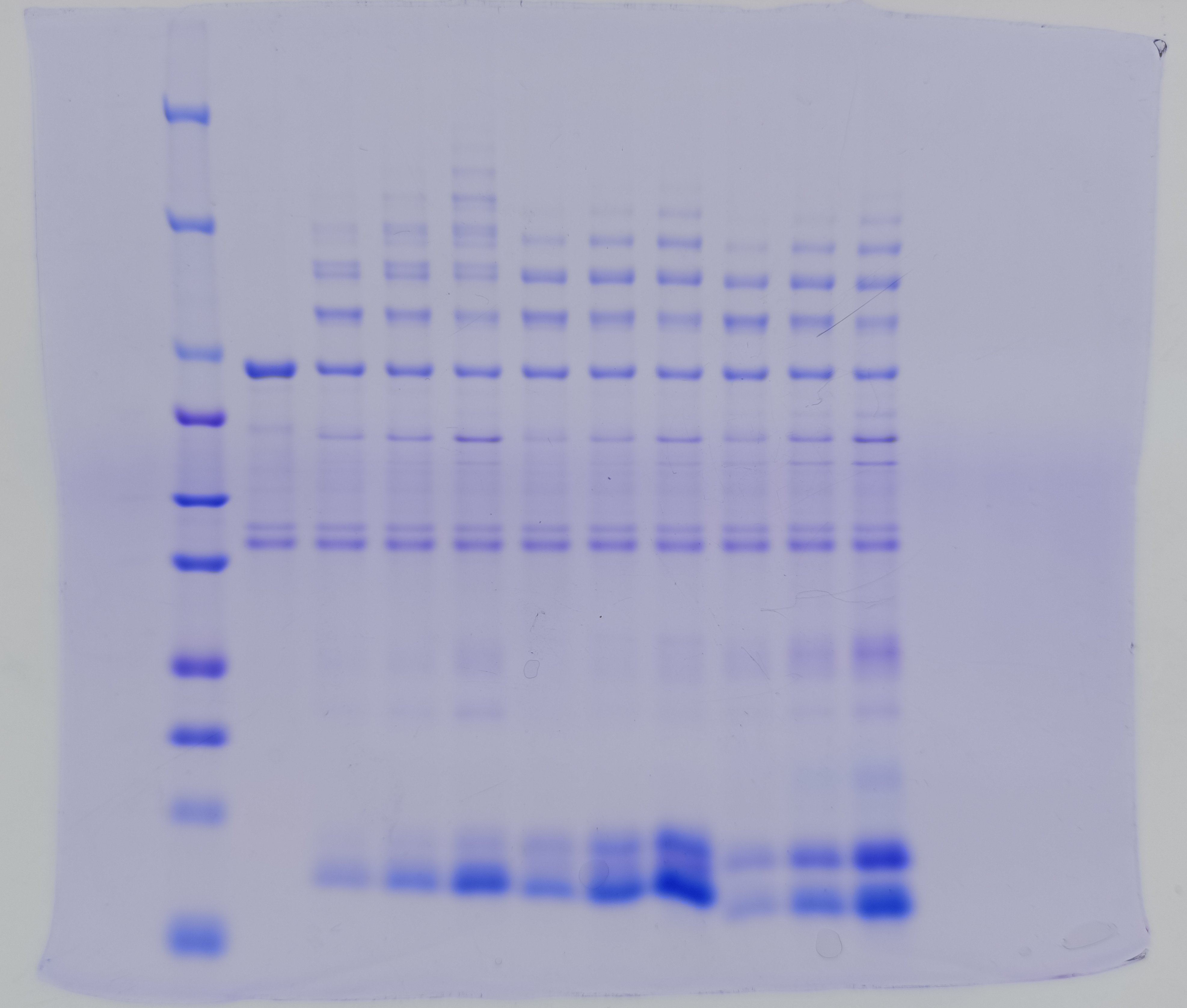

Supplement: Figure 1—figure supplement 2—source data 2. [file elife-102667-fig1-figsupp2-data2.zip › Figure 1—figure supplement 2-source data 2/Figure 1—figure supplement 2. E.jpg]

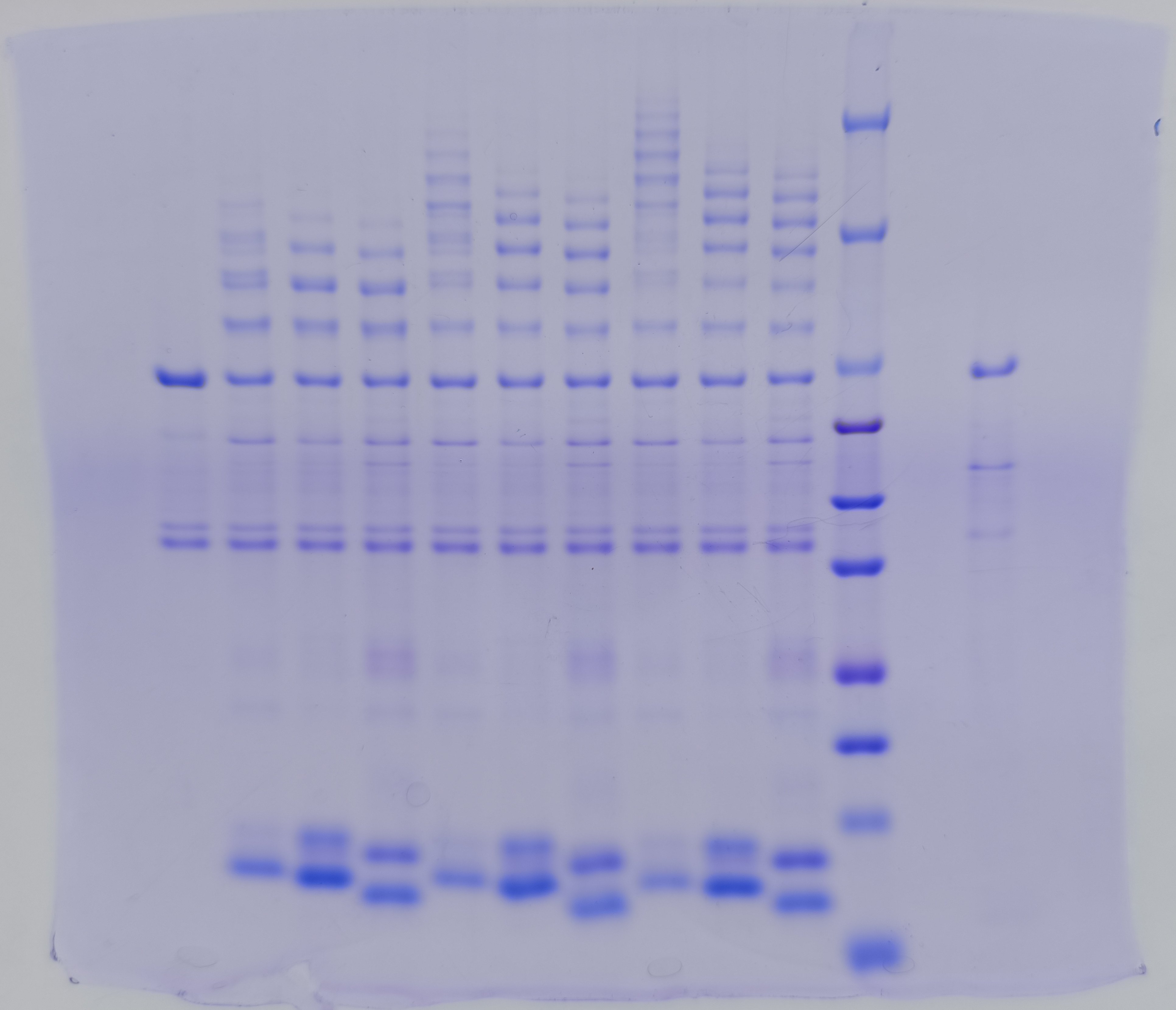

Supplement: Figure 1—figure supplement 2—source data 2. [file elife-102667-fig1-figsupp2-data2.zip › Figure 1—figure supplement 2-source data 2/Figure 1—figure supplement 2. F.jpg]

**Figure 1—figure supplement 3**

**B**

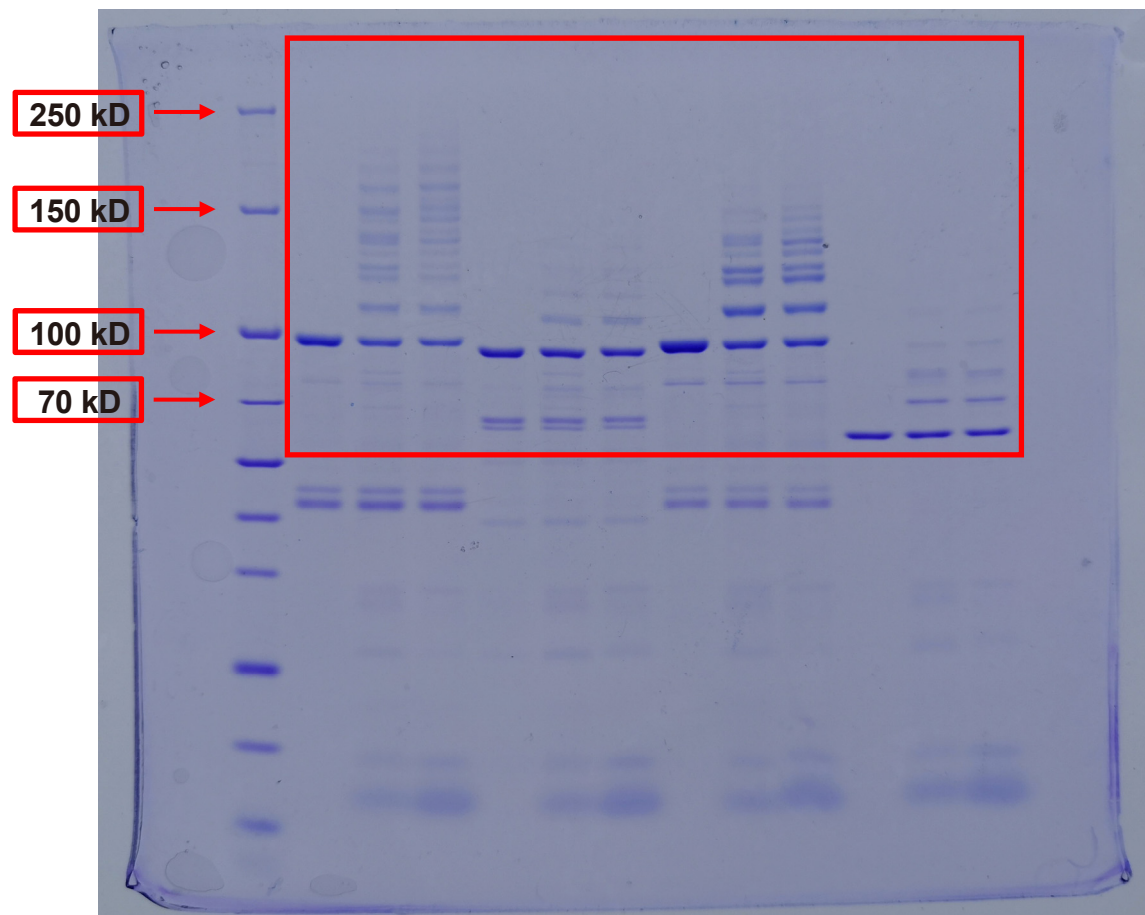

Supplement: Figure 1—figure supplement 3—source data 1. [file elife-102667-fig1-figsupp3-data1.zip › Figure 1—figure supplement 3-source data 1.pdf]

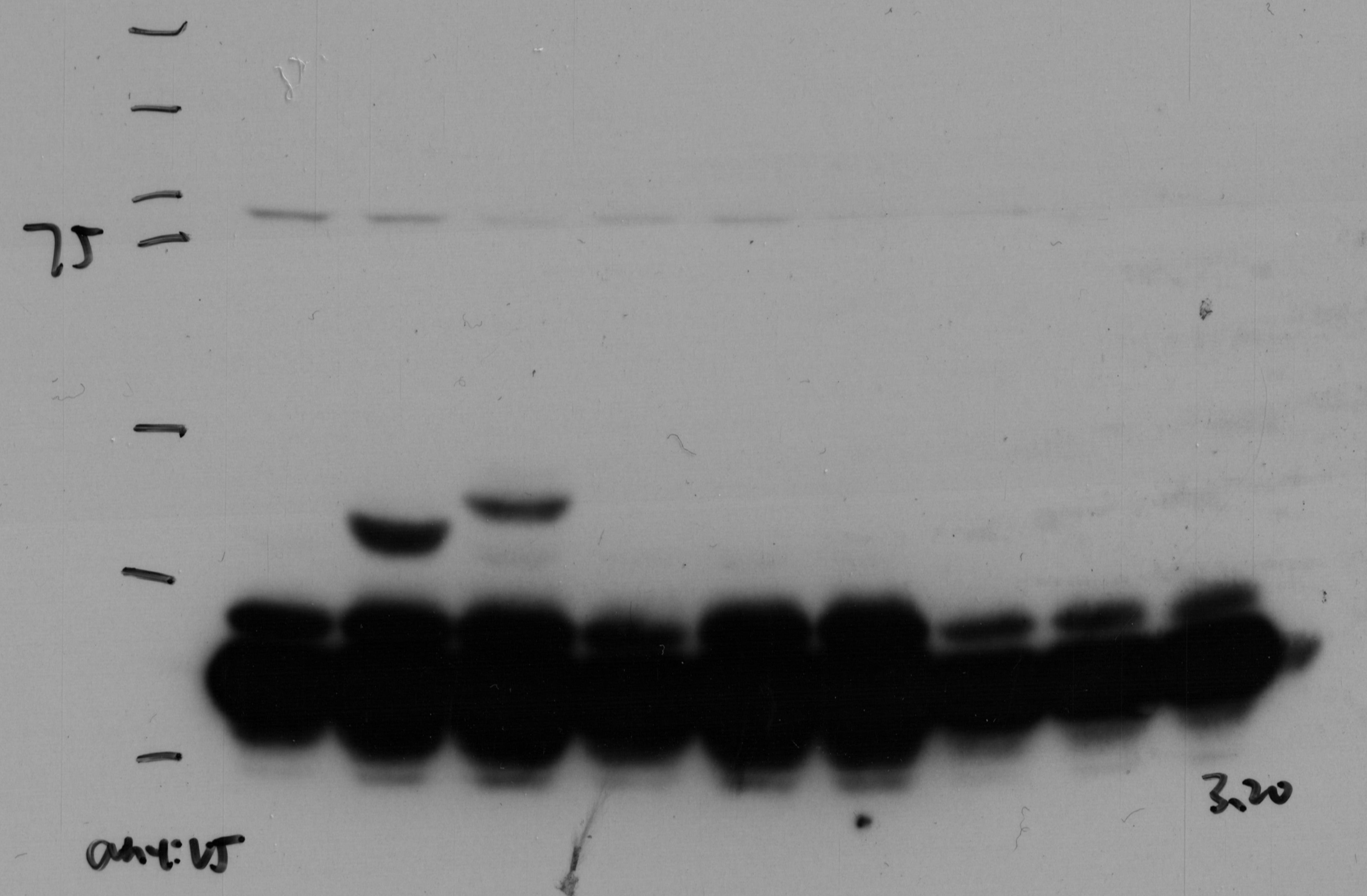

Supplement: Figure 1—figure supplement 3—source data 2. [file elife-102667-fig1-figsupp3-data2.zip › Figure 3—figure supplement 3-source data 2/Figure 3—figure supplement 3. 1.tif]

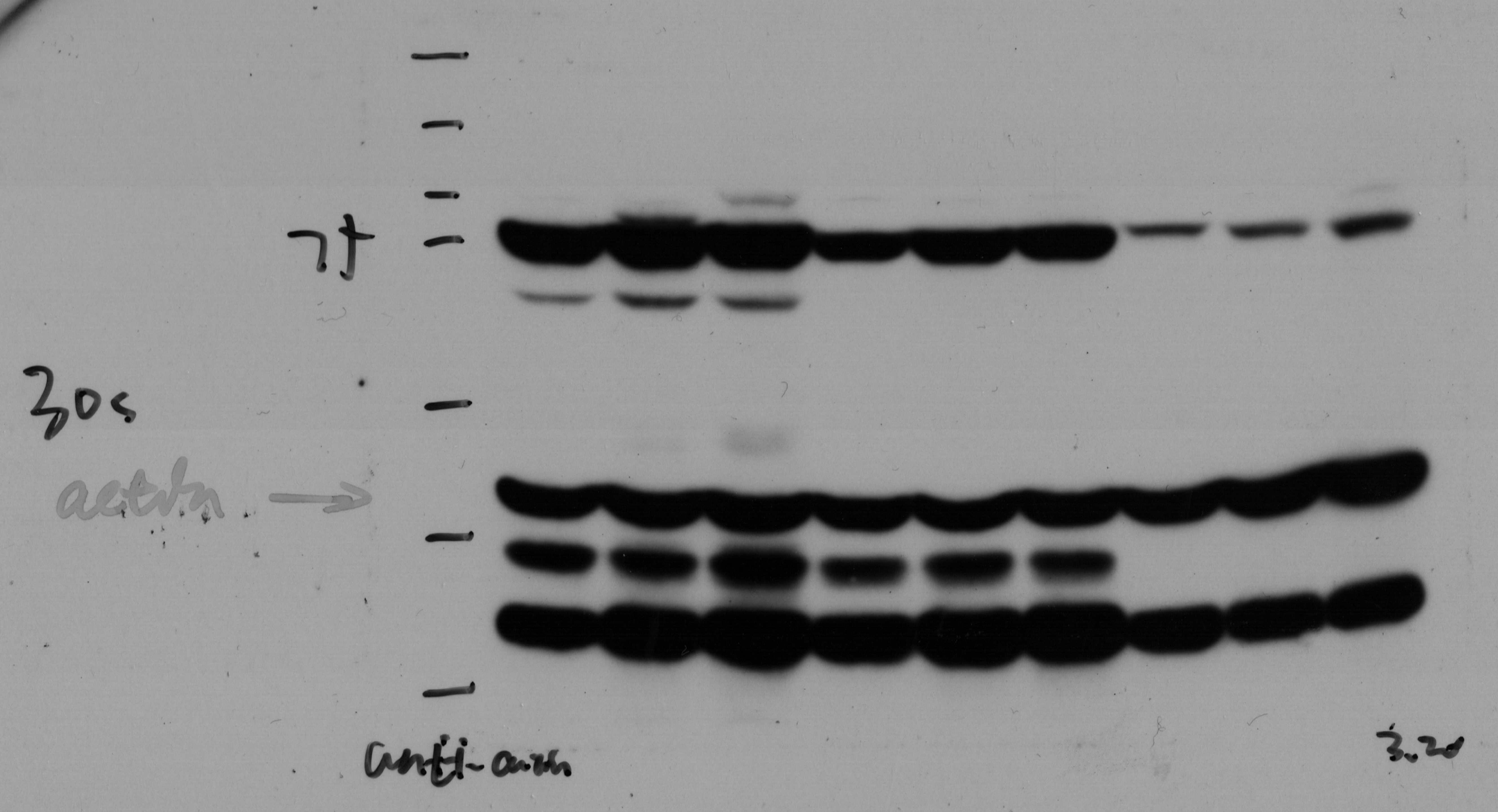

Supplement: Figure 1—figure supplement 3—source data 2. [file elife-102667-fig1-figsupp3-data2.zip › Figure 3—figure supplement 3-source data 2/Figure 3—figure supplement 3. 2.tif]

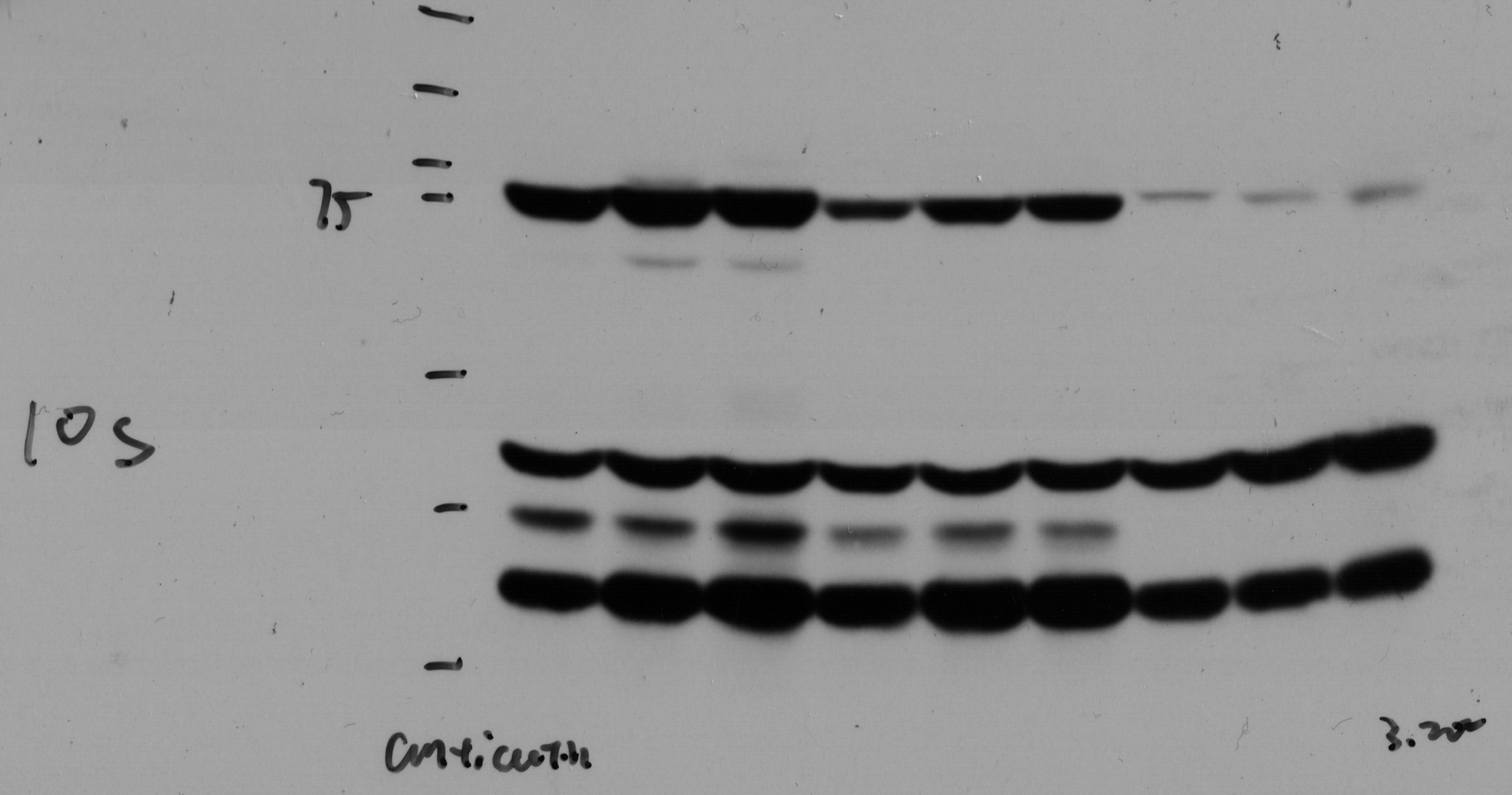

Supplement: Figure 1—figure supplement 3—source data 2. [file elife-102667-fig1-figsupp3-data2.zip › Figure 3—figure supplement 3-source data 2/Figure 3—figure supplement 3. 3.tif]

**Figure 2**

**B**

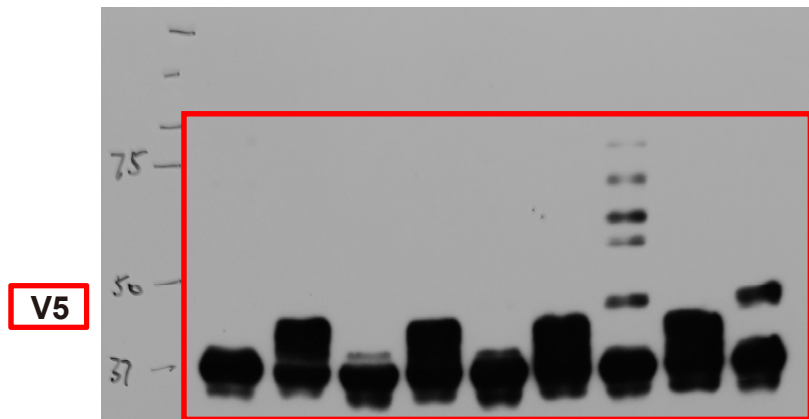

**C**

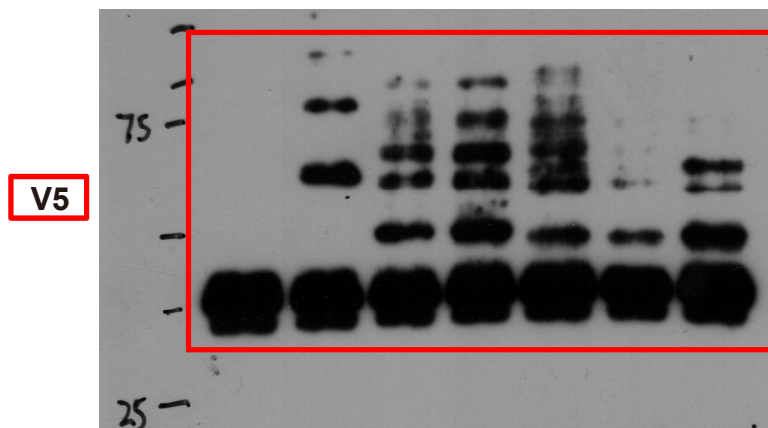

**D**

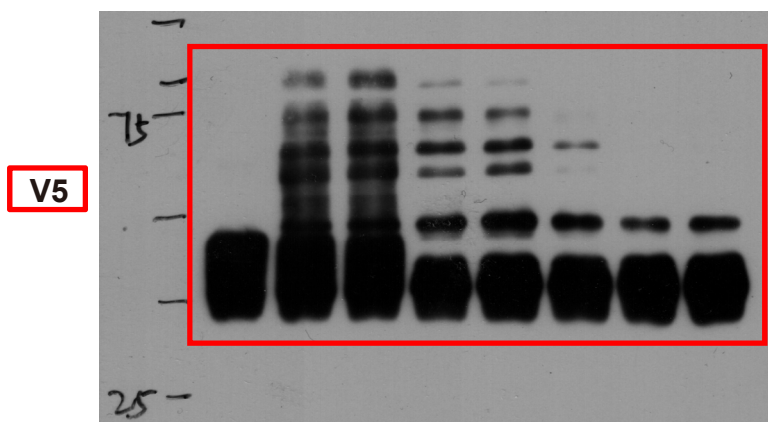

Supplement: Figure 2—source data 1. [file elife-102667-fig2-data1.zip › Figure 2-source data 1.pdf]

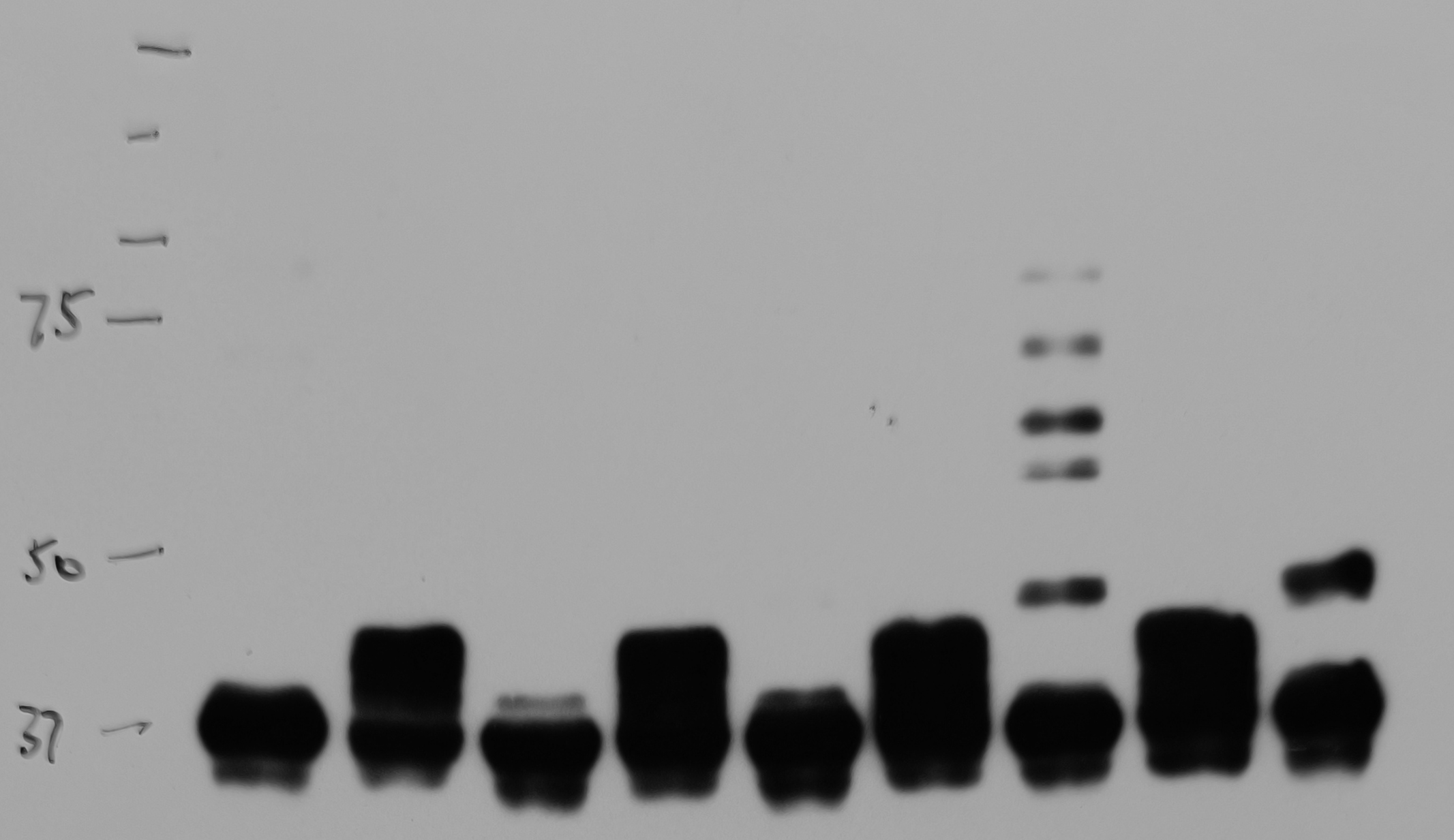

Supplement: Figure 2—source data 2. [file elife-102667-fig2-data2.zip › Figure 2-source data 2/Fig. 2. B.tif]

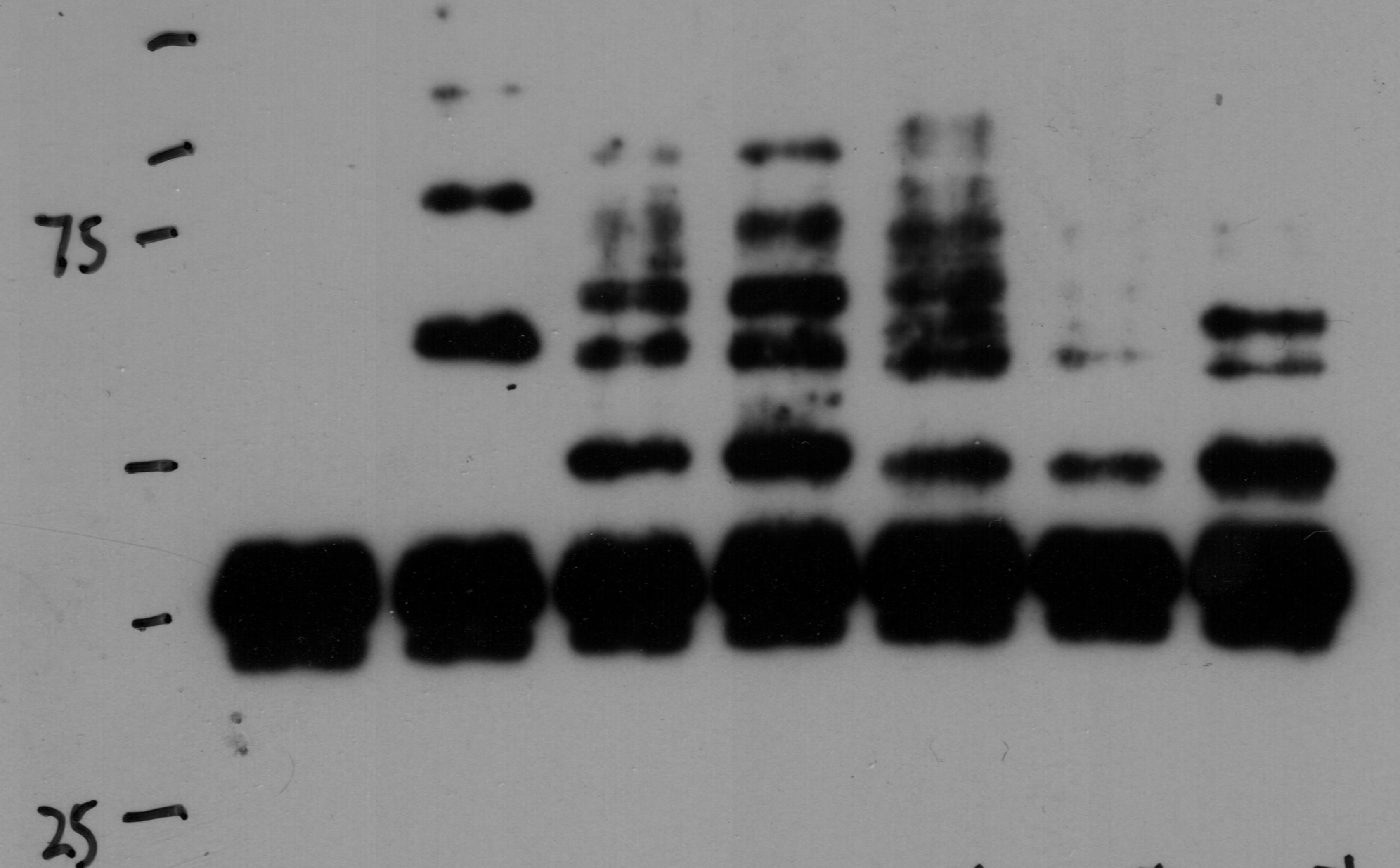

Supplement: Figure 2—source data 2. [file elife-102667-fig2-data2.zip › Figure 2-source data 2/Fig. 2. C.tif]

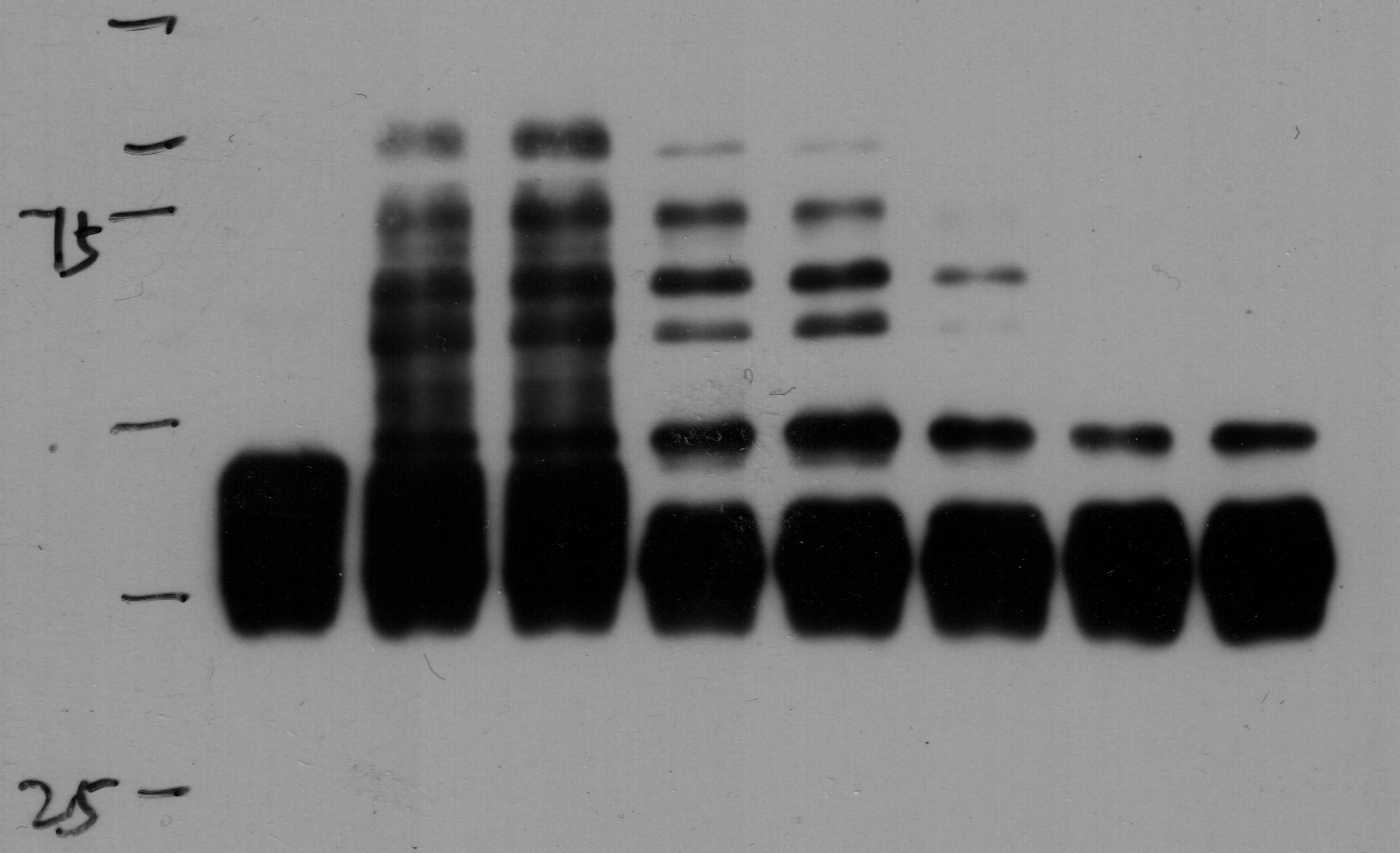

Supplement: Figure 2—source data 2. [file elife-102667-fig2-data2.zip › Figure 2-source data 2/Fig. 2. D.tif]

**Figure 2—figure supplement 3**

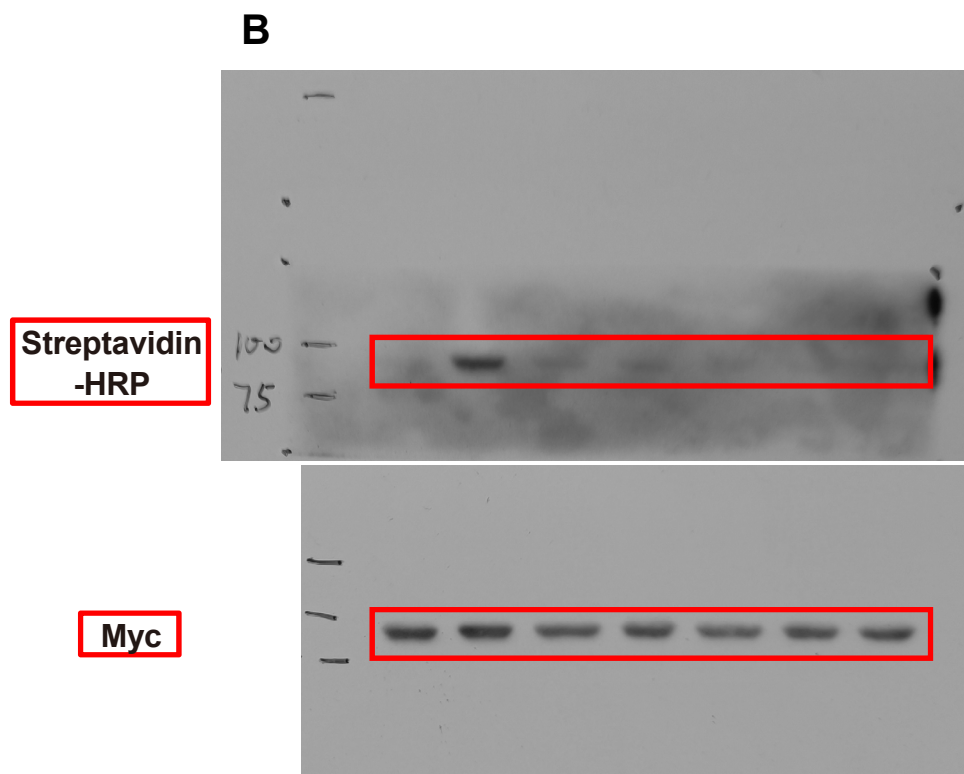

Supplement: Figure 2—figure supplement 3—source data 1. [file elife-102667-fig2-figsupp3-data1.zip › Figure 2—figure supplement 3-source data 1.pdf]

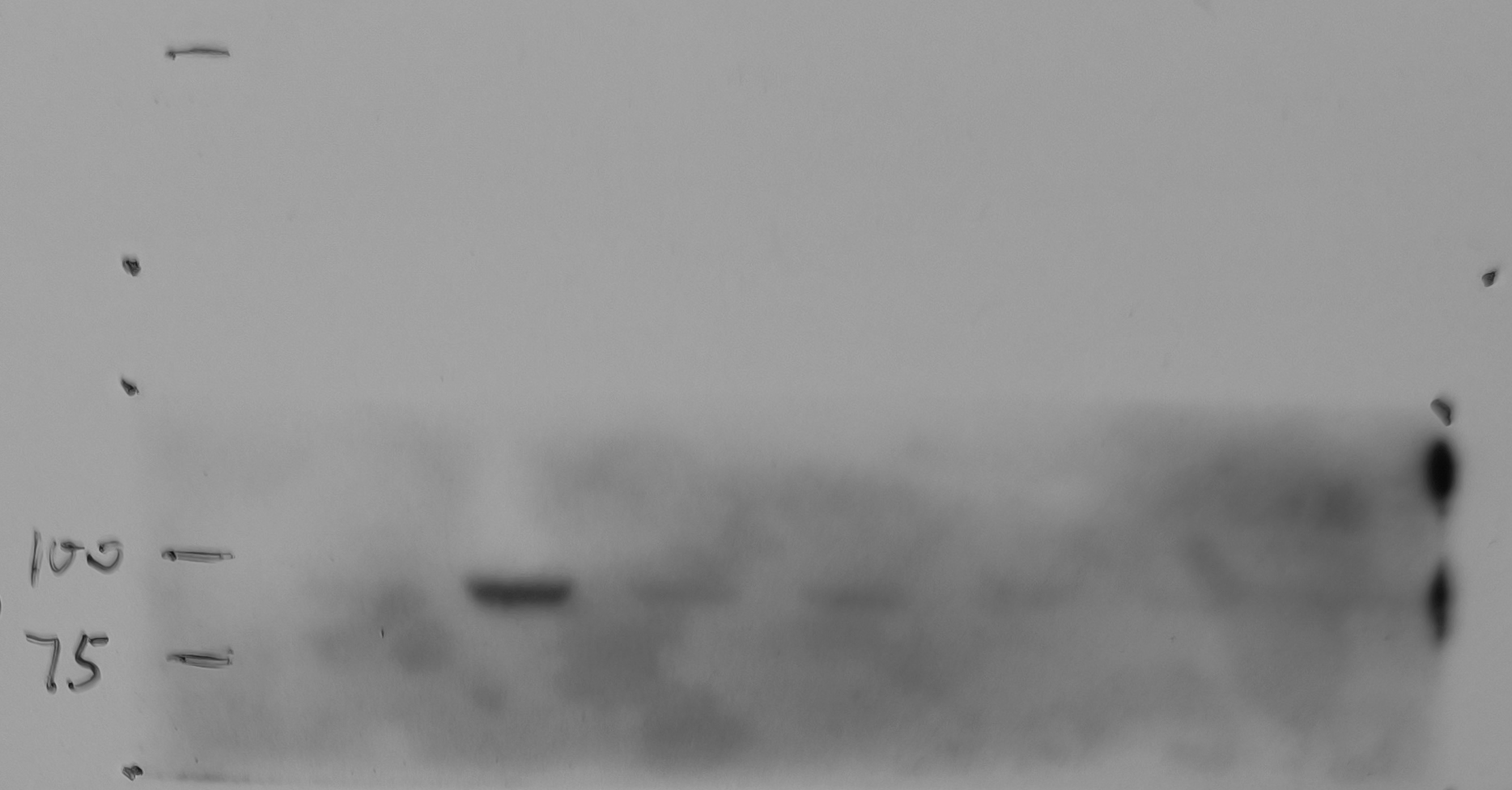

Supplement: Figure 2—figure supplement 3—source data 2. [file elife-102667-fig2-figsupp3-data2.zip › Figure 2—figure supplement 3-source data 2/Figure 2—figure supplement 3. B1.tif]

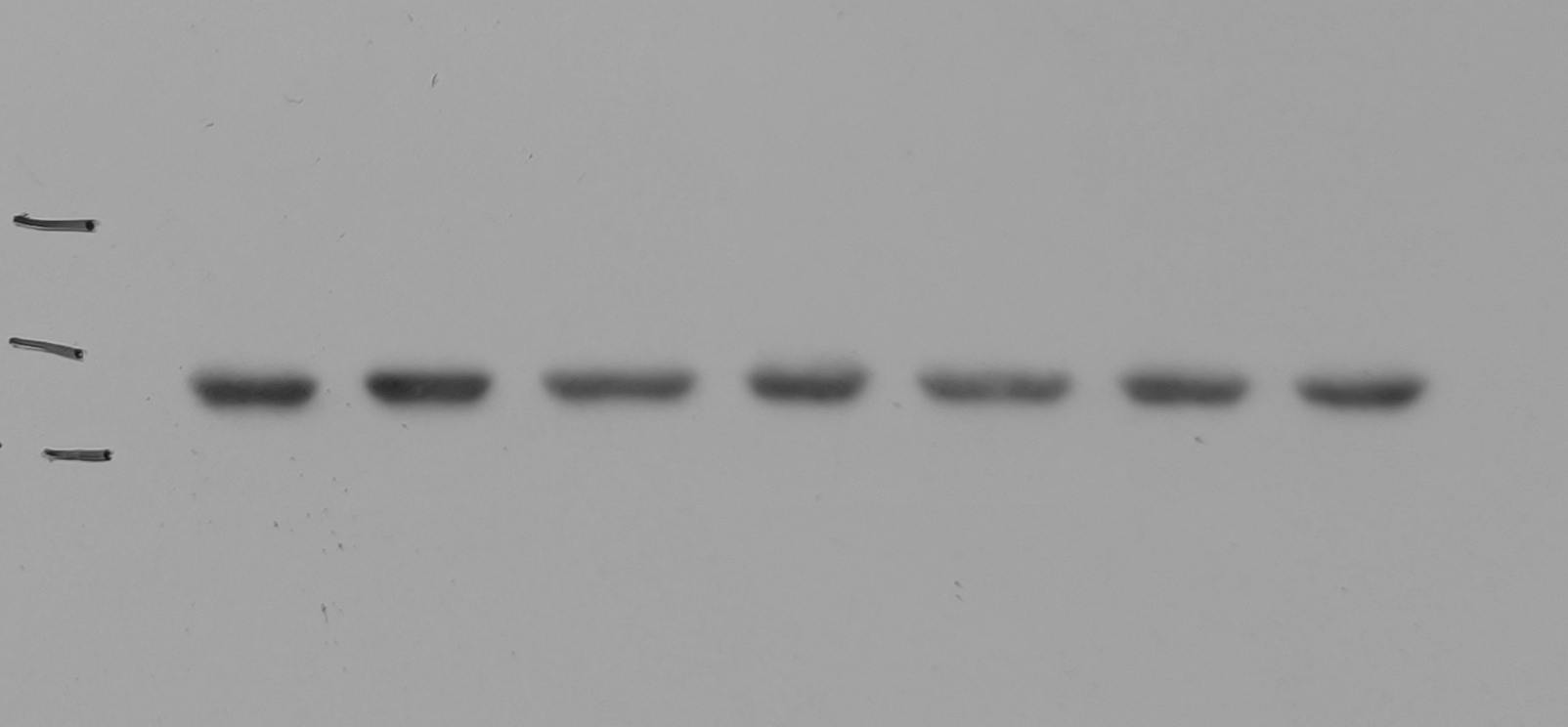

Supplement: Figure 2—figure supplement 3—source data 2. [file elife-102667-fig2-figsupp3-data2.zip › Figure 2—figure supplement 3-source data 2/Figure 2—figure supplement 3. B2.tif]

**Figure 2—figure supplement 4**

**A**

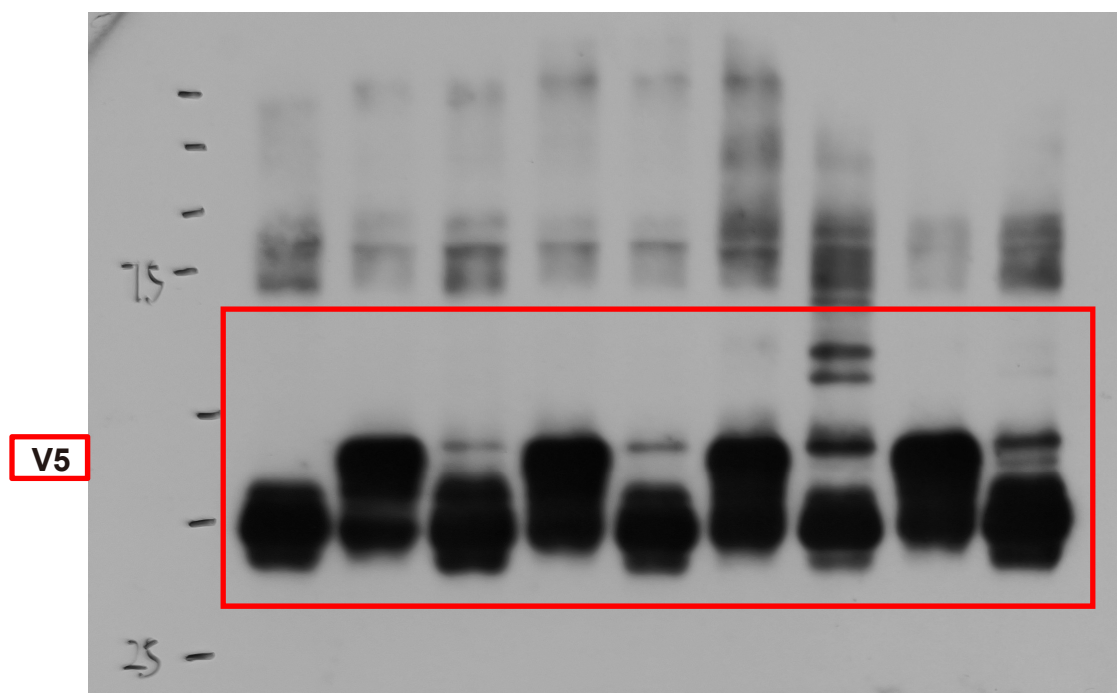

**B**

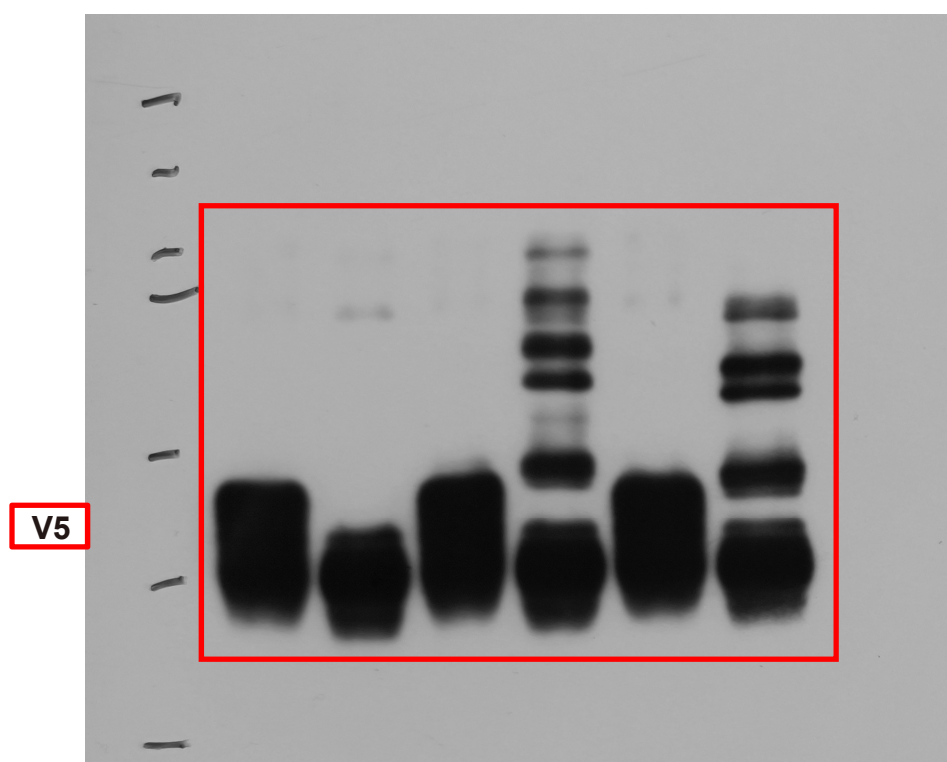

Supplement: Figure 2—figure supplement 4—source data 1. [file elife-102667-fig2-figsupp4-data1.zip › Figure 2—figure supplement 4-source data 1.pdf]

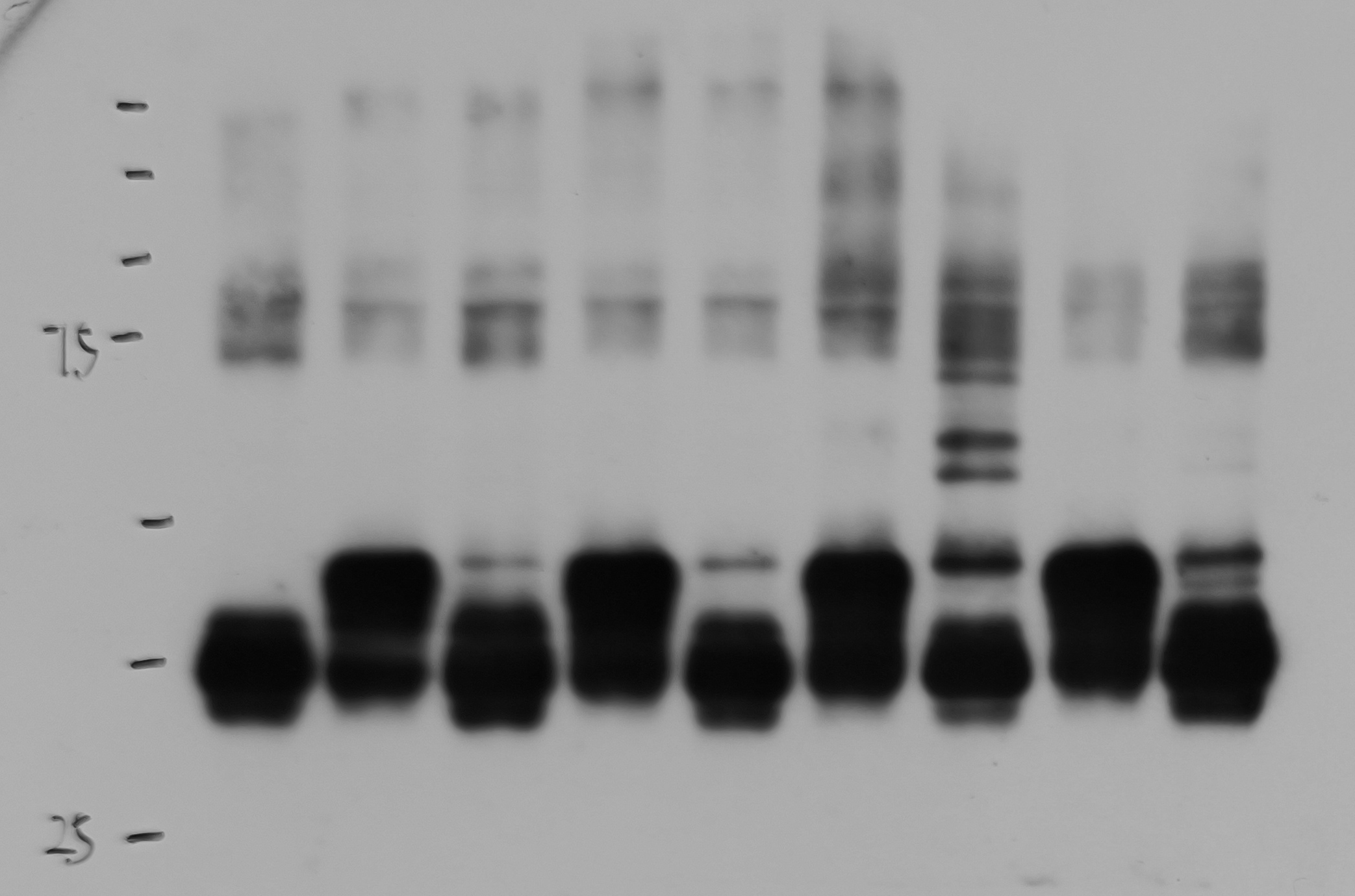

Supplement: Figure 2—figure supplement 4—source data 2. [file elife-102667-fig2-figsupp4-data2.zip › Figure 2—figure supplement 2-source data 2/Figure 2—figure supplement 2. A.tif]

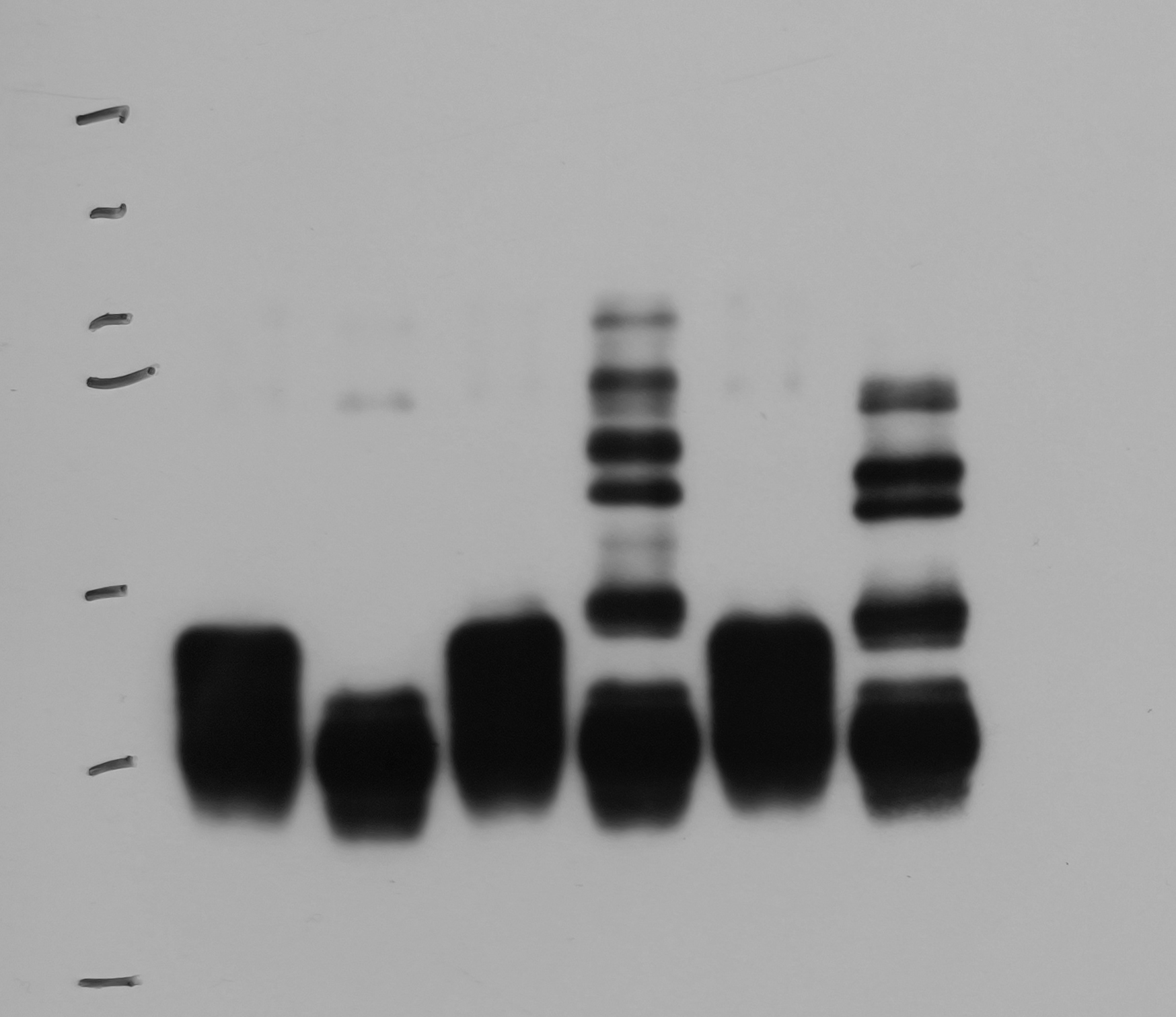

Supplement: Figure 2—figure supplement 4—source data 2. [file elife-102667-fig2-figsupp4-data2.zip › Figure 2—figure supplement 2-source data 2/Figure 2—figure supplement 2. B.tif]

**Figure 3 A-D**

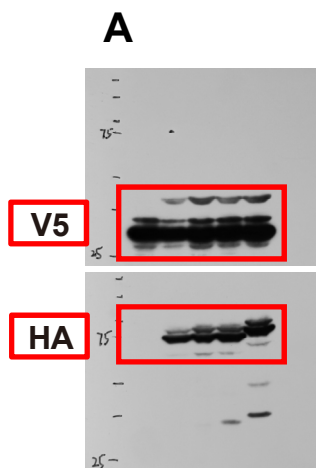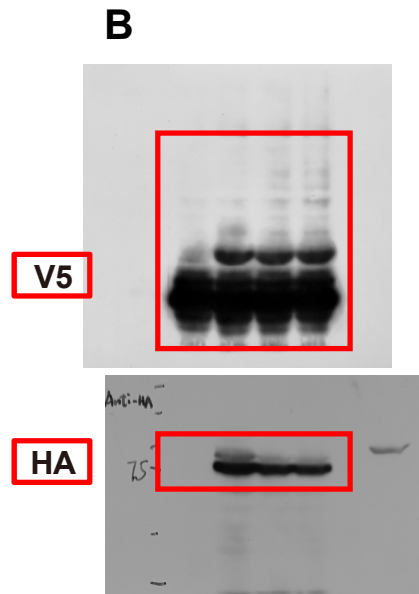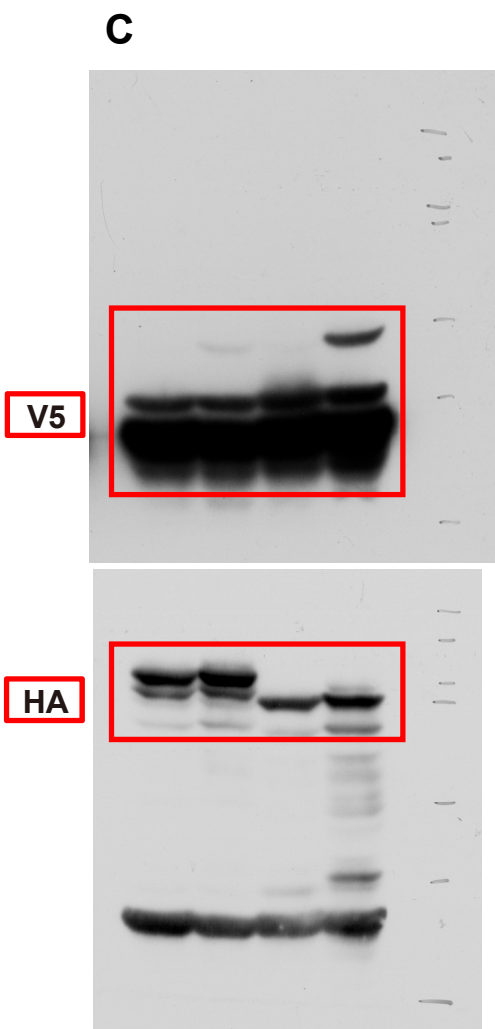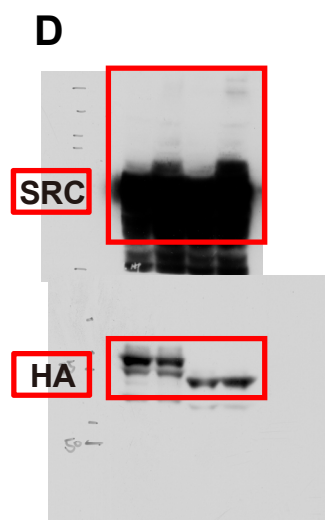

**Figure 3 E-G**

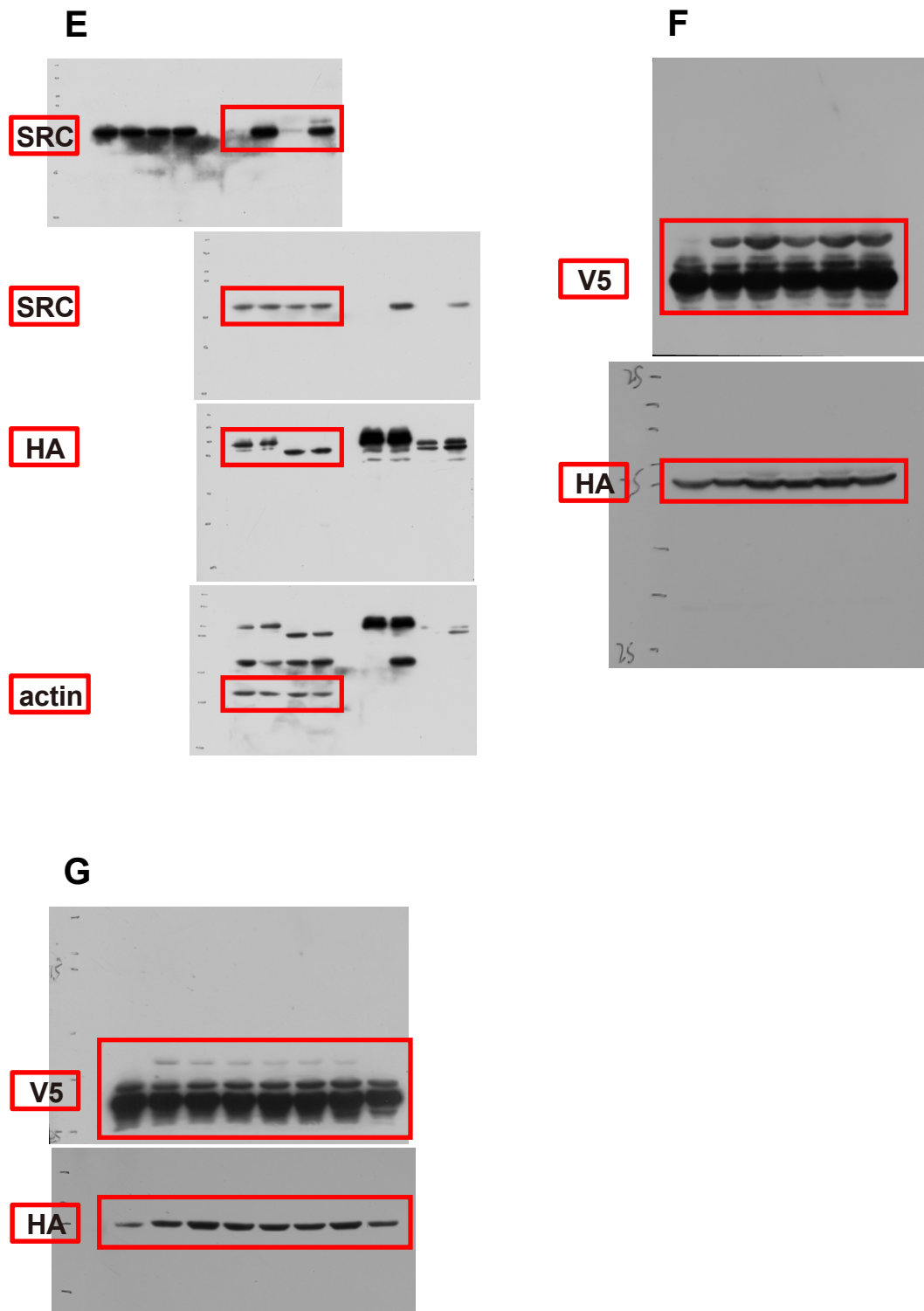

Supplement: Figure 3—source data 1. [file elife-102667-fig3-data1.zip › Figure 3-source data 1.pdf]

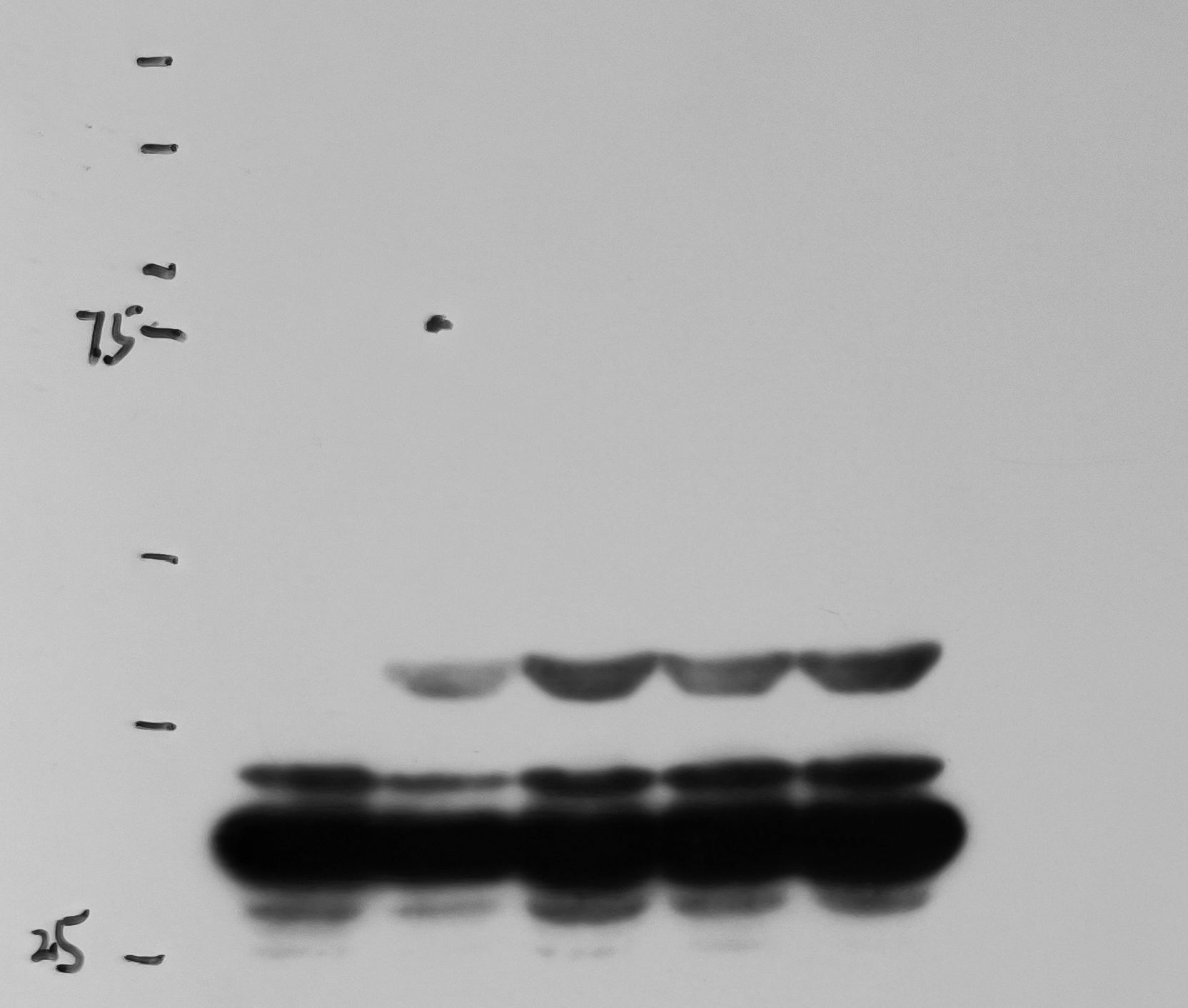

Supplement: Figure 3—source data 2. [file elife-102667-fig3-data2.zip › Figure 3-source data 2/Fig. 3. A1.tif]

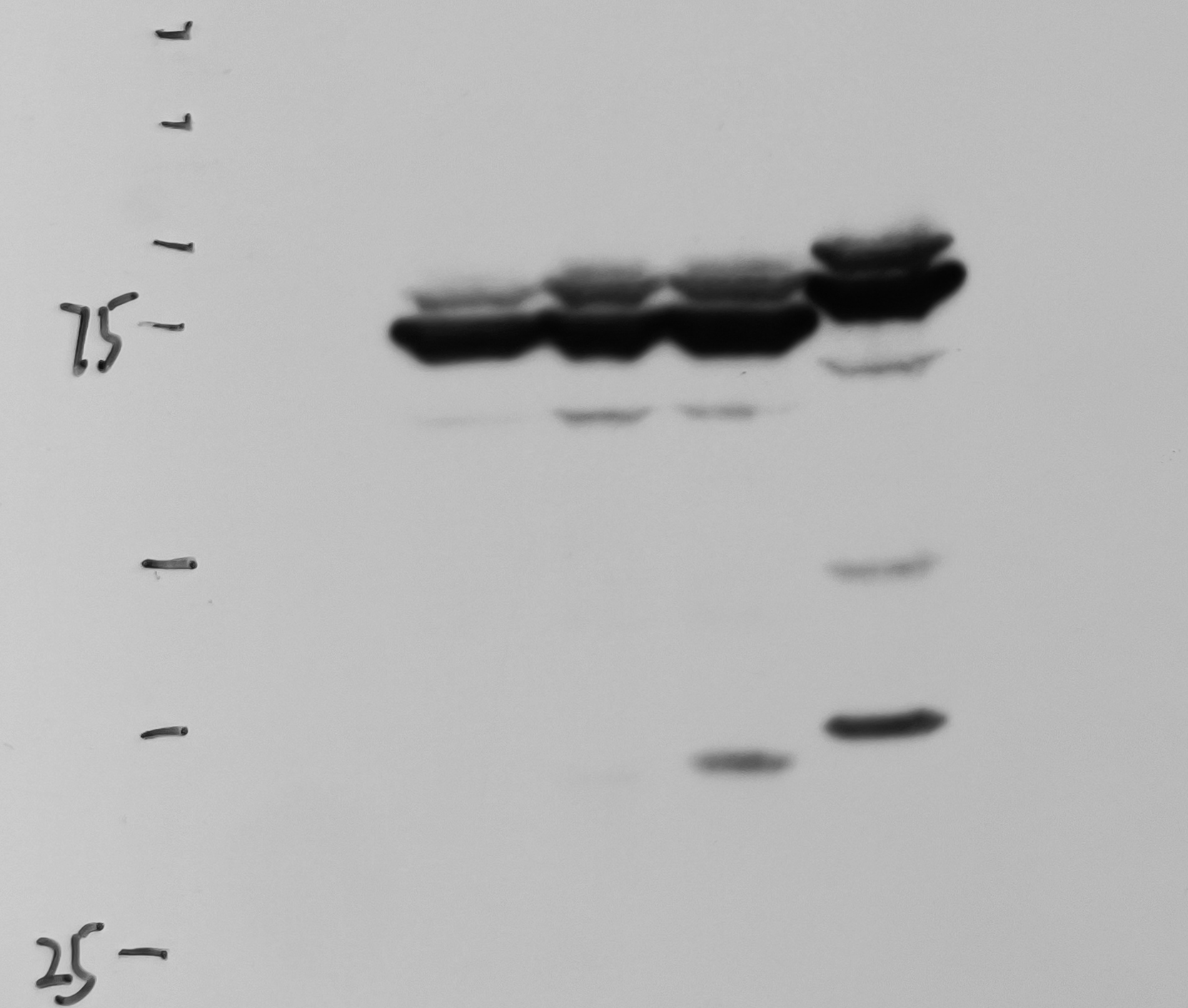

Supplement: Figure 3—source data 2. [file elife-102667-fig3-data2.zip › Figure 3-source data 2/Fig. 3. A2.tif]

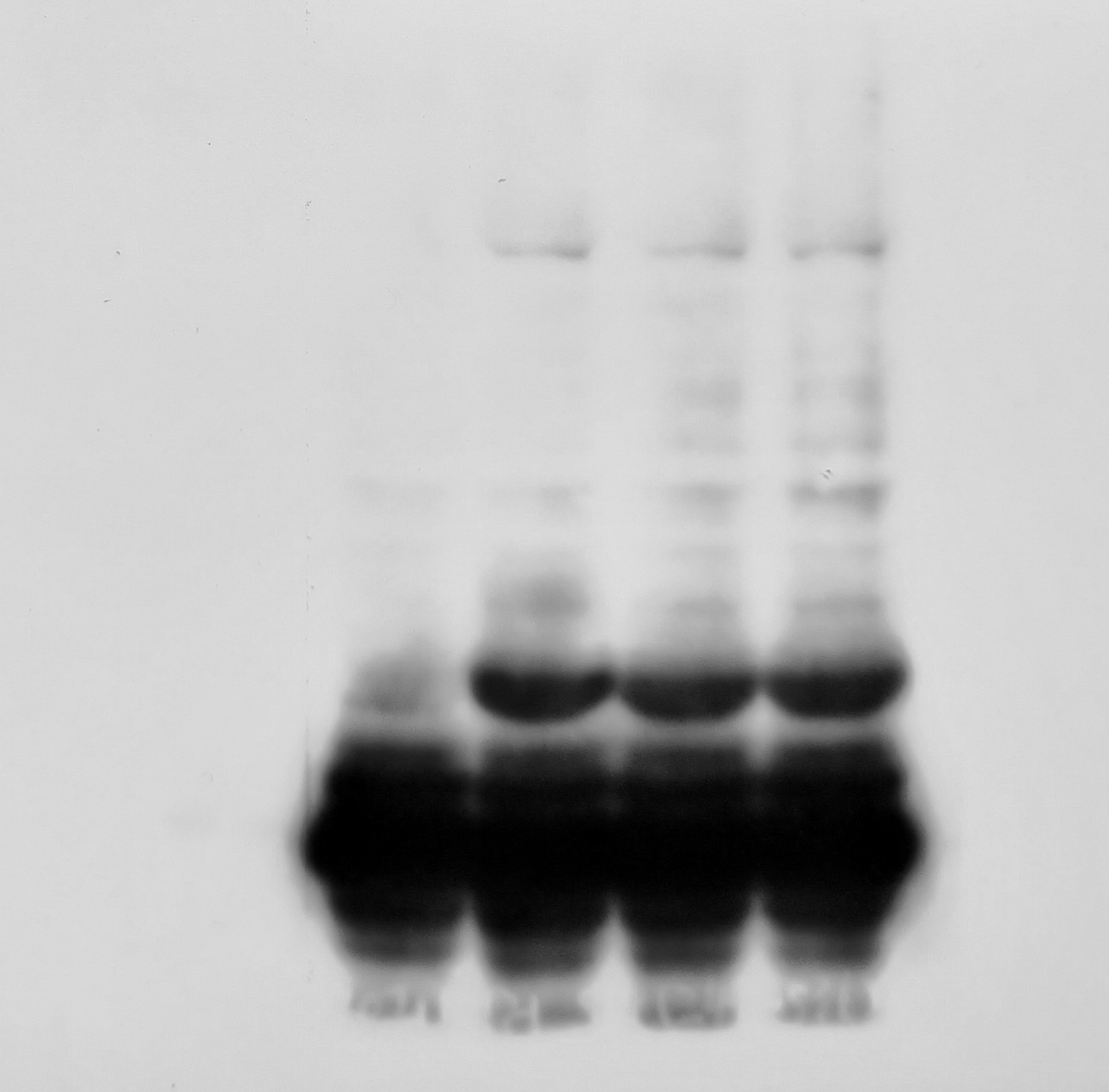

Supplement: Figure 3—source data 2. [file elife-102667-fig3-data2.zip › Figure 3-source data 2/Fig. 3. B1.tif]

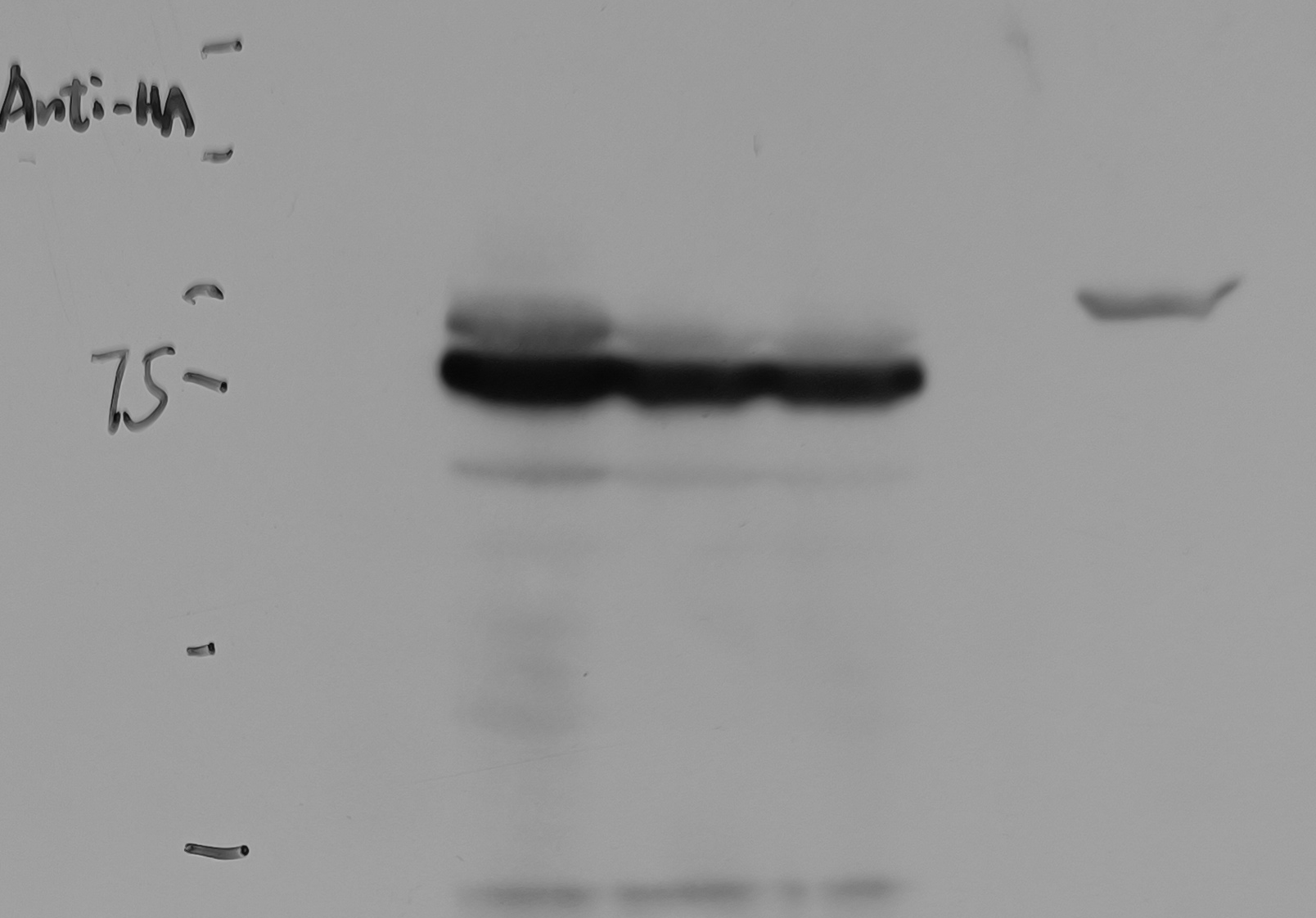

Supplement: Figure 3—source data 2. [file elife-102667-fig3-data2.zip › Figure 3-source data 2/Fig. 3. B2.tif]

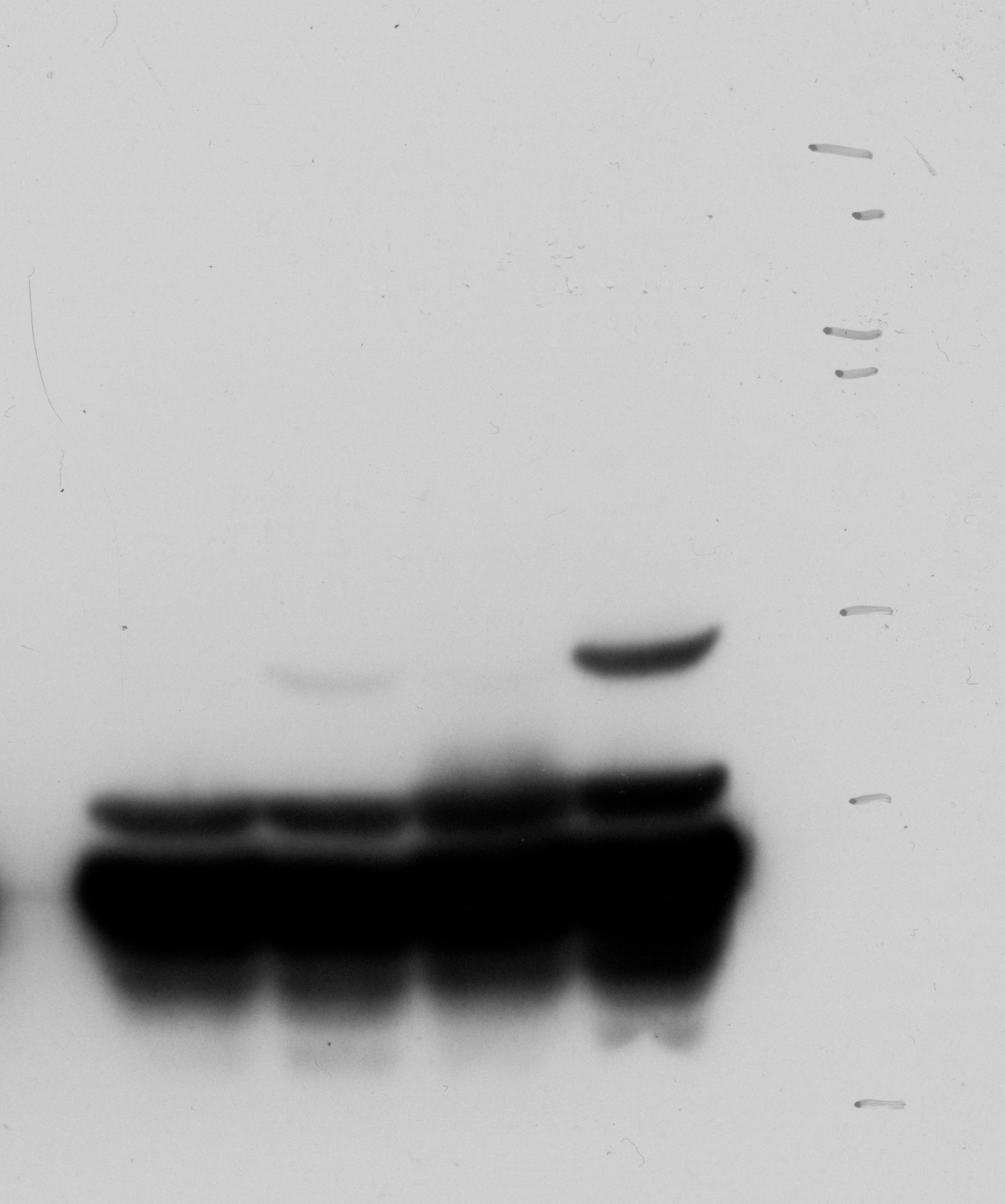

Supplement: Figure 3—source data 2. [file elife-102667-fig3-data2.zip › Figure 3-source data 2/Fig. 3. C1.tif]

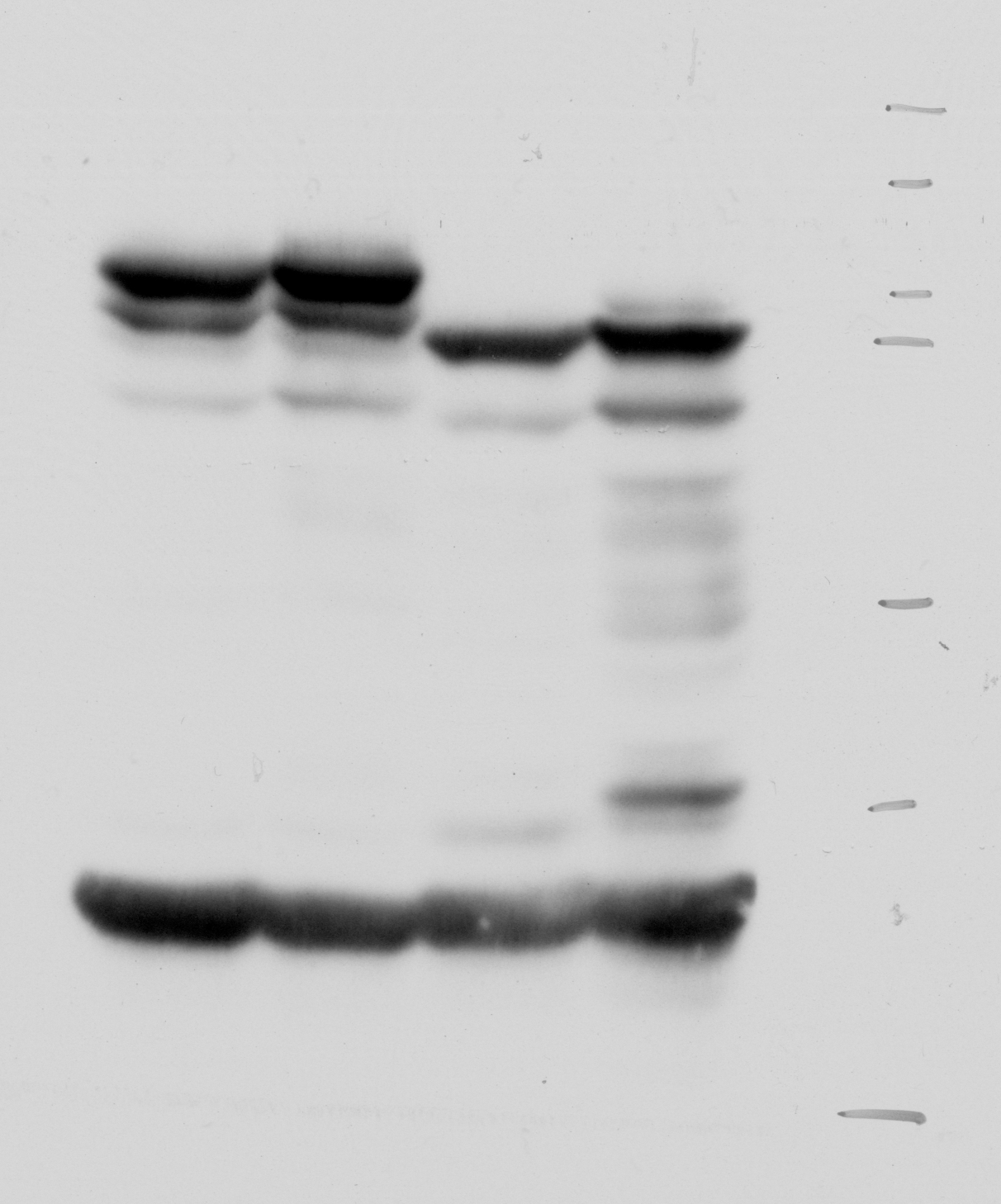

Supplement: Figure 3—source data 2. [file elife-102667-fig3-data2.zip › Figure 3-source data 2/Fig. 3. C2.tif]

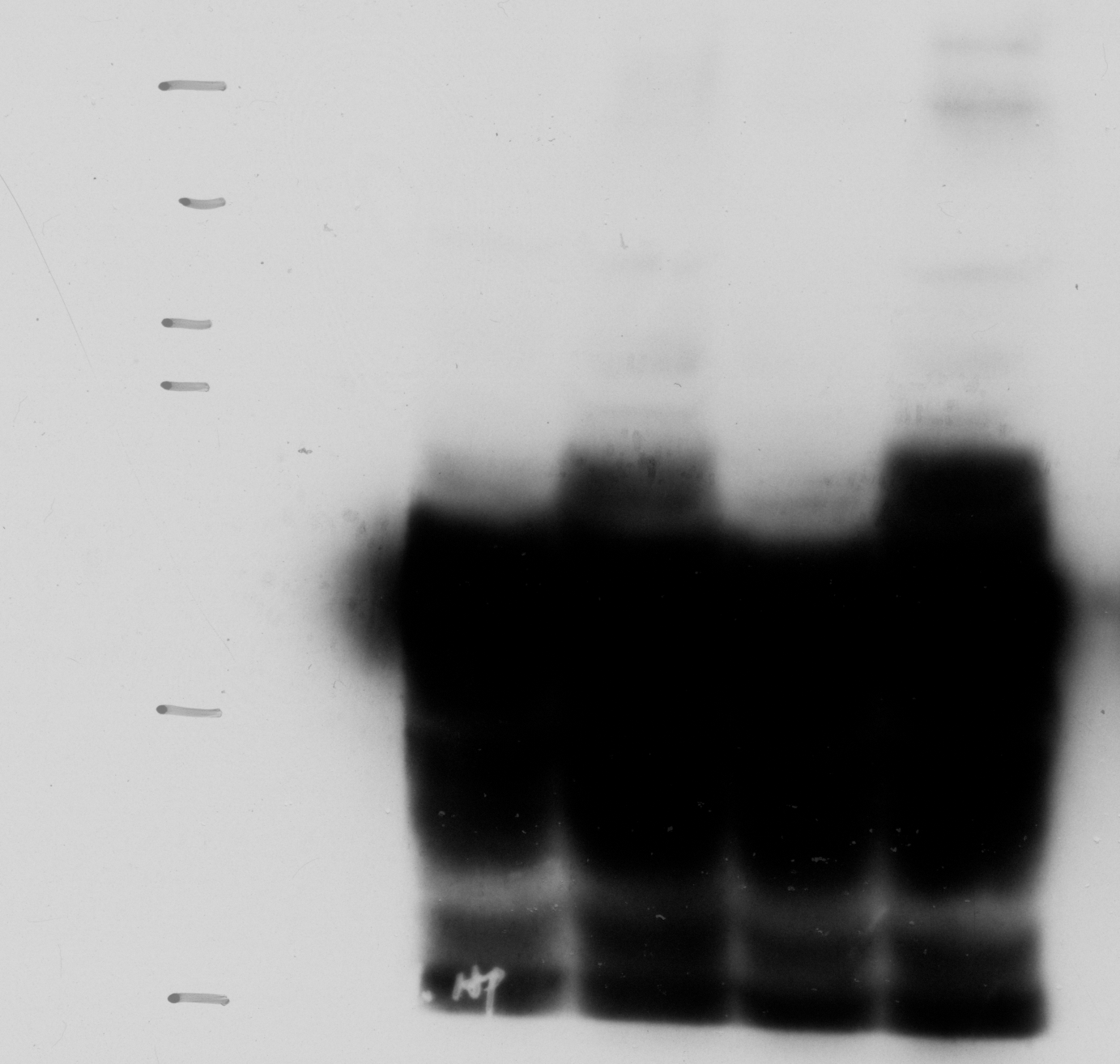

Supplement: Figure 3—source data 2. [file elife-102667-fig3-data2.zip › Figure 3-source data 2/Fig. 3. D1.tif]

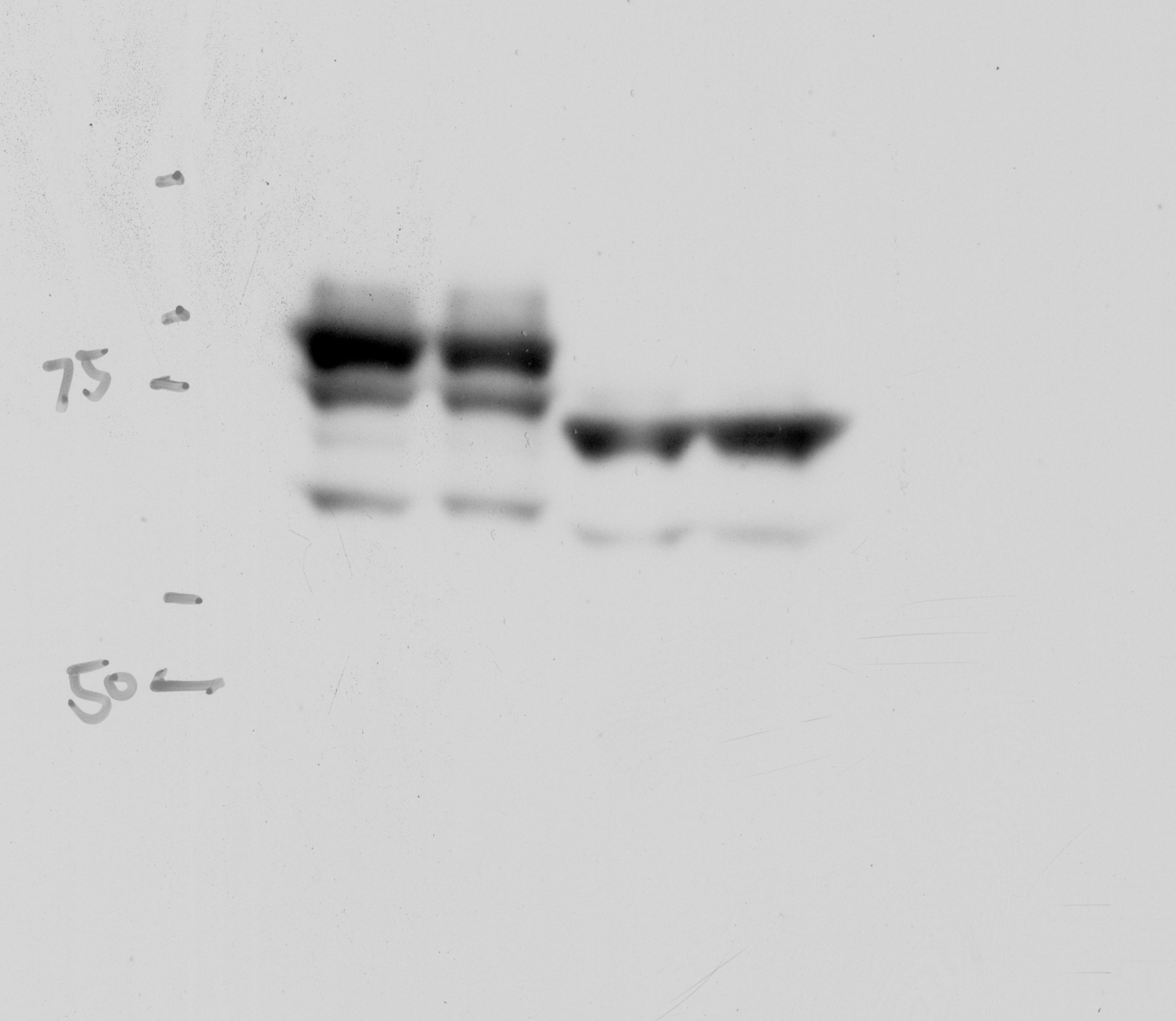

Supplement: Figure 3—source data 2. [file elife-102667-fig3-data2.zip › Figure 3-source data 2/Fig. 3. D2.tif]

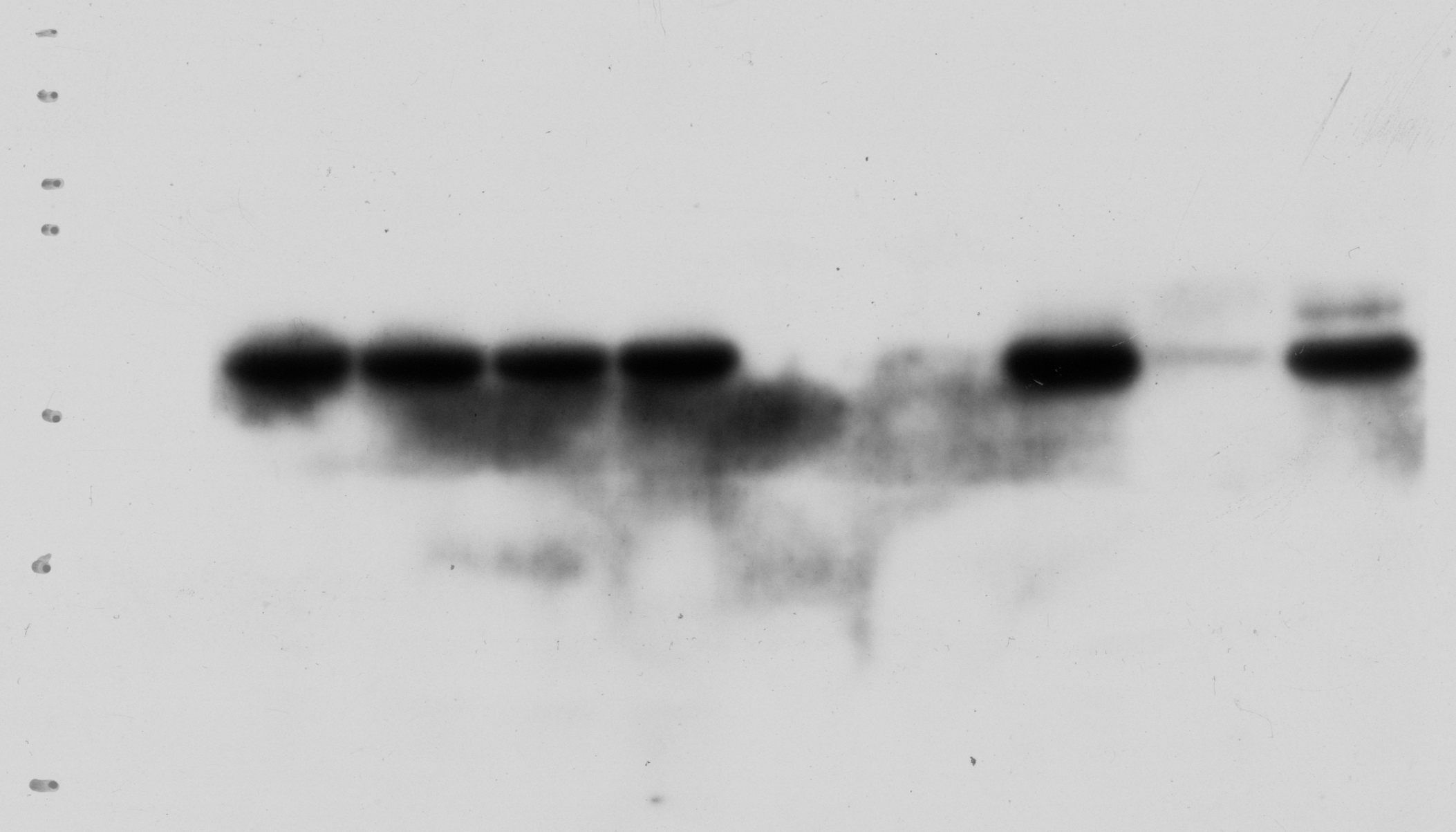

Supplement: Figure 3—source data 2. [file elife-102667-fig3-data2.zip › Figure 3-source data 2/Fig. 3. E1.tif]

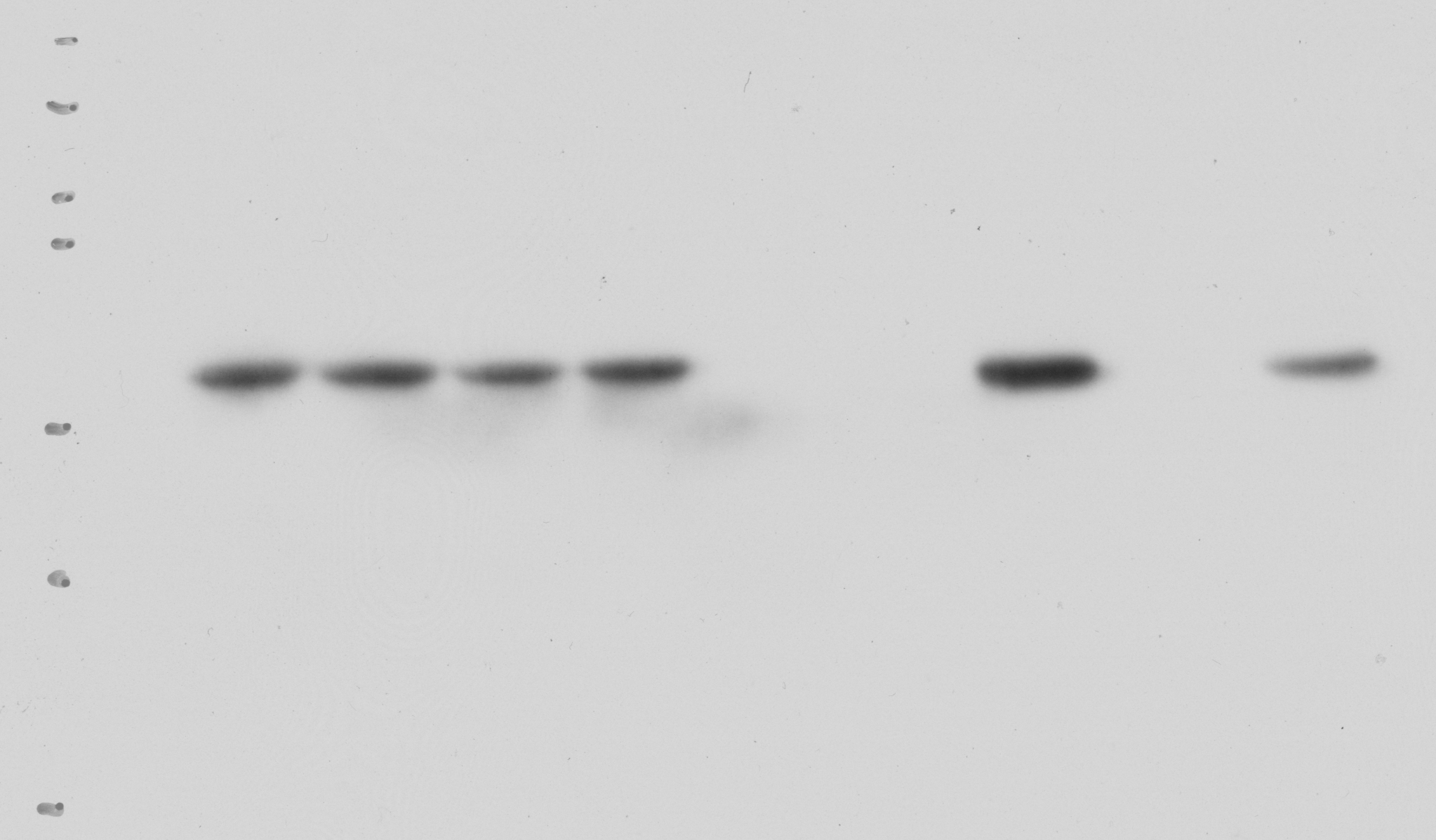

Supplement: Figure 3—source data 2. [file elife-102667-fig3-data2.zip › Figure 3-source data 2/Fig. 3. E2.tif]

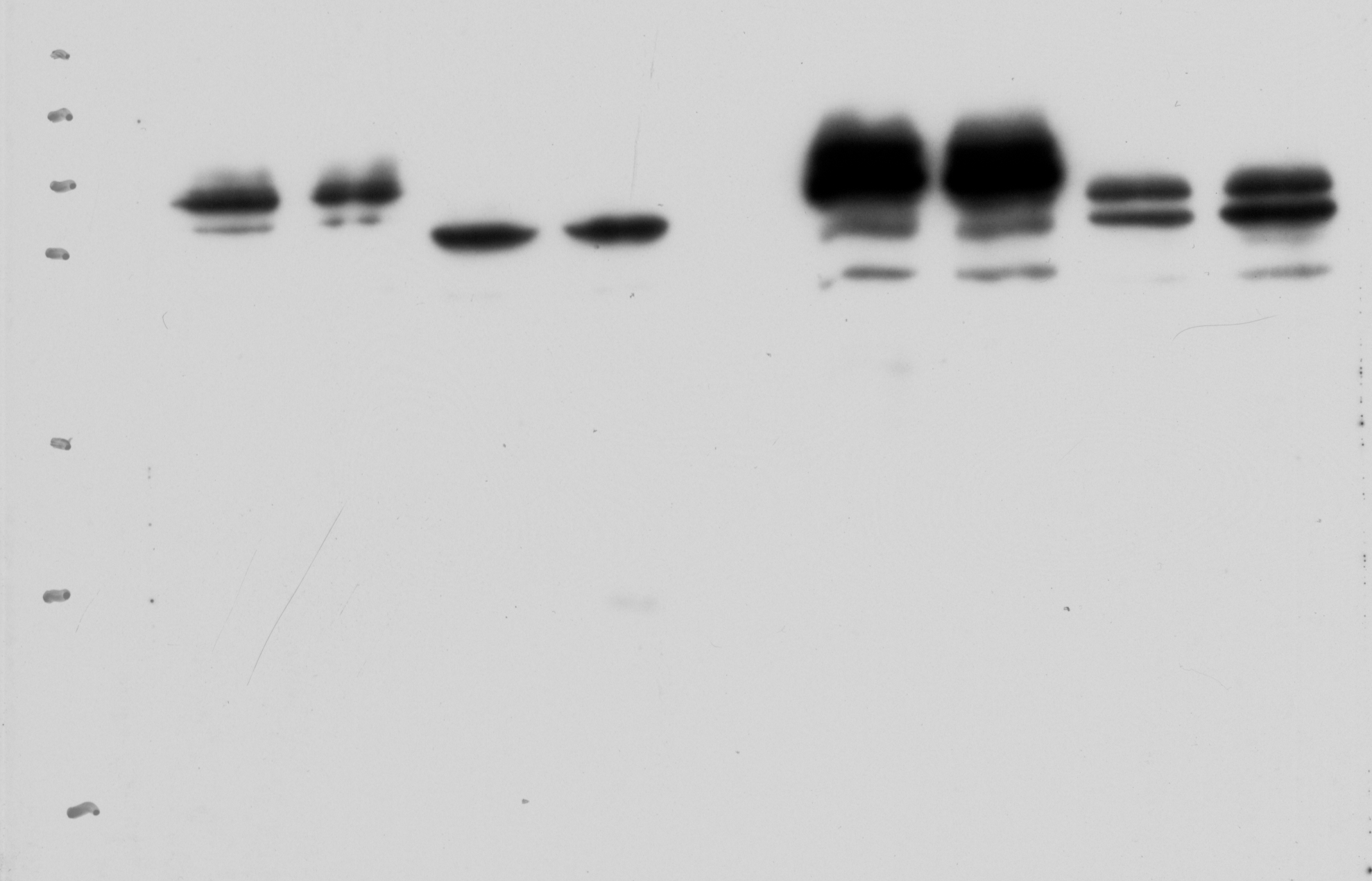

Supplement: Figure 3—source data 2. [file elife-102667-fig3-data2.zip › Figure 3-source data 2/Fig. 3. E3.tif]

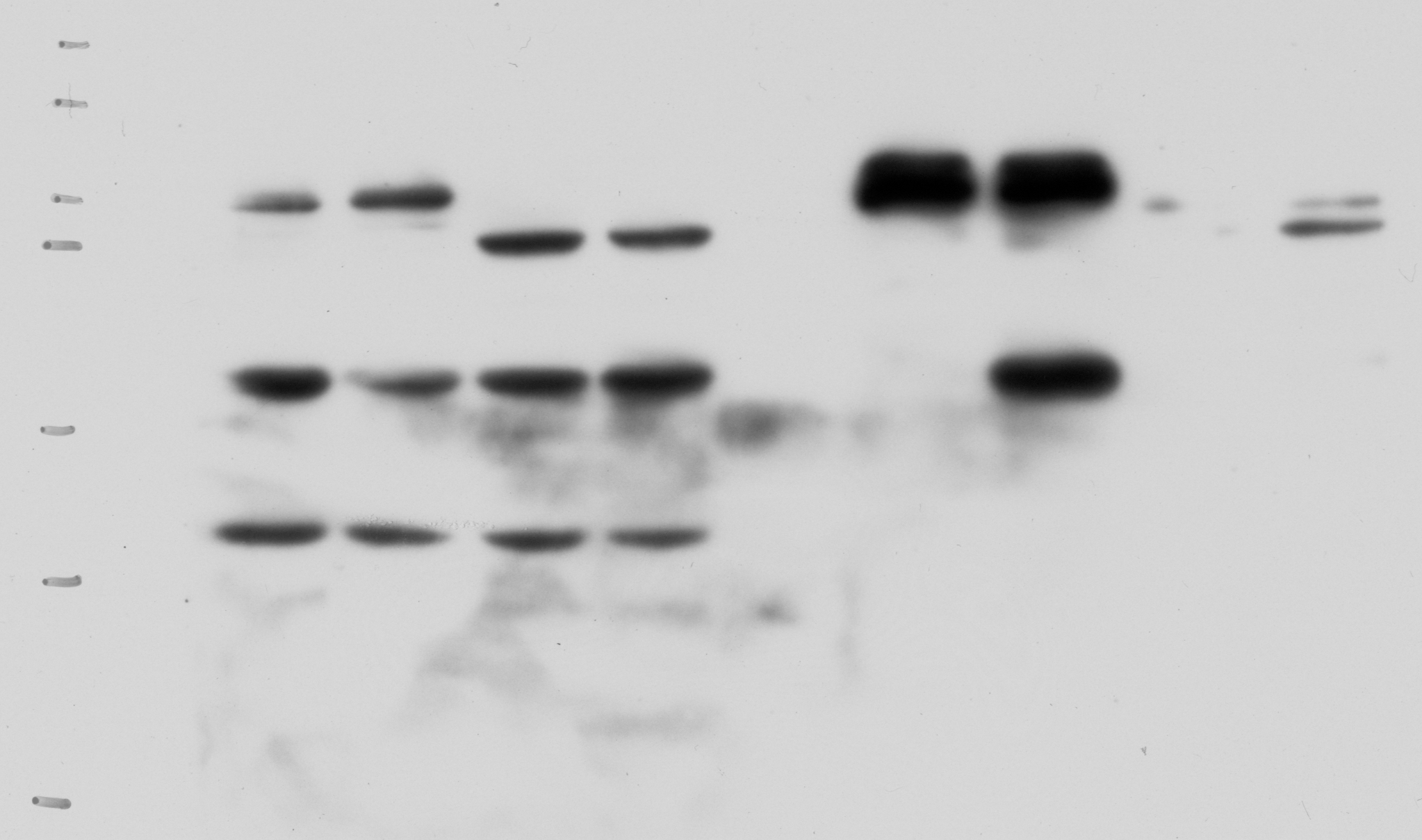

Supplement: Figure 3—source data 2. [file elife-102667-fig3-data2.zip › Figure 3-source data 2/Fig. 3. E4.tif]

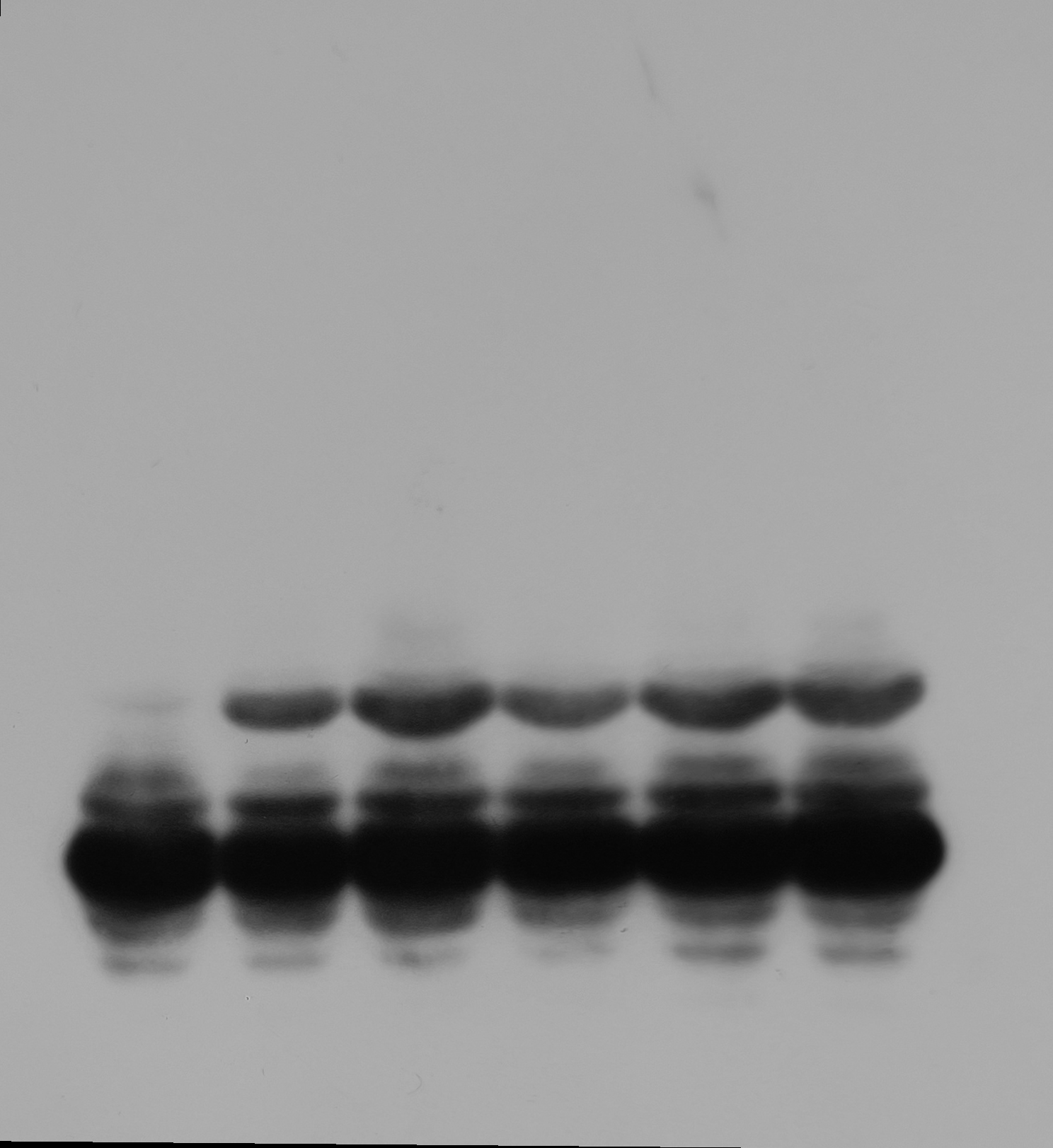

Supplement: Figure 3—source data 2. [file elife-102667-fig3-data2.zip › Figure 3-source data 2/Fig. 3. F1.tif]

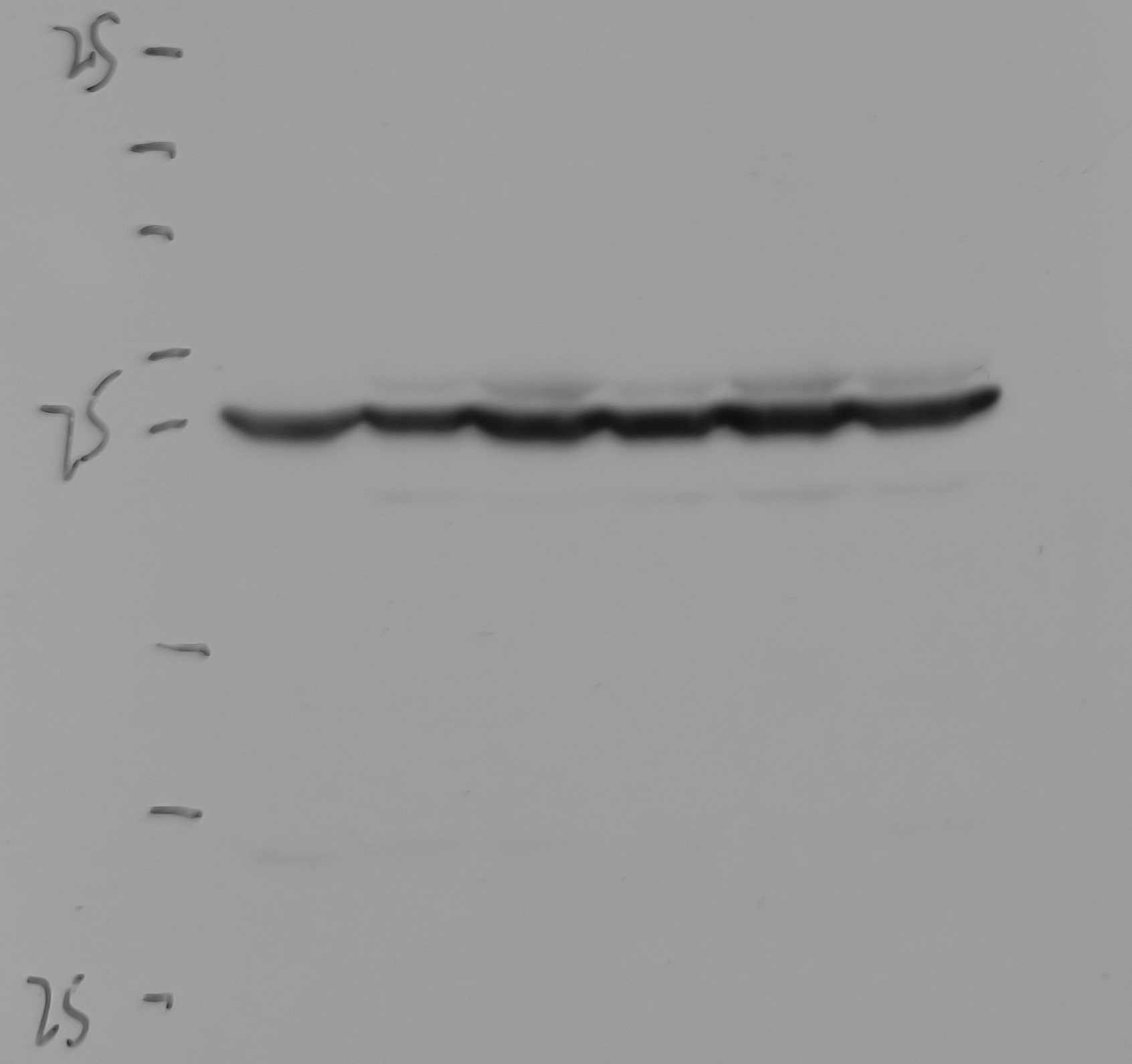

Supplement: Figure 3—source data 2. [file elife-102667-fig3-data2.zip › Figure 3-source data 2/Fig. 3. F2.tif]

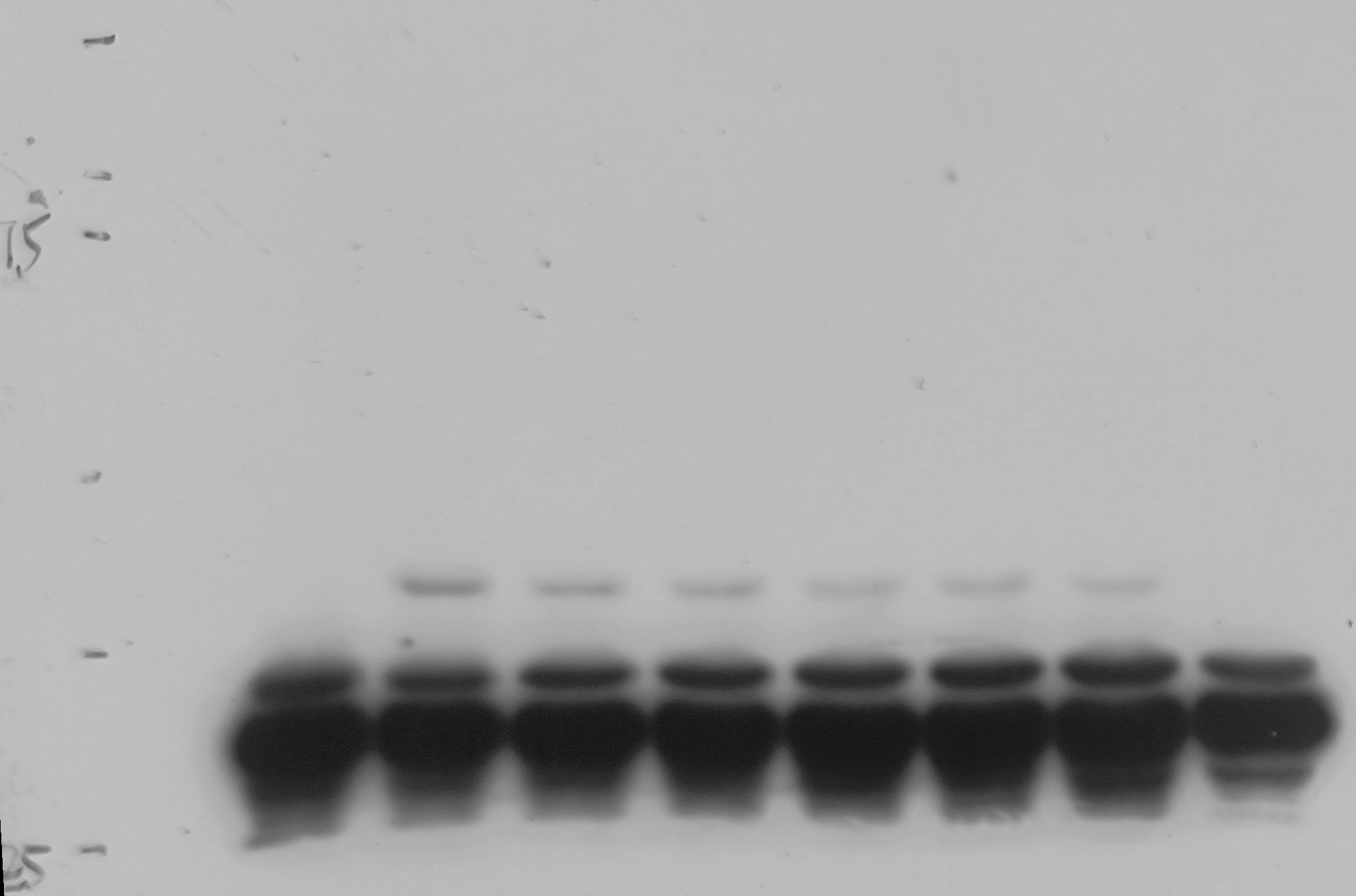

Supplement: Figure 3—source data 2. [file elife-102667-fig3-data2.zip › Figure 3-source data 2/Fig. 3. G1.tif]

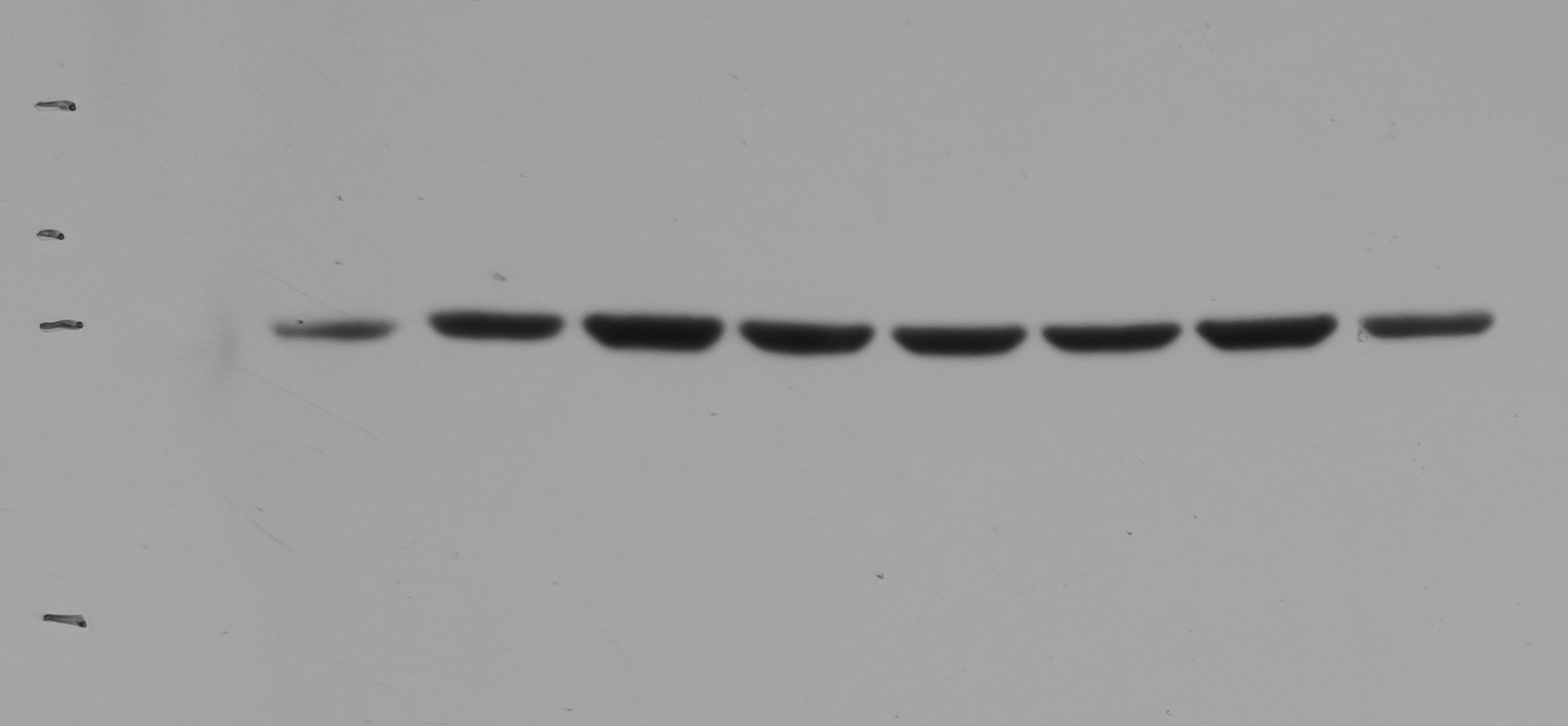

Supplement: Figure 3—source data 2. [file elife-102667-fig3-data2.zip › Figure 3-source data 2/Fig. 3. G2.tif]

**Figure 3—figure supplement 1 B**

**B**

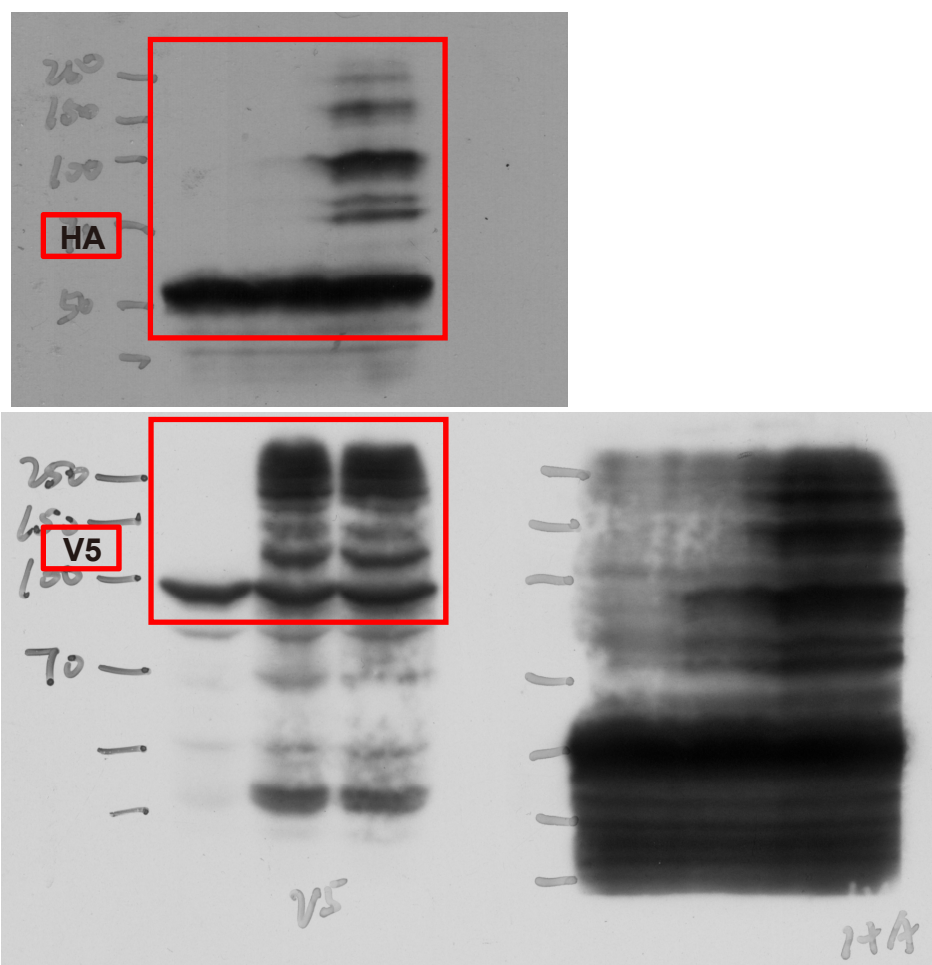

**Figure 3—figure supplement 1 C and D**

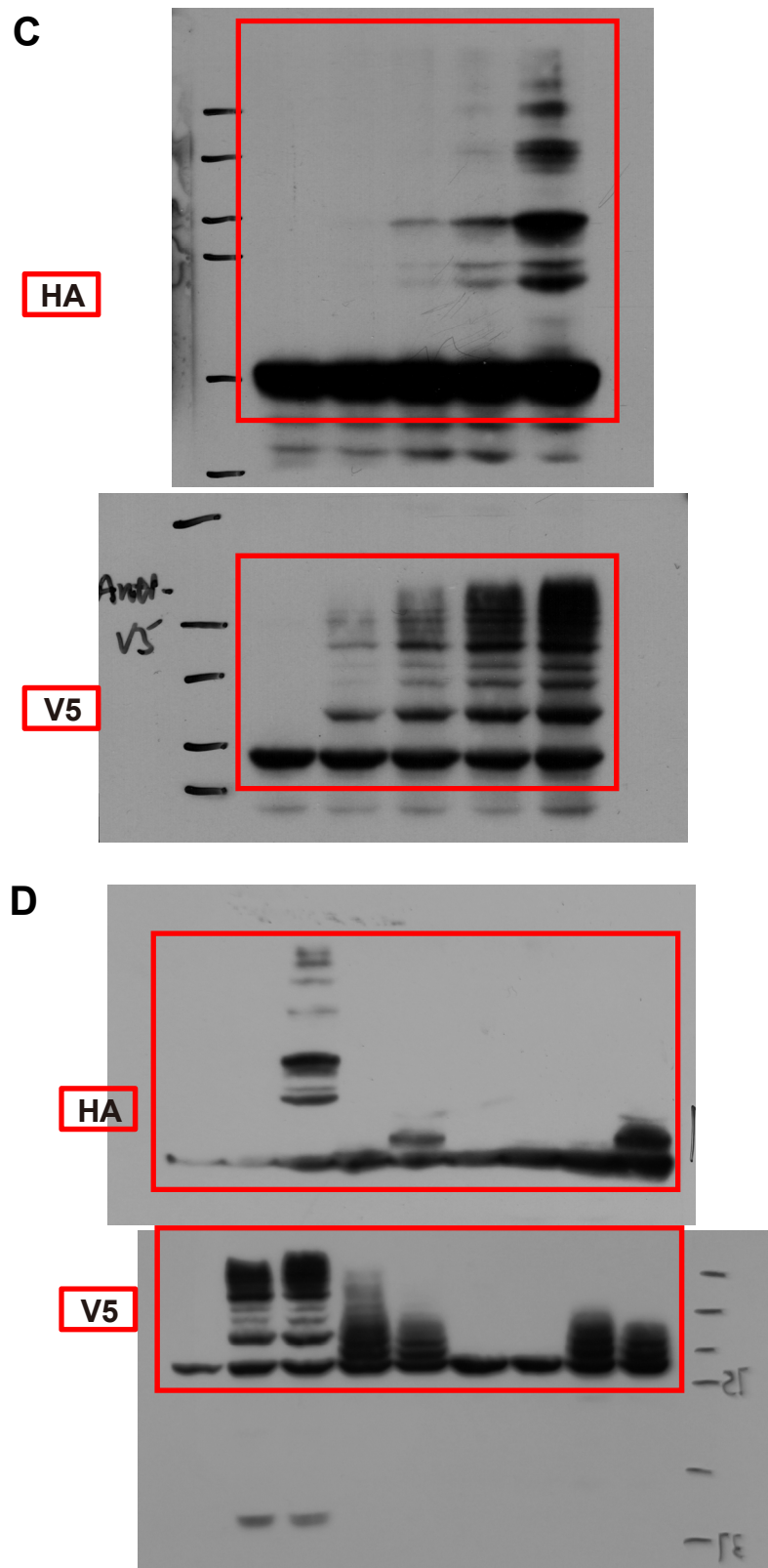

Supplement: Figure 3—figure supplement 1—source data 1. [file elife-102667-fig3-figsupp1-data1.zip › Figure 3—figure supplement 1-source data 1.pdf]

Figure 3—figure supplement 3

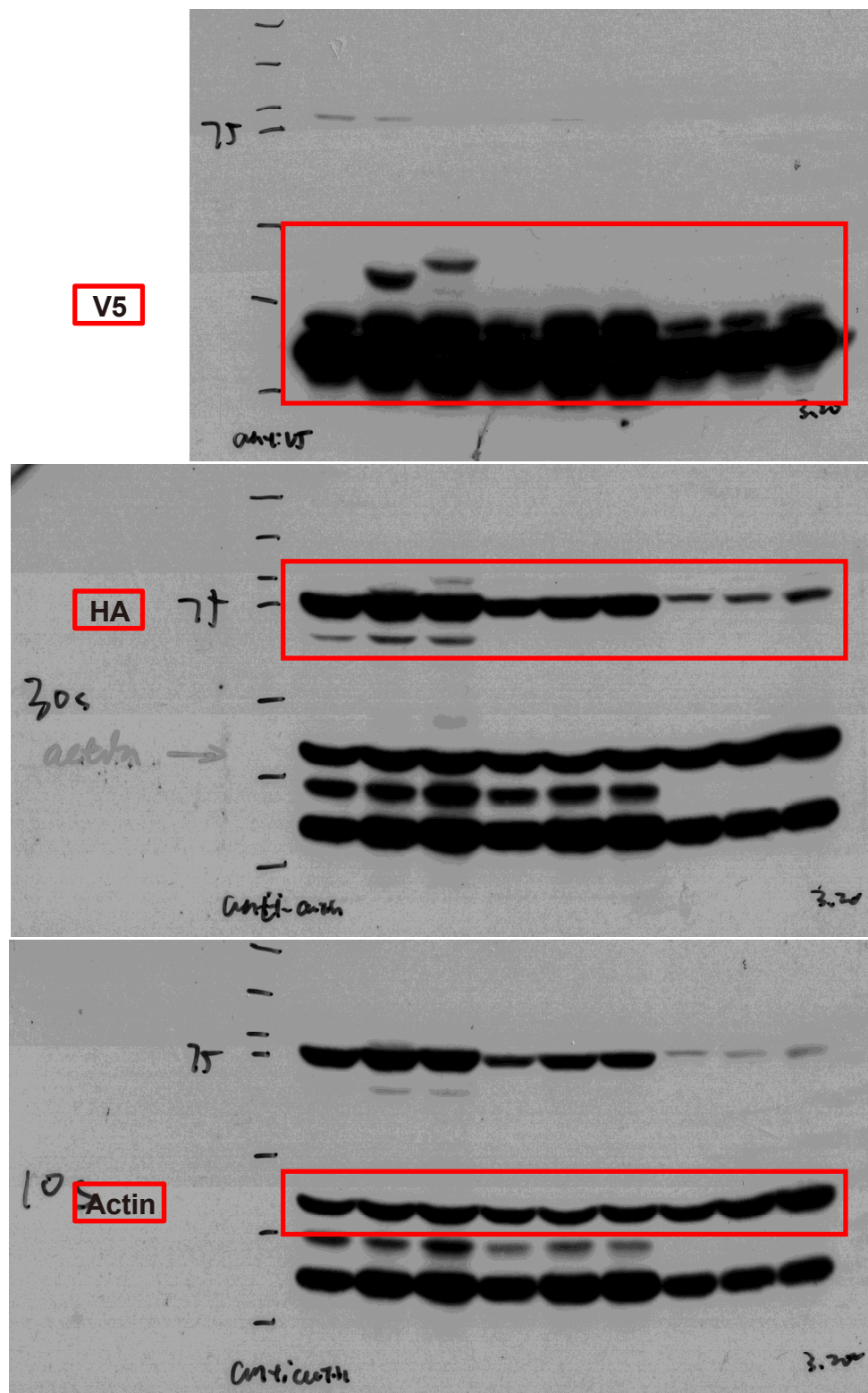

Supplement: Figure 3—figure supplement 3—source data 1. [file elife-102667-fig3-figsupp3-data1.zip › Figure 3—figure supplement 3-source data 1.pdf]

**Figure 4**

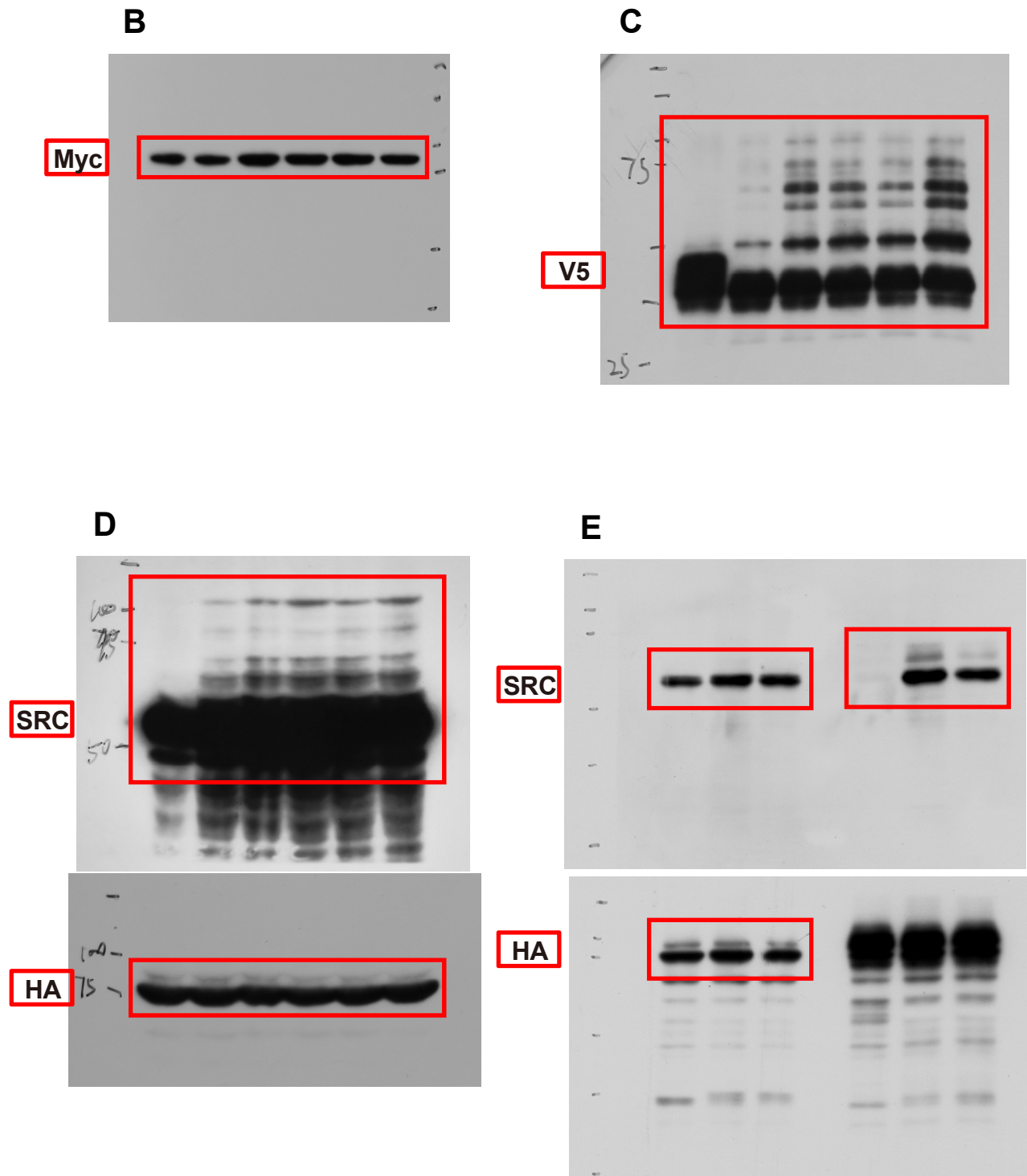

Supplement: Figure 4—source data 1. [file elife-102667-fig4-data1.zip › Figure 4-source data 1.pdf]

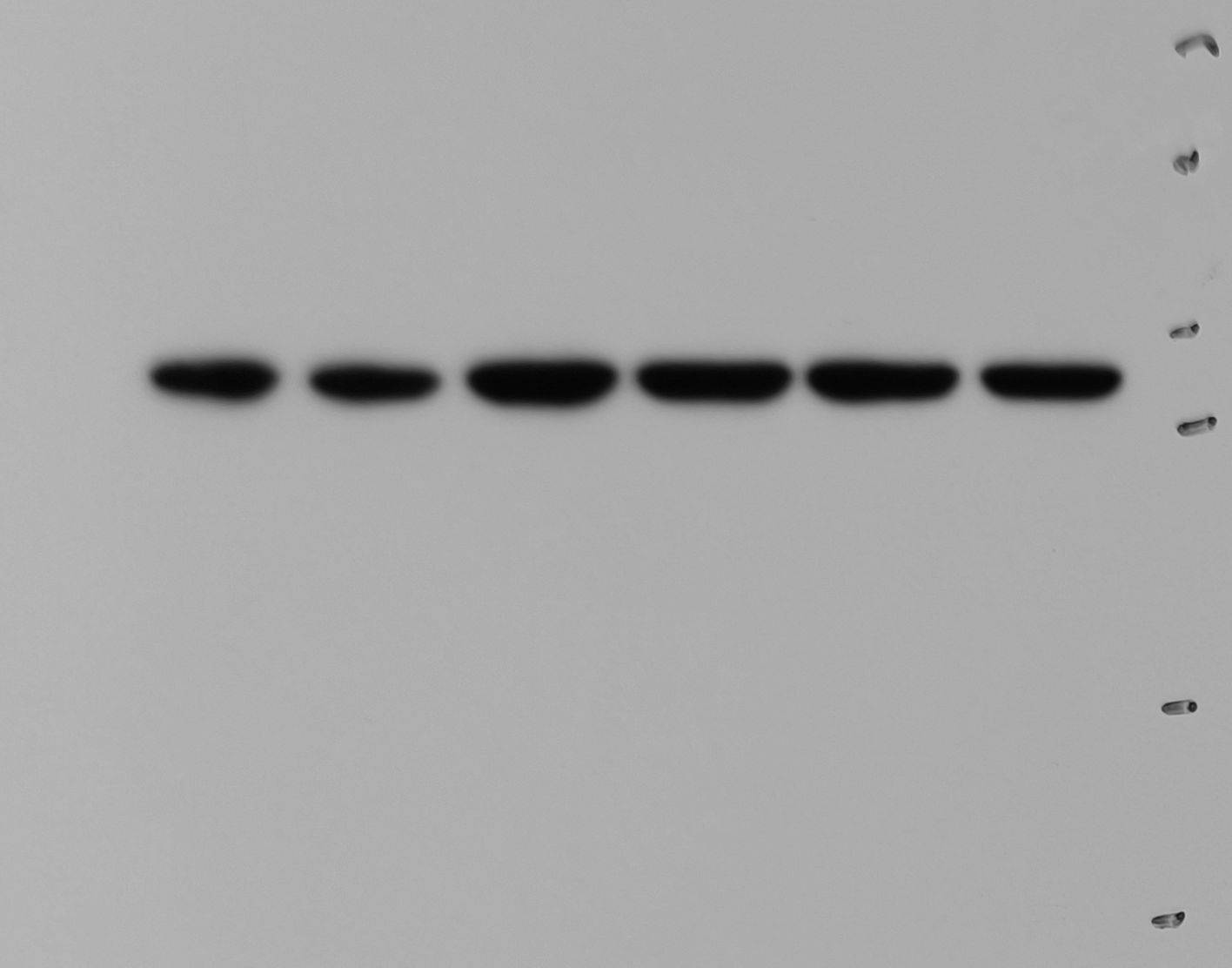

Supplement: Figure 4—source data 2. [file elife-102667-fig4-data2.zip › Figure 4-source data 2/Fig. 4. B.tif]

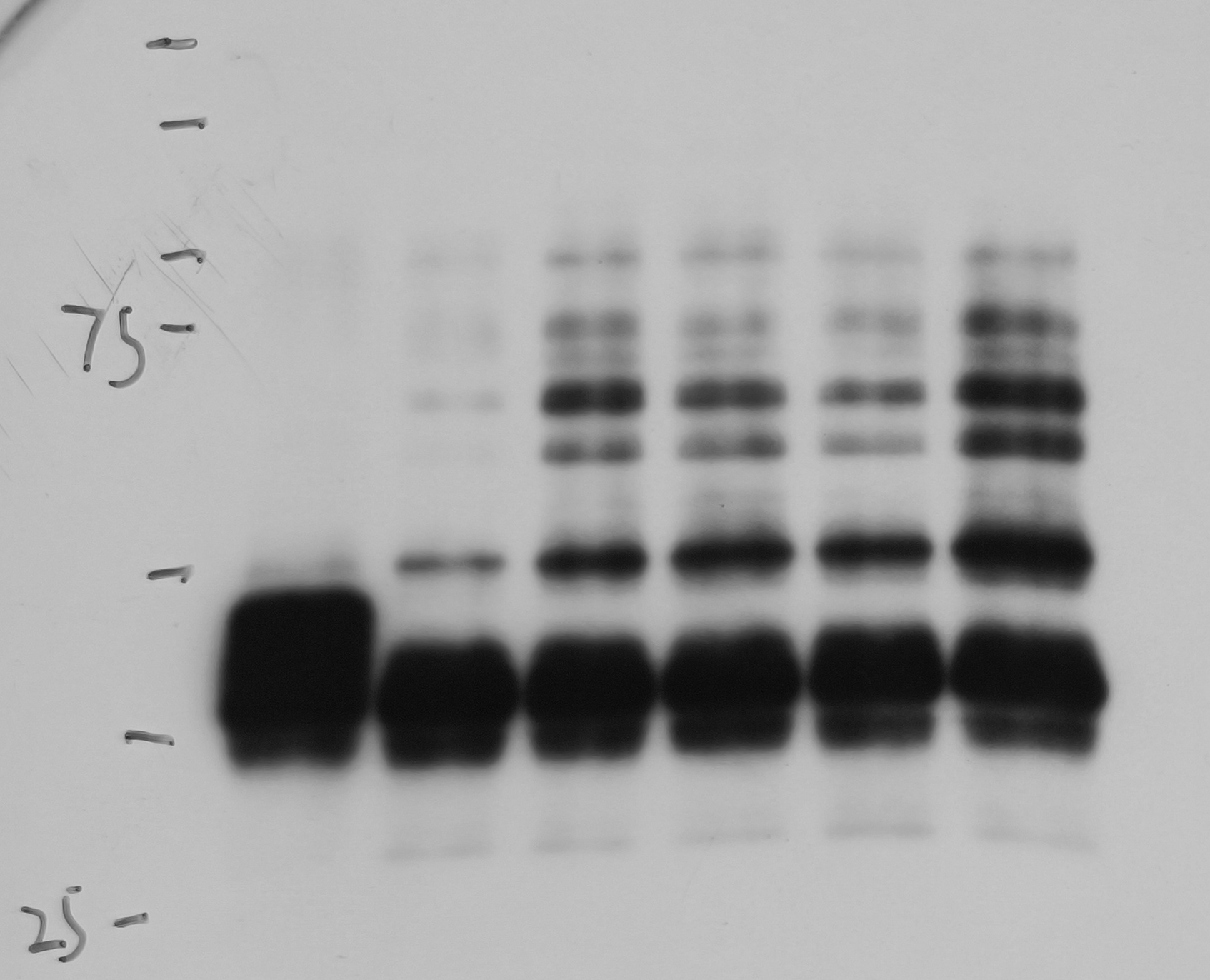

Supplement: Figure 4—source data 2. [file elife-102667-fig4-data2.zip › Figure 4-source data 2/Fig. 4. C.tif]

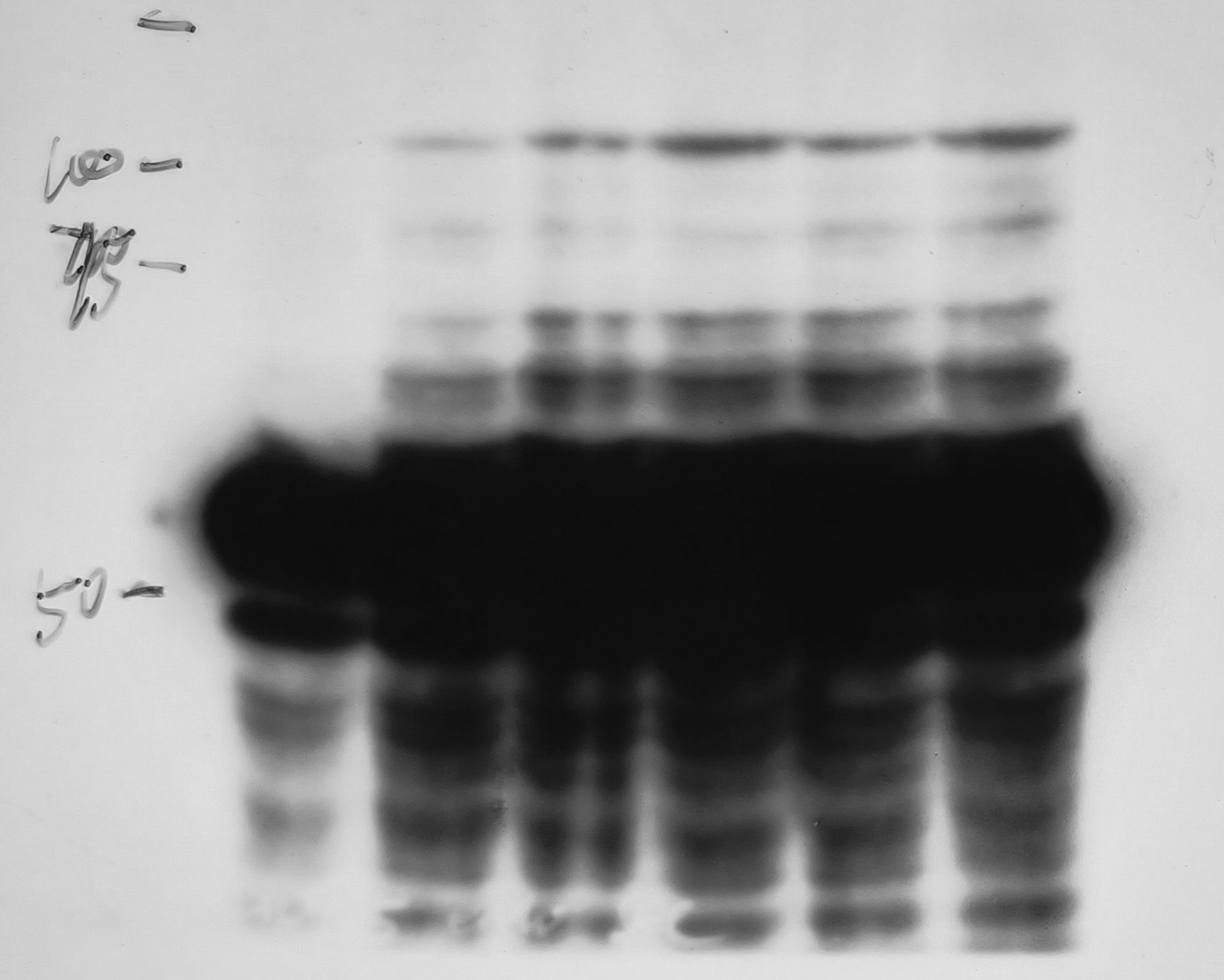

Supplement: Figure 4—source data 2. [file elife-102667-fig4-data2.zip › Figure 4-source data 2/Fig. 4. D1.tif]

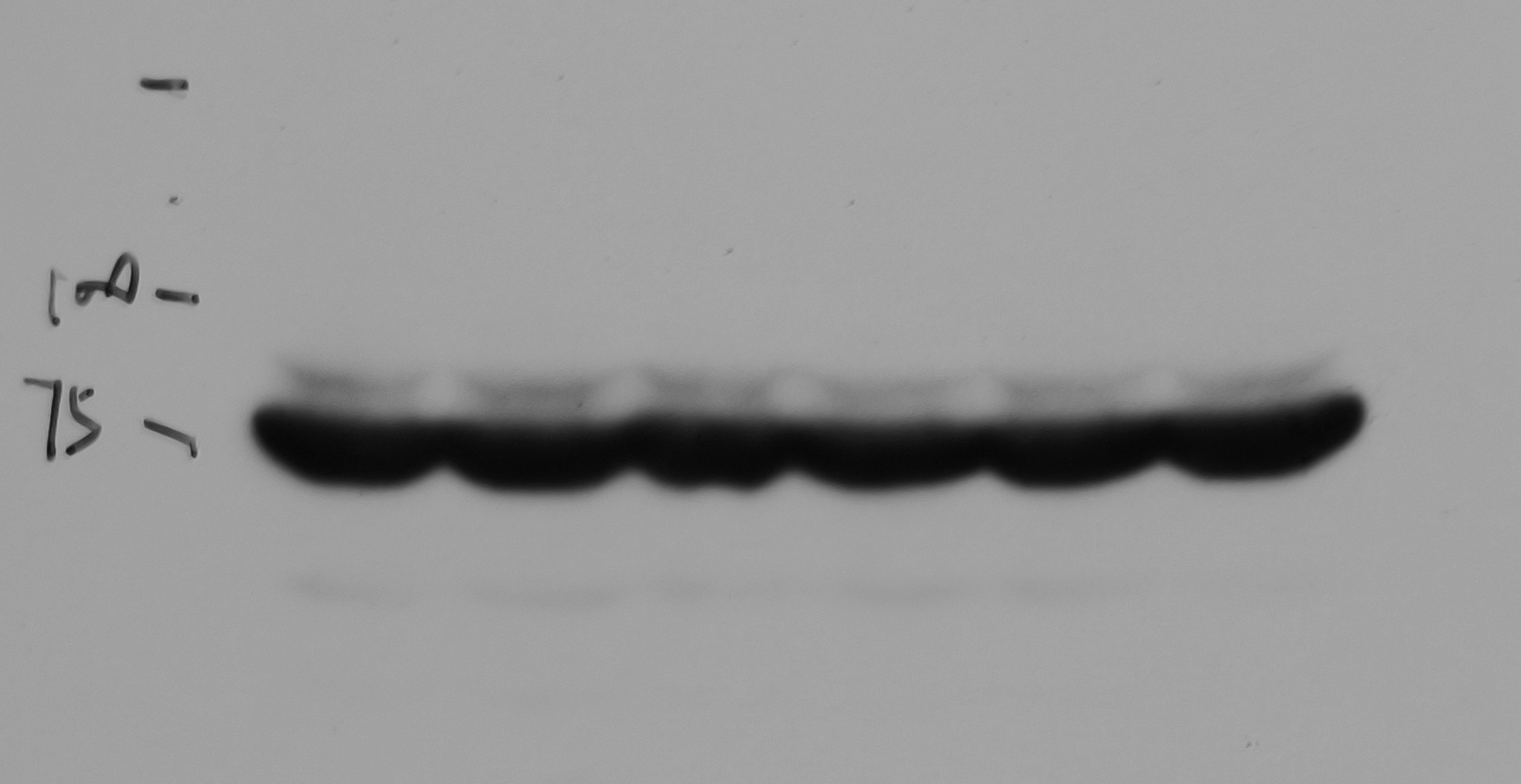

Supplement: Figure 4—source data 2. [file elife-102667-fig4-data2.zip › Figure 4-source data 2/Fig. 4. D2.tif]

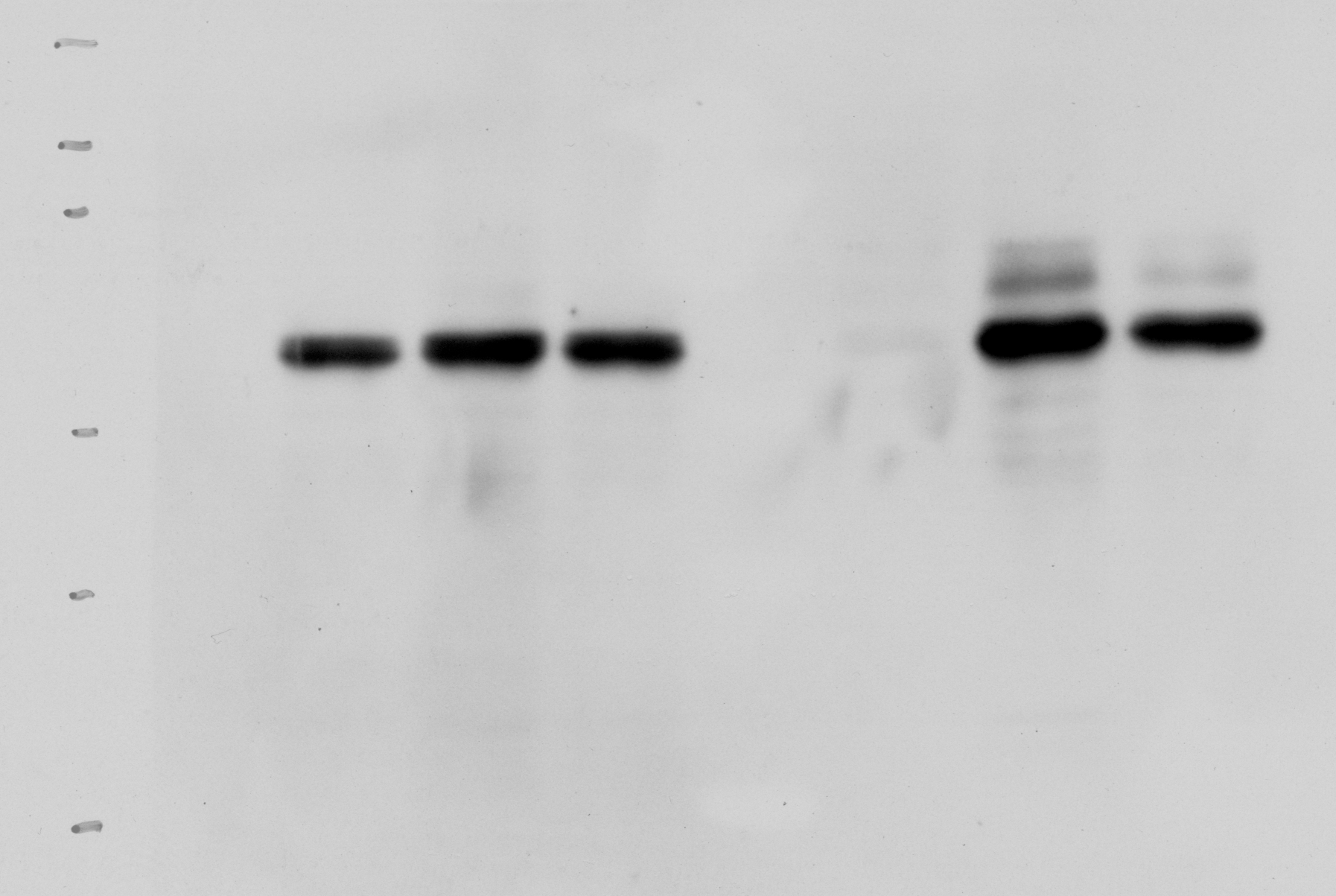

Supplement: Figure 4—source data 2. [file elife-102667-fig4-data2.zip › Figure 4-source data 2/Fig. 4. E1.tif]

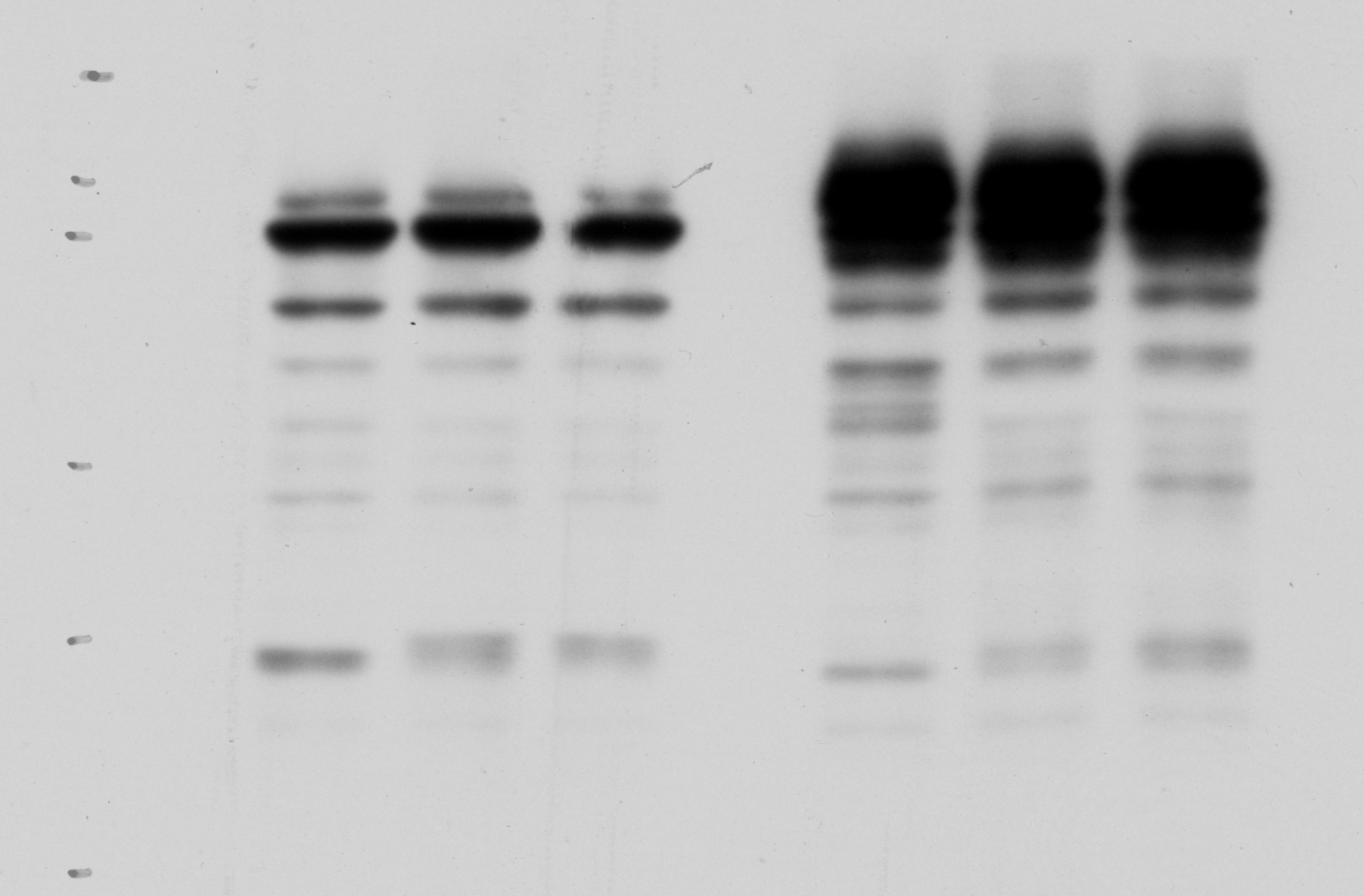

Supplement: Figure 4—source data 2. [file elife-102667-fig4-data2.zip › Figure 4-source data 2/Fig. 4. E2.tif]

**Figure 6 D and E**

**D**

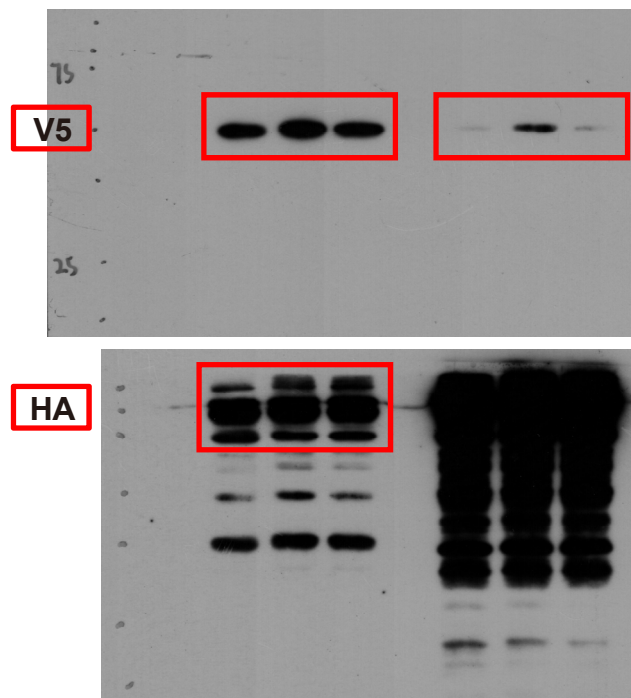

**E**

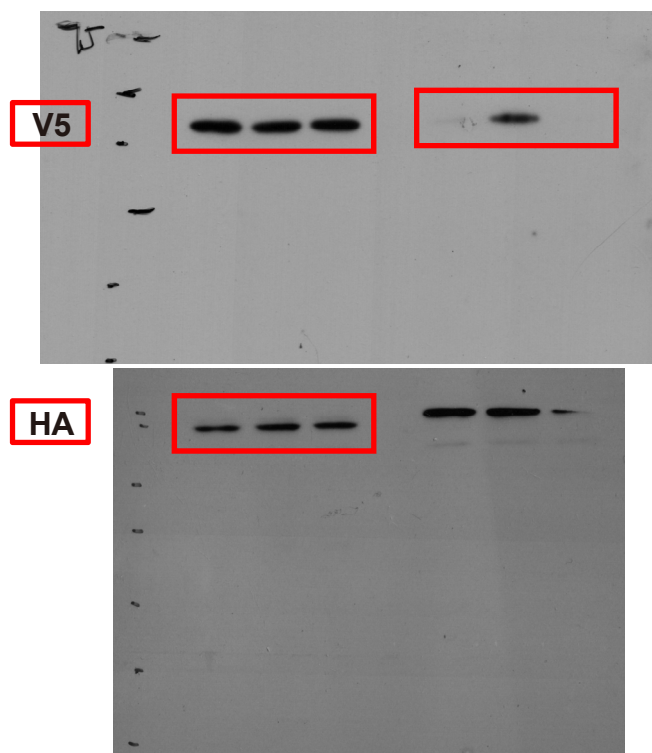

**Figure 6 F and G**

**F**

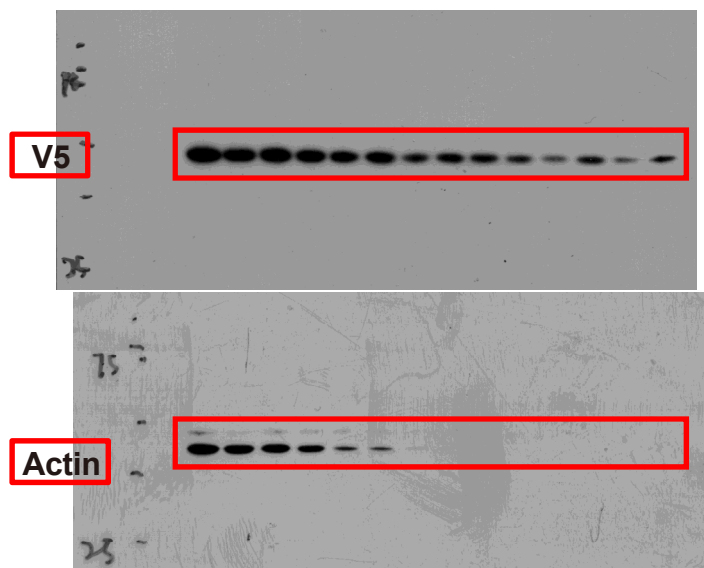

**G**

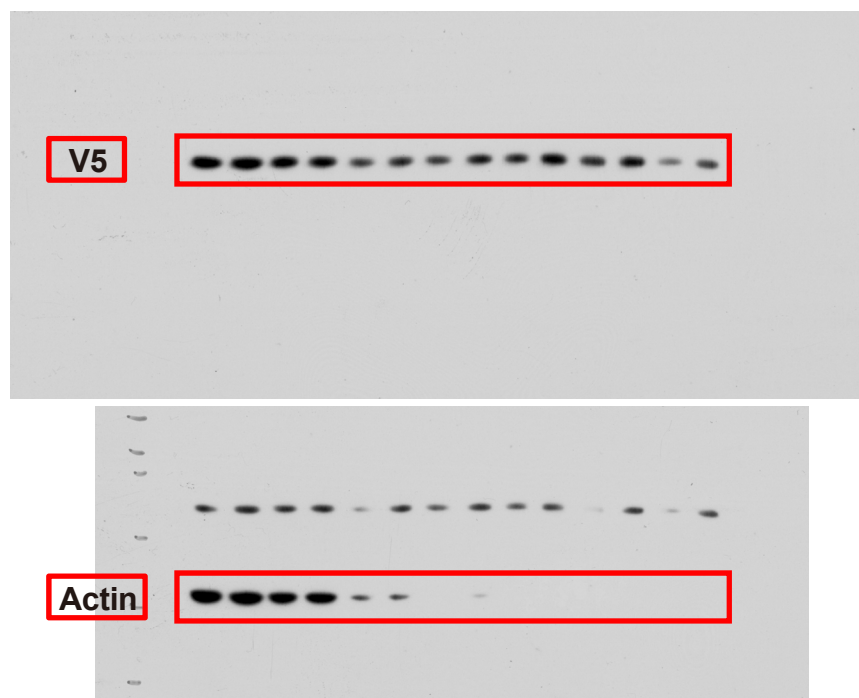

Supplement: Figure 6—source data 1. [file elife-102667-fig6-data1.zip › Figure 6-source data 1.pdf]

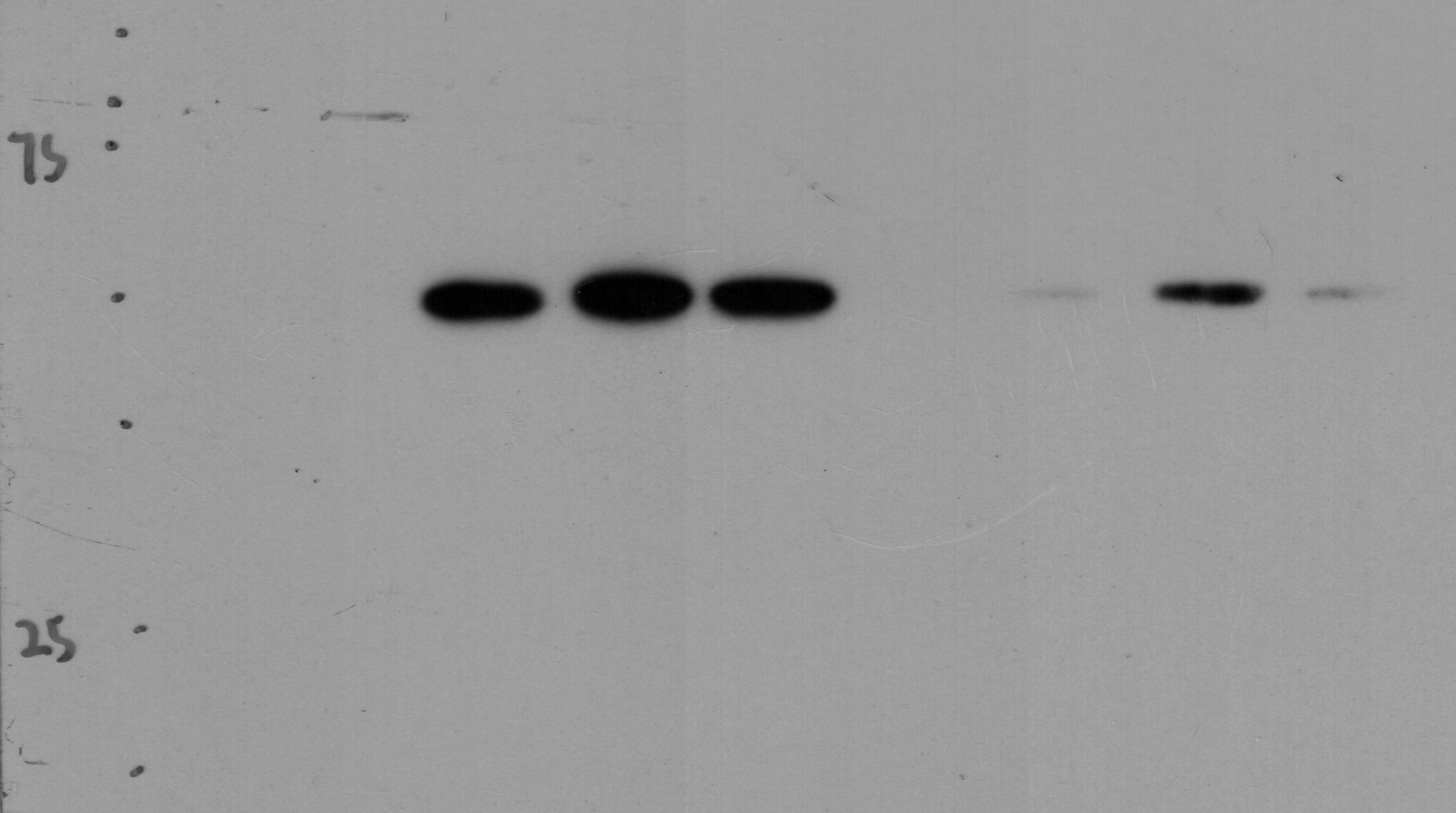

Supplement: Figure 6—source data 2. [file elife-102667-fig6-data2.zip › Figure 6-source data 2/Fig. 6. D1.tif]

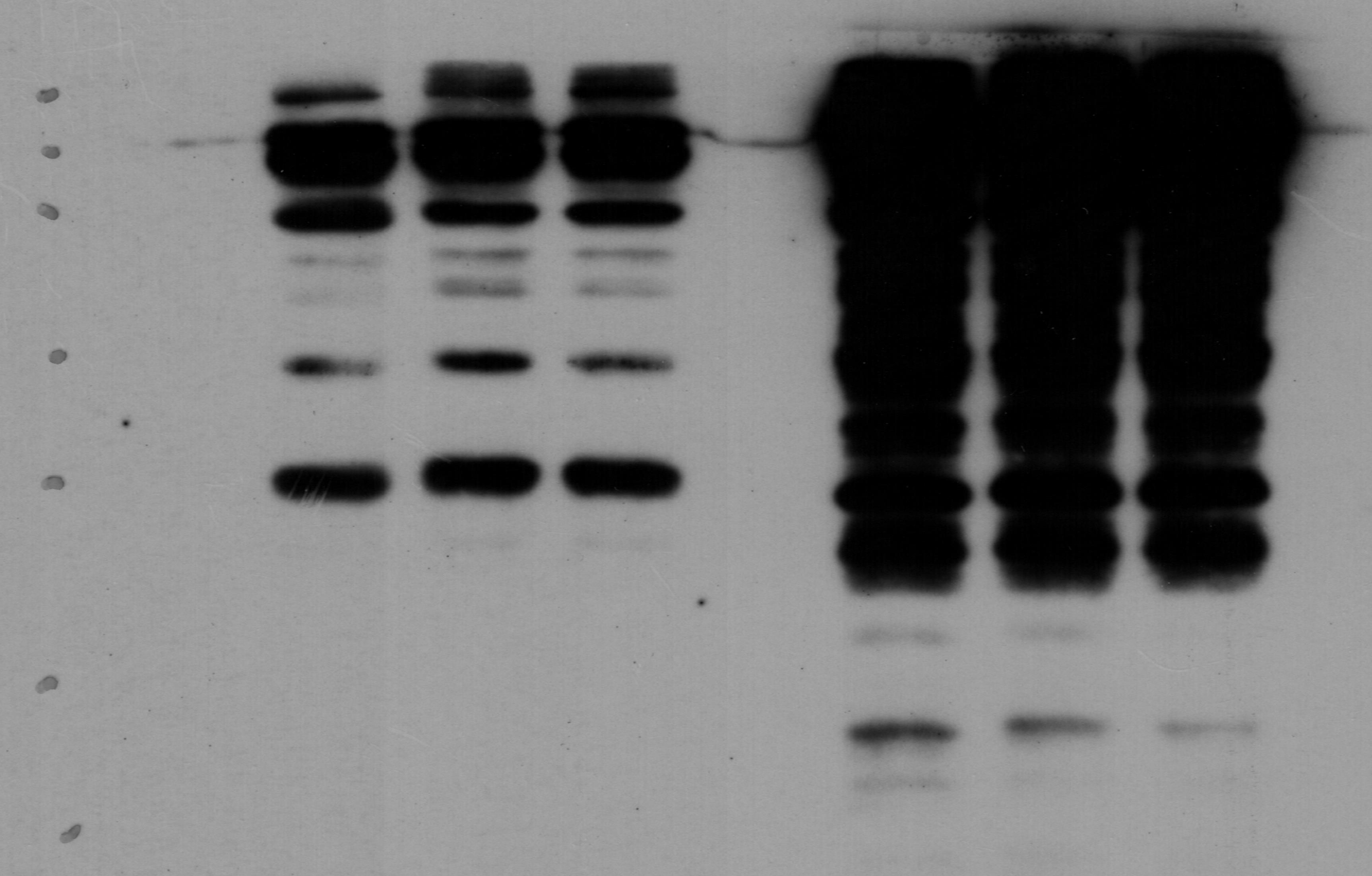

Supplement: Figure 6—source data 2. [file elife-102667-fig6-data2.zip › Figure 6-source data 2/Fig. 6. D2.tif]

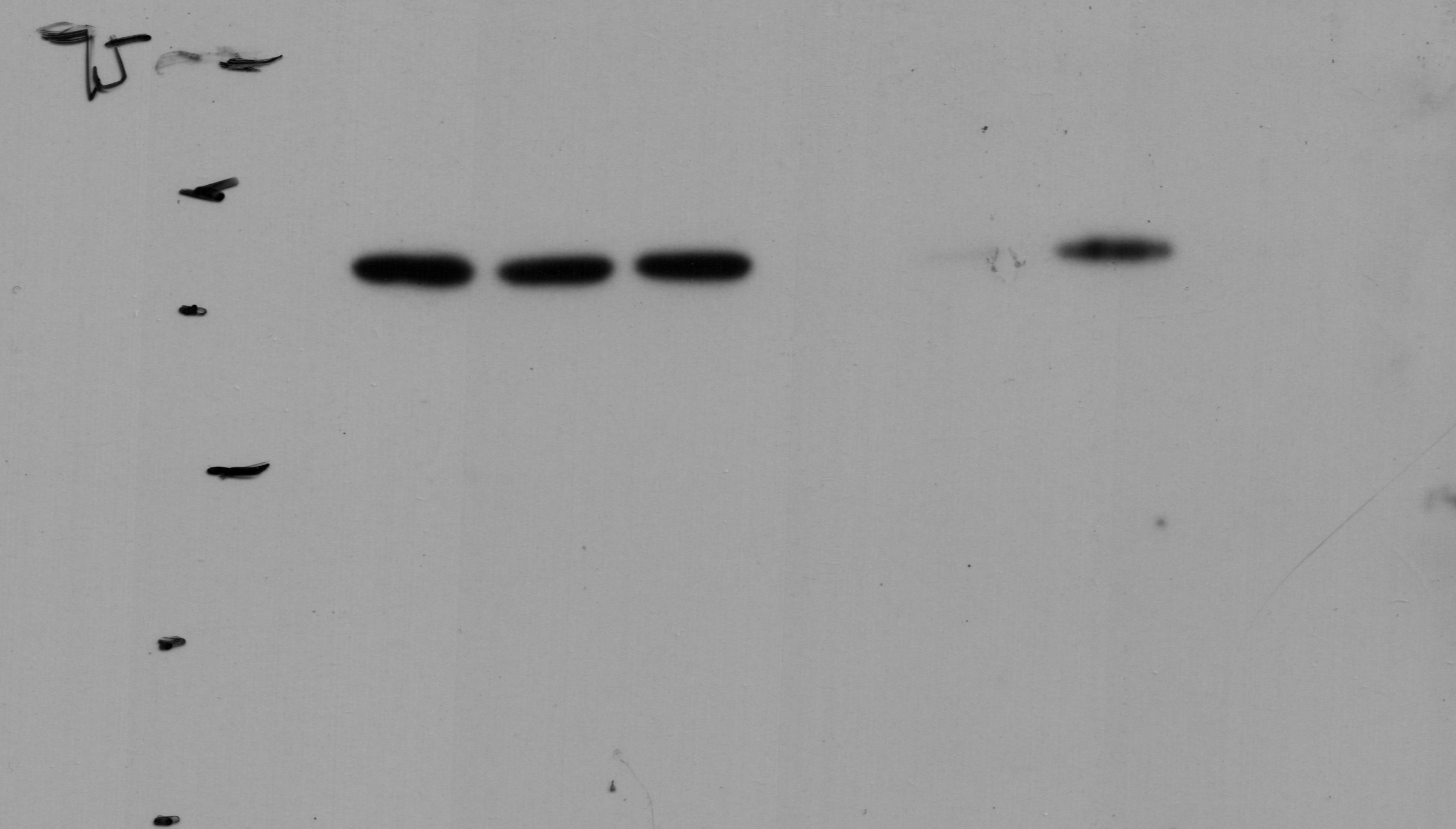

Supplement: Figure 6—source data 2. [file elife-102667-fig6-data2.zip › Figure 6-source data 2/Fig. 6. E1.tif]

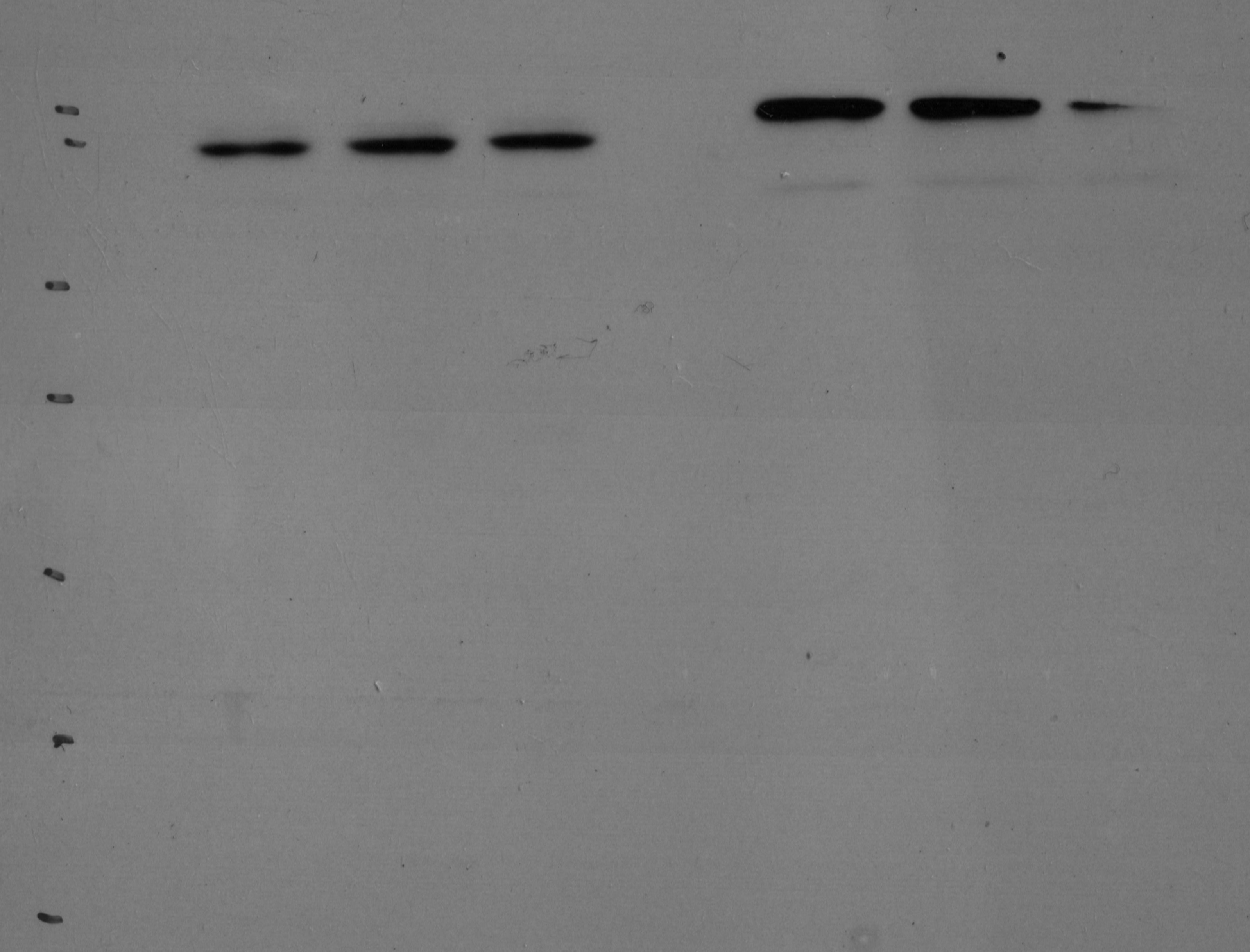

Supplement: Figure 6—source data 2. [file elife-102667-fig6-data2.zip › Figure 6-source data 2/Fig. 6. E2.tif]

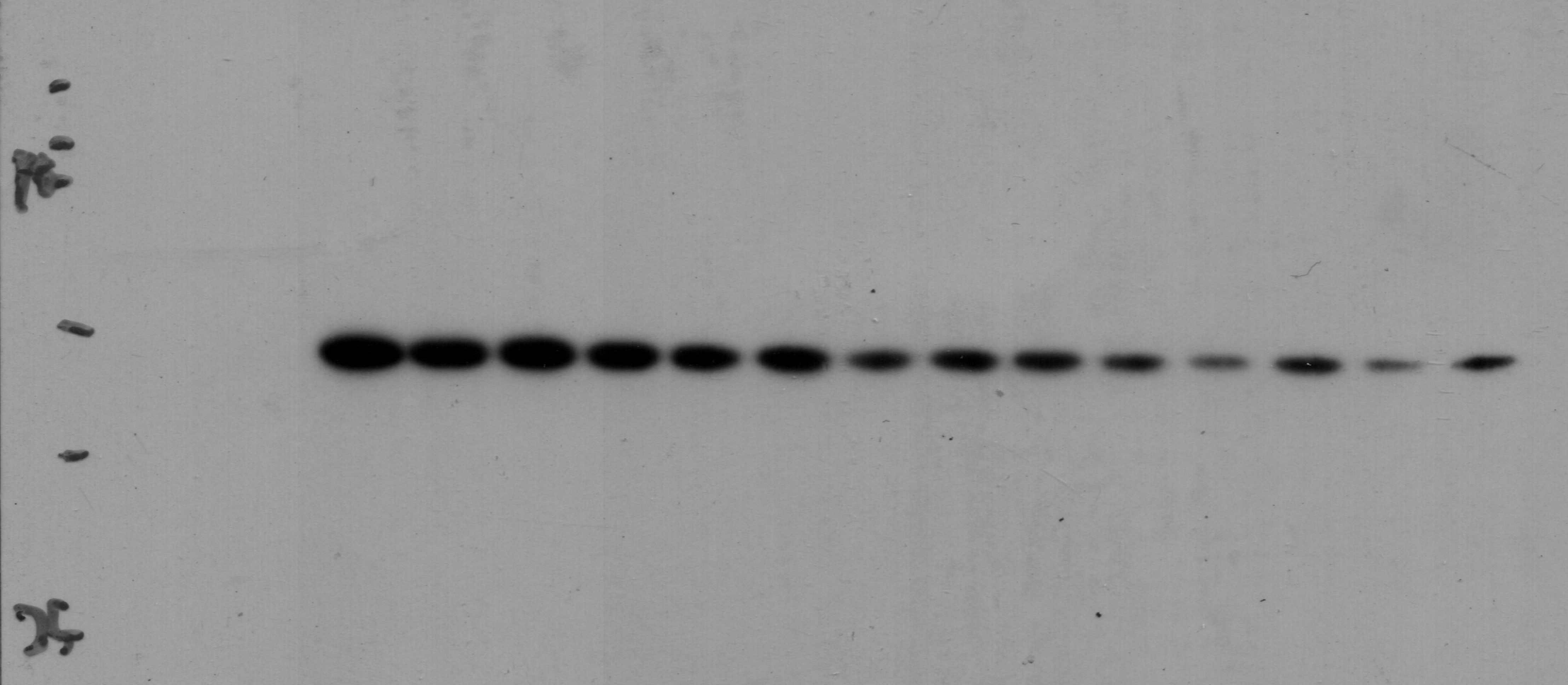

Supplement: Figure 6—source data 2. [file elife-102667-fig6-data2.zip › Figure 6-source data 2/Fig. 6. F1.tif]

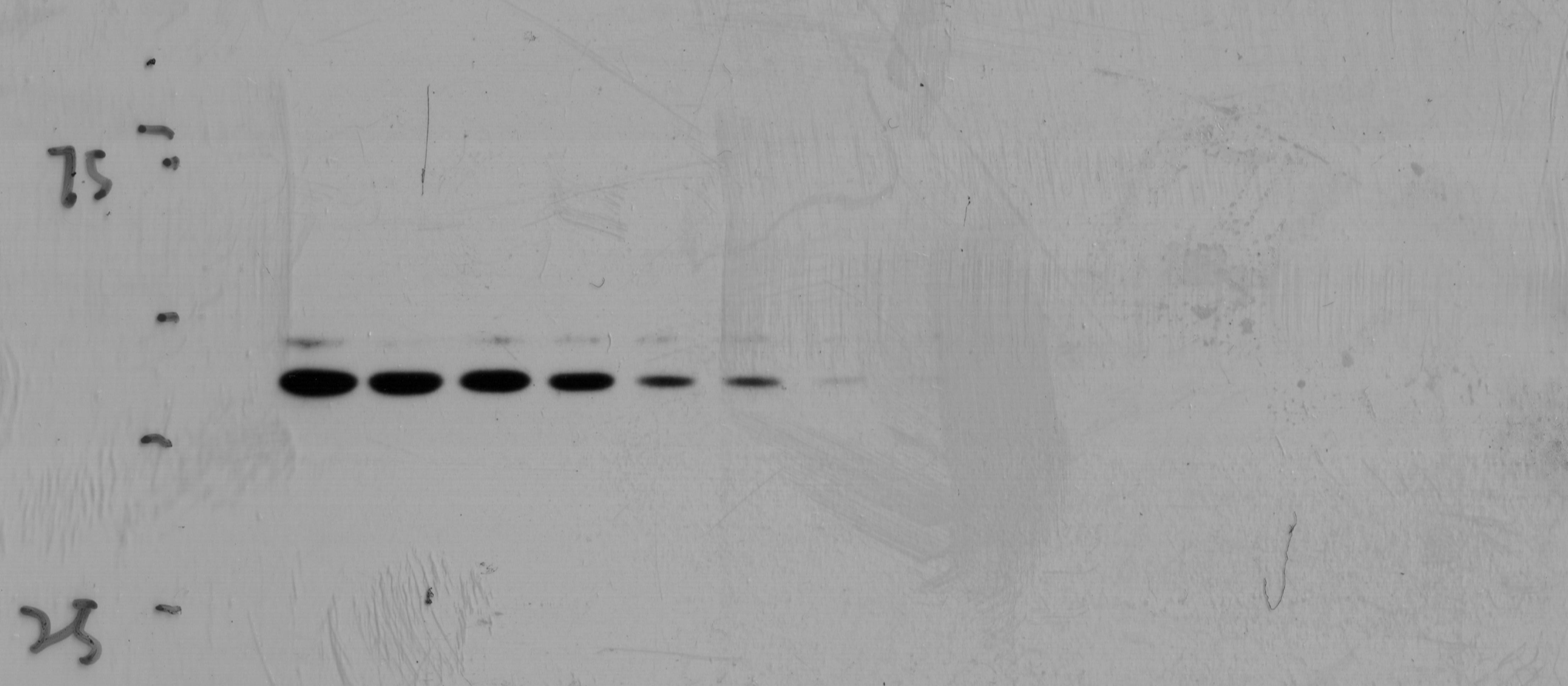

Supplement: Figure 6—source data 2. [file elife-102667-fig6-data2.zip › Figure 6-source data 2/Fig. 6. F2.tif]

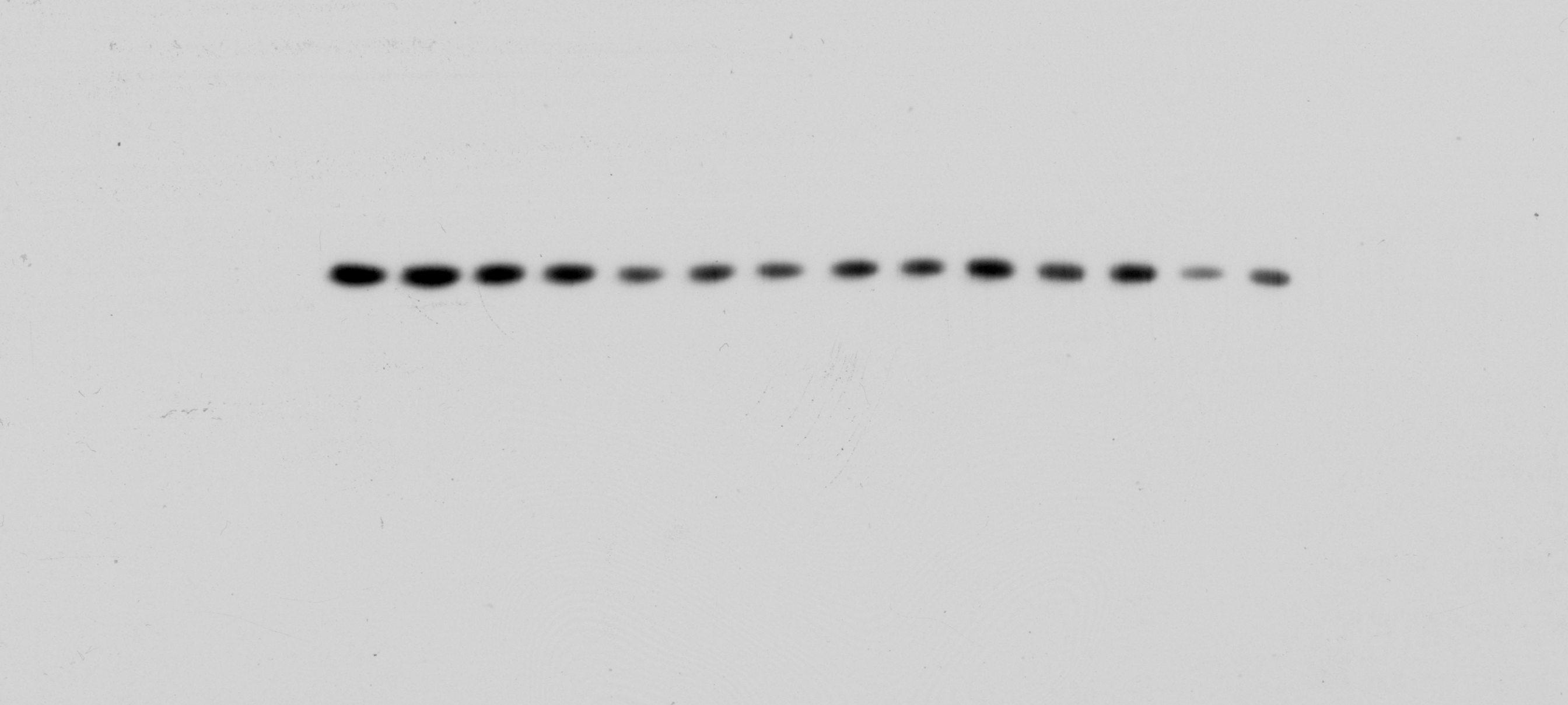

Supplement: Figure 6—source data 2. [file elife-102667-fig6-data2.zip › Figure 6-source data 2/Fig. 6. G1.tif]

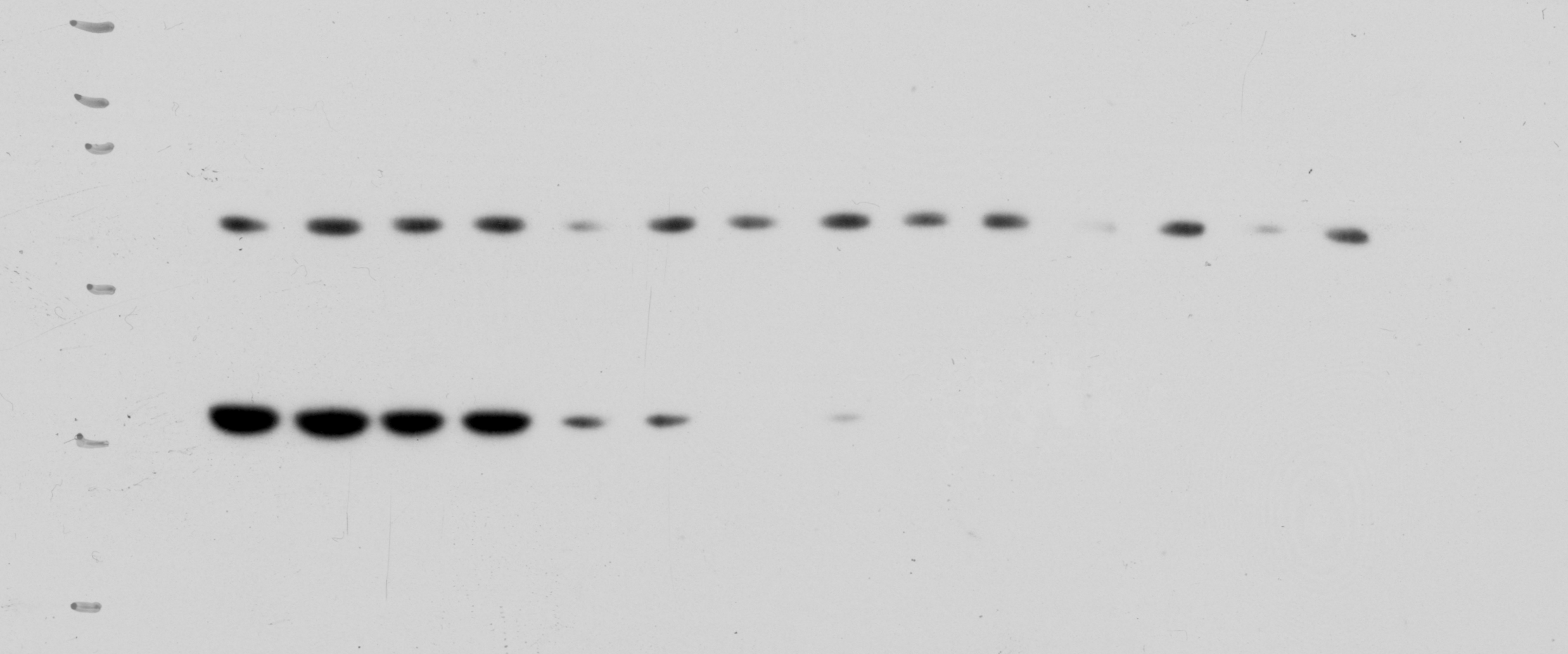

Supplement: Figure 6—source data 2. [file elife-102667-fig6-data2.zip › Figure 6-source data 2/Fig. 6. G2.tif]

**Figure 6—figure supplement 1**

**B**

HEK293T

LC3

Actin

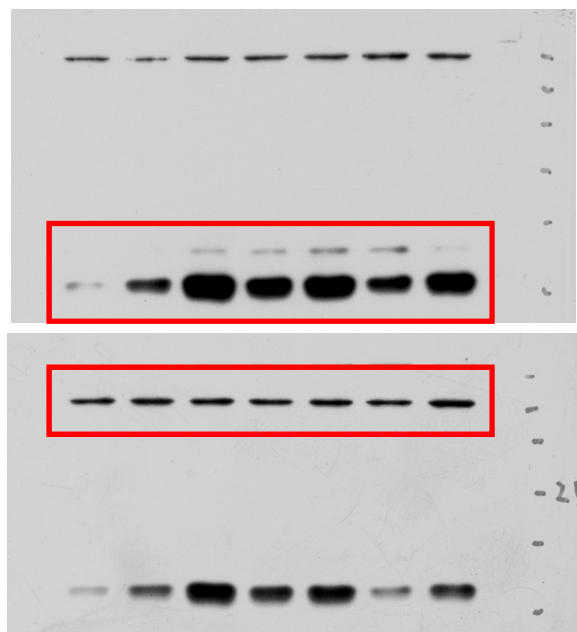

A375

LC3

Actin

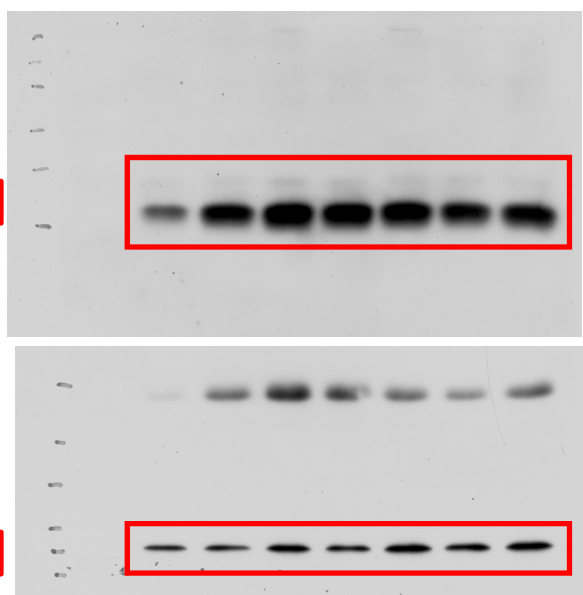

Supplement: Figure 6—figure supplement 1—source data 1. [file elife-102667-fig6-figsupp1-data1.zip › Figure 6—figure supplement 1-source data 1.pdf]

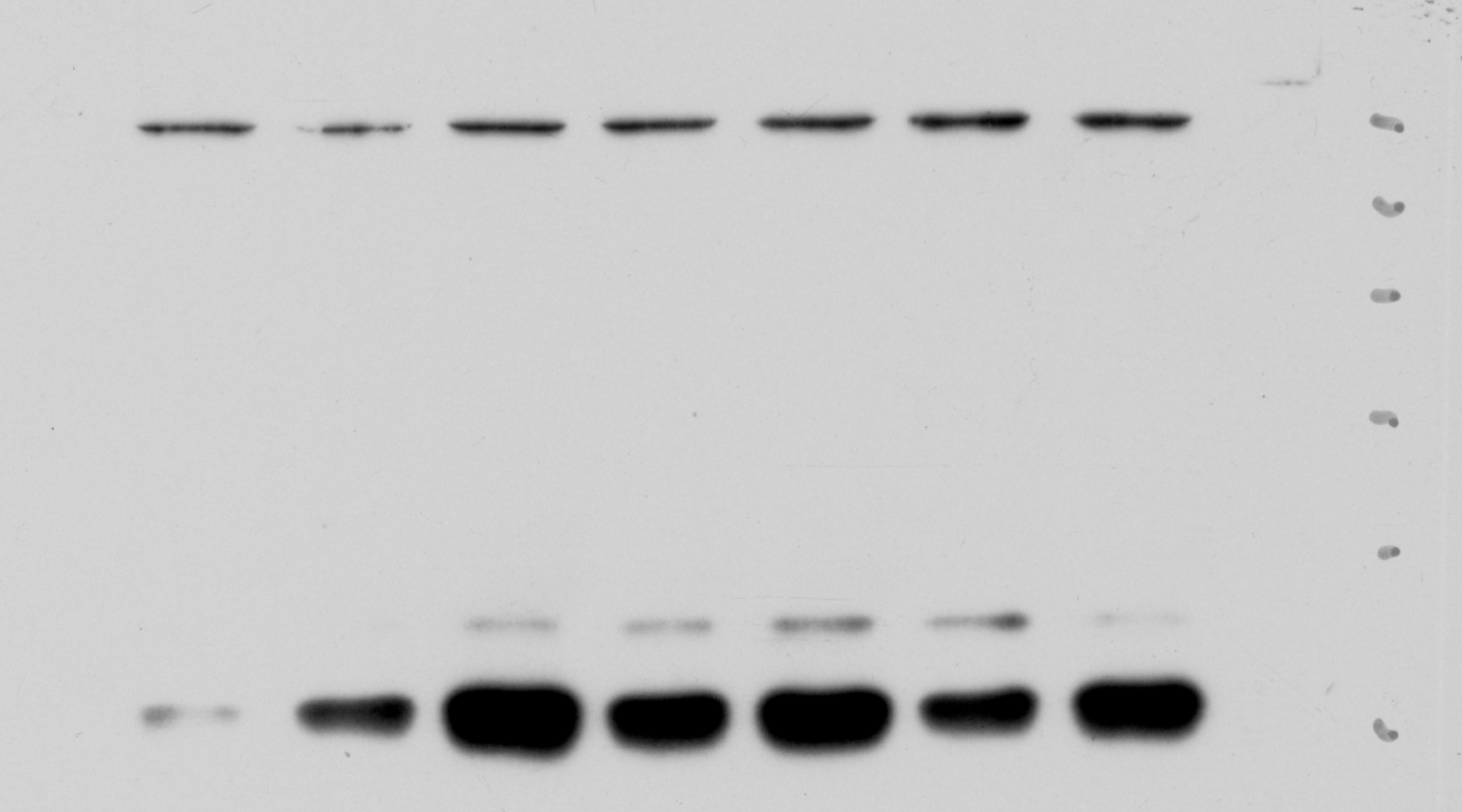

Supplement: Figure 6—figure supplement 1—source data 2. [file elife-102667-fig6-figsupp1-data2.zip › Figure 6—figure supplement 1-source data 2/Figure 6—figure supplement 1. B1.tif]

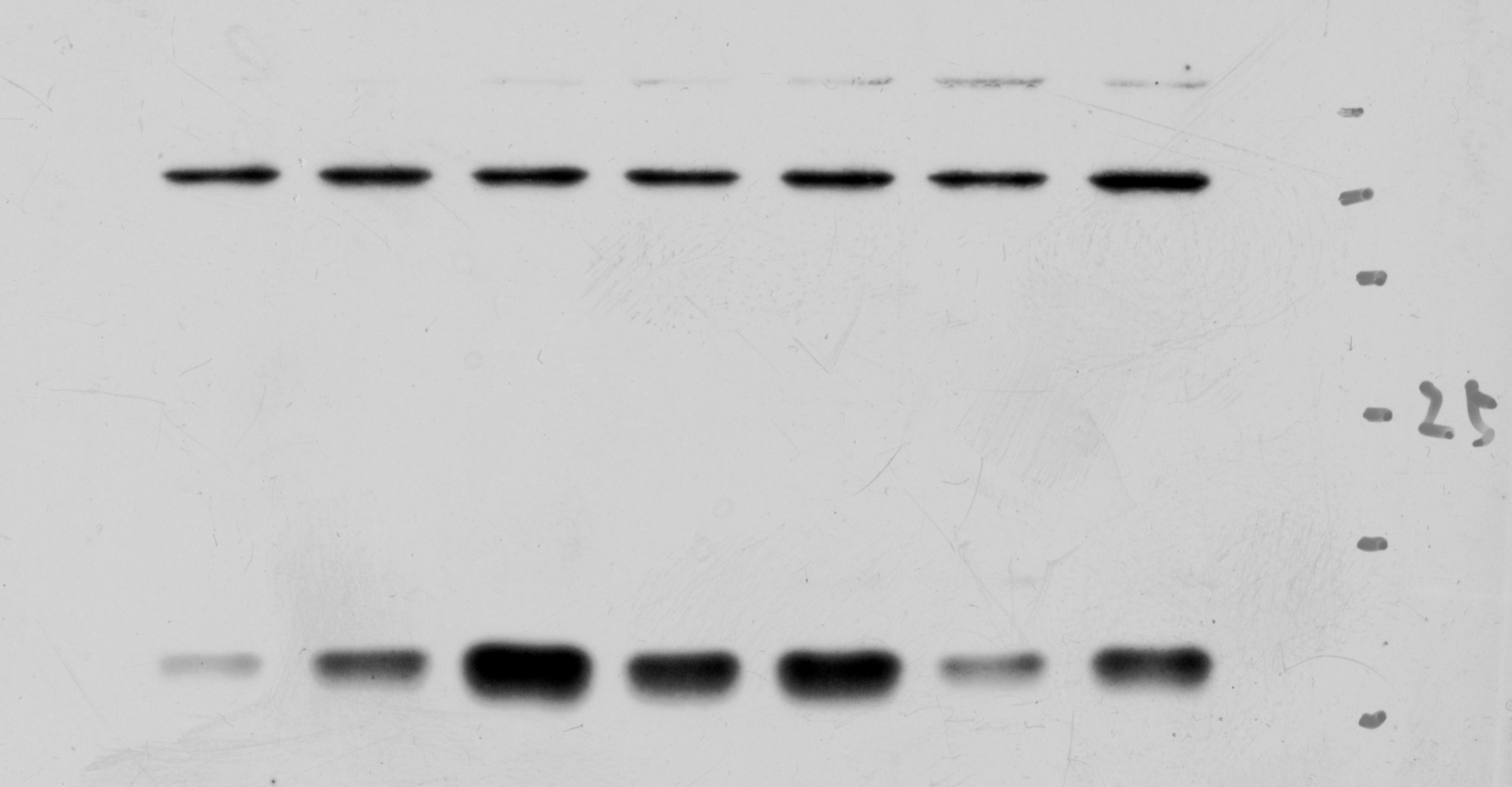

Supplement: Figure 6—figure supplement 1—source data 2. [file elife-102667-fig6-figsupp1-data2.zip › Figure 6—figure supplement 1-source data 2/Figure 6—figure supplement 1. B2.tif]

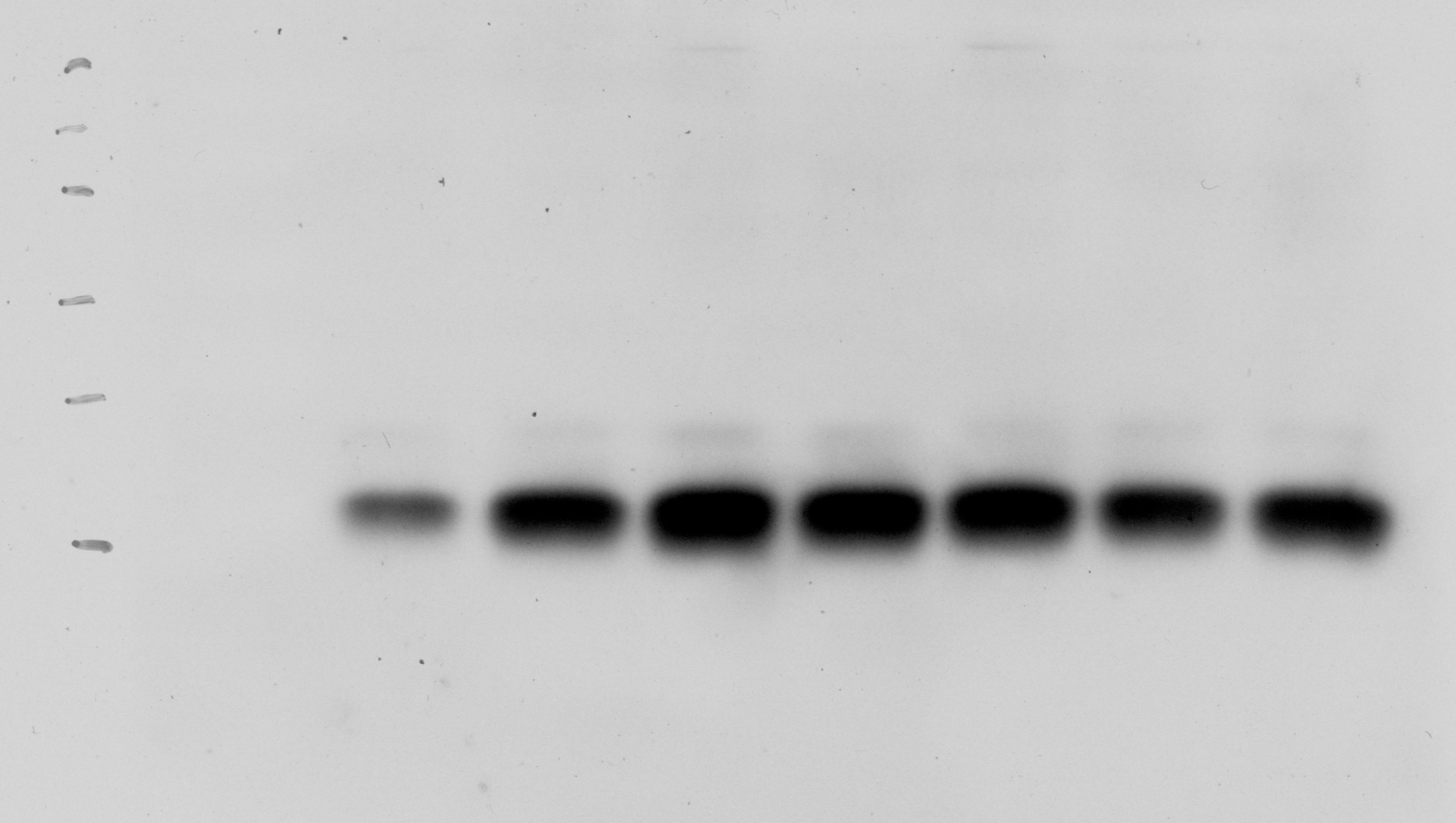

Supplement: Figure 6—figure supplement 1—source data 2. [file elife-102667-fig6-figsupp1-data2.zip › Figure 6—figure supplement 1-source data 2/Figure 6—figure supplement 1. B3.tif]

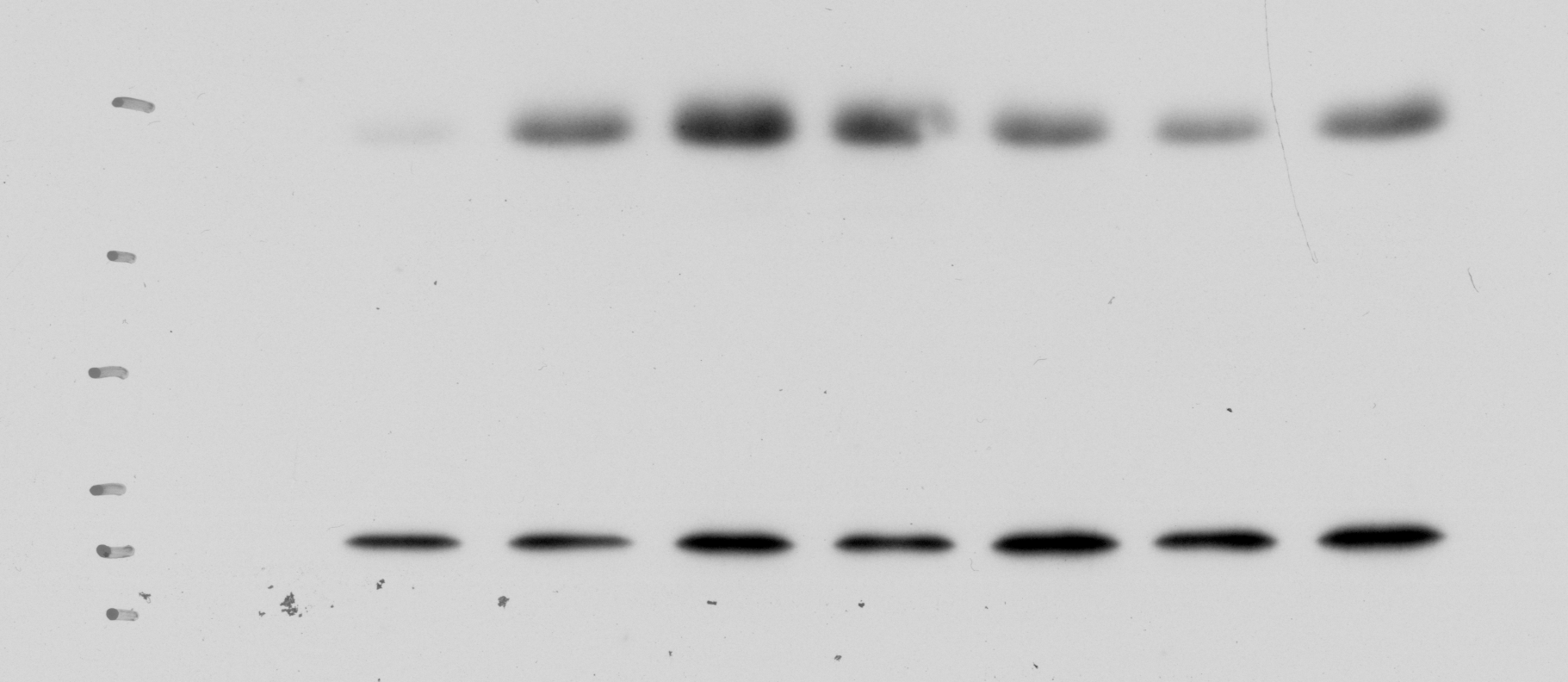

Supplement: Figure 6—figure supplement 1—source data 2. [file elife-102667-fig6-figsupp1-data2.zip › Figure 6—figure supplement 1-source data 2/Figure 6—figure supplement 1. B4.tif]

**Figure 7**

**C**

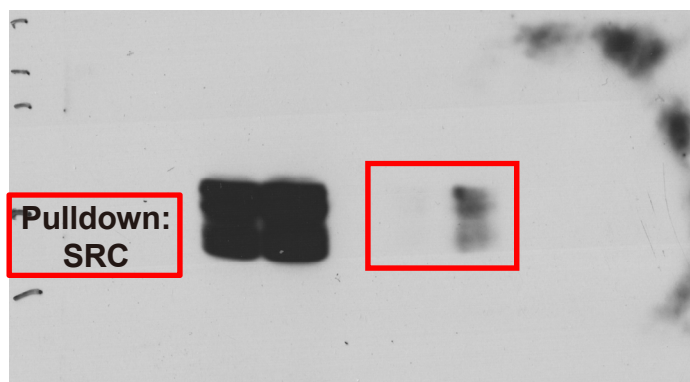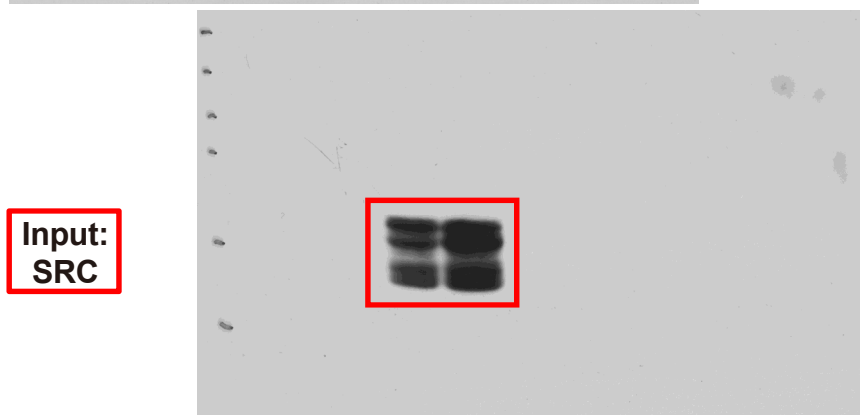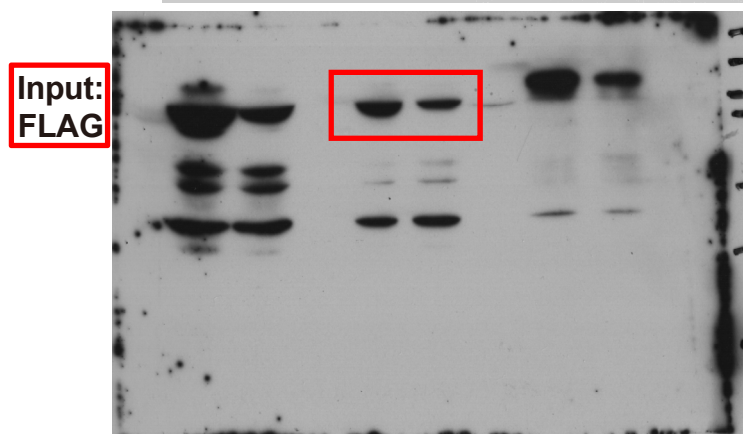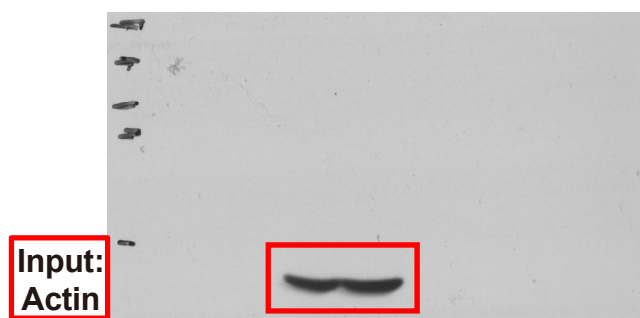

Supplement: Figure 7—source data 1. [file elife-102667-fig7-data1.zip › Figure 7-source data 1.pdf]

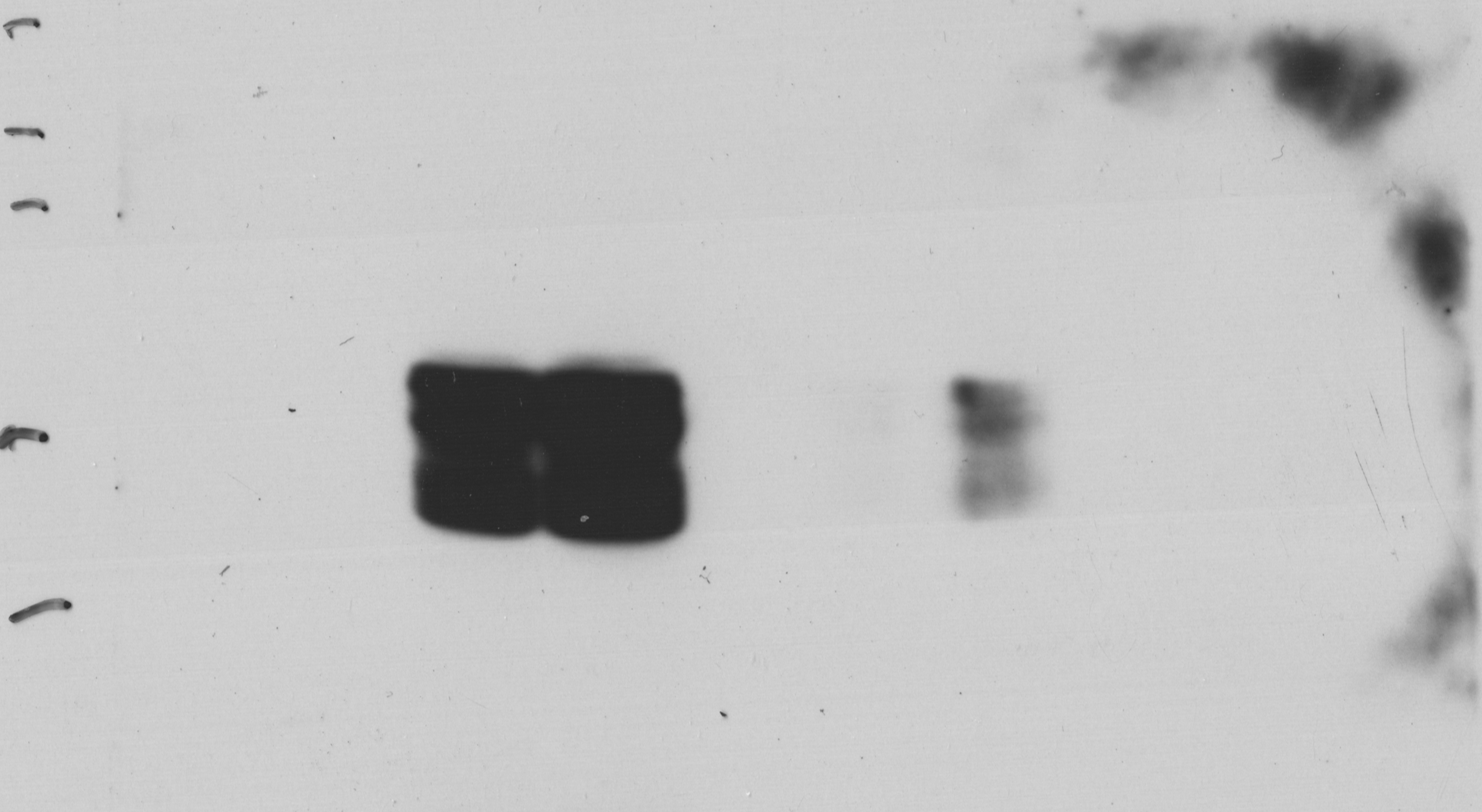

Supplement: Figure 7—source data 2. [file elife-102667-fig7-data2.zip › Figure 7-source data 2/Fig. 7. C1.tif]

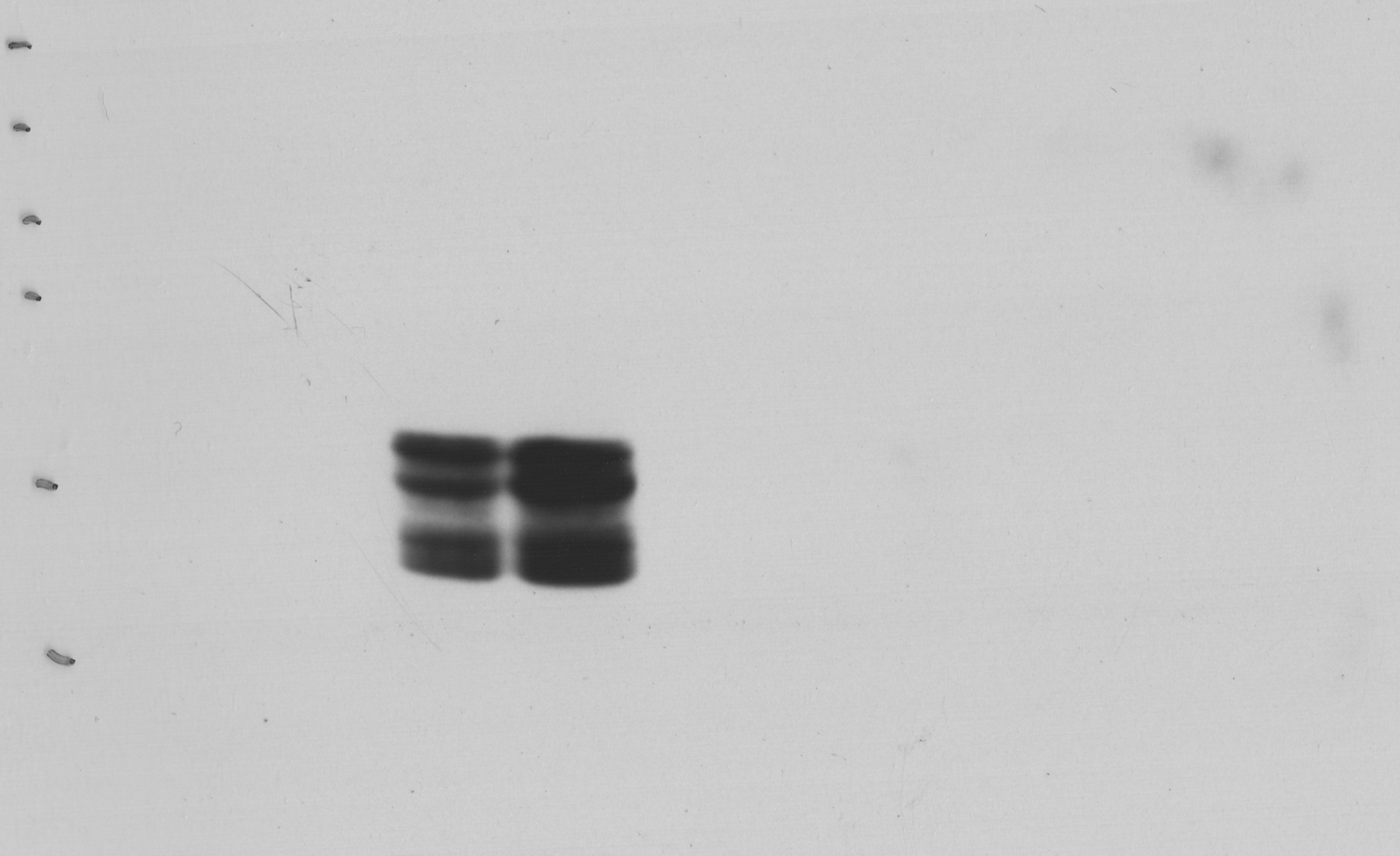

Supplement: Figure 7—source data 2. [file elife-102667-fig7-data2.zip › Figure 7-source data 2/Fig. 7. C2.tif]

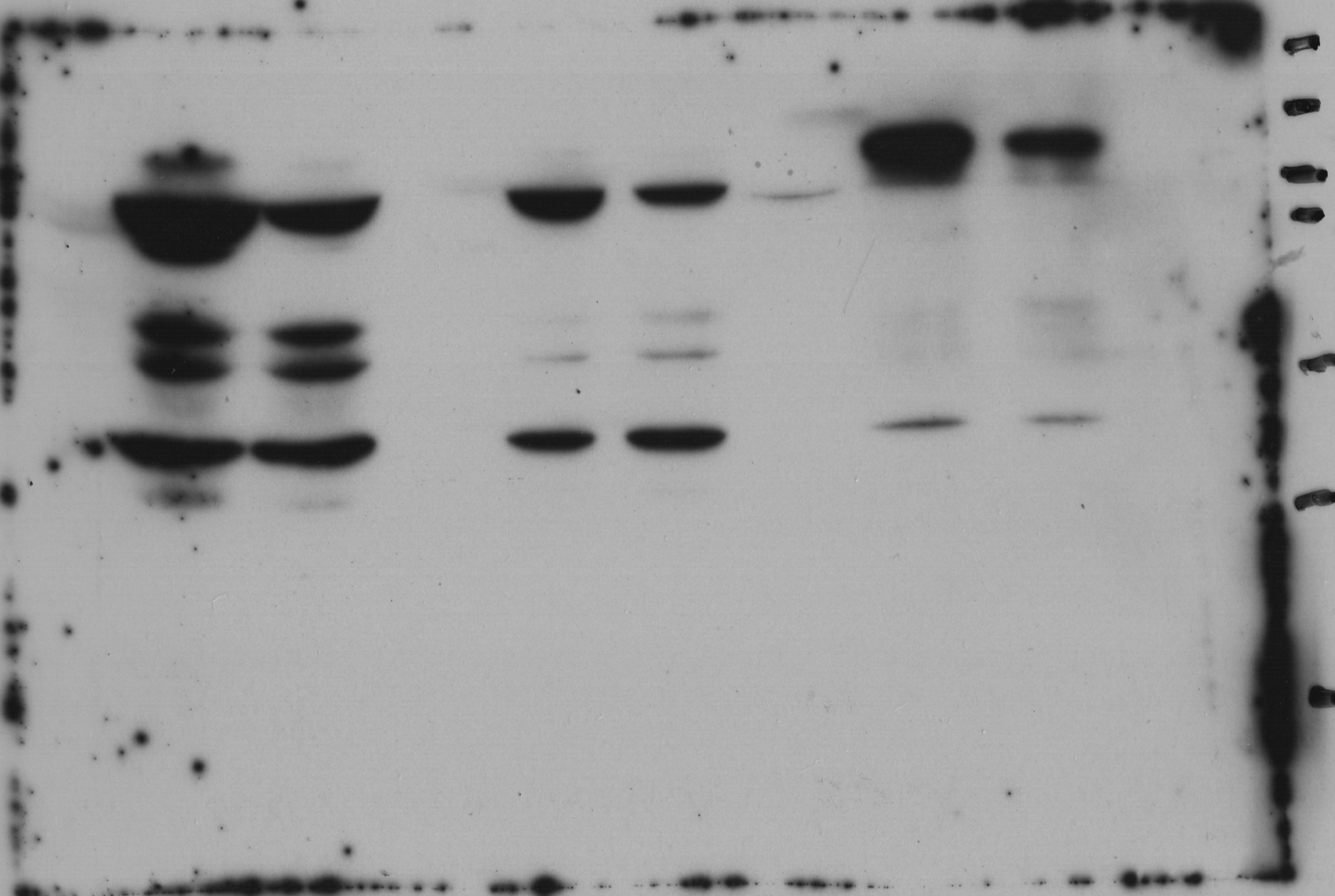

Supplement: Figure 7—source data 2. [file elife-102667-fig7-data2.zip › Figure 7-source data 2/Fig. 7. C3.tif]

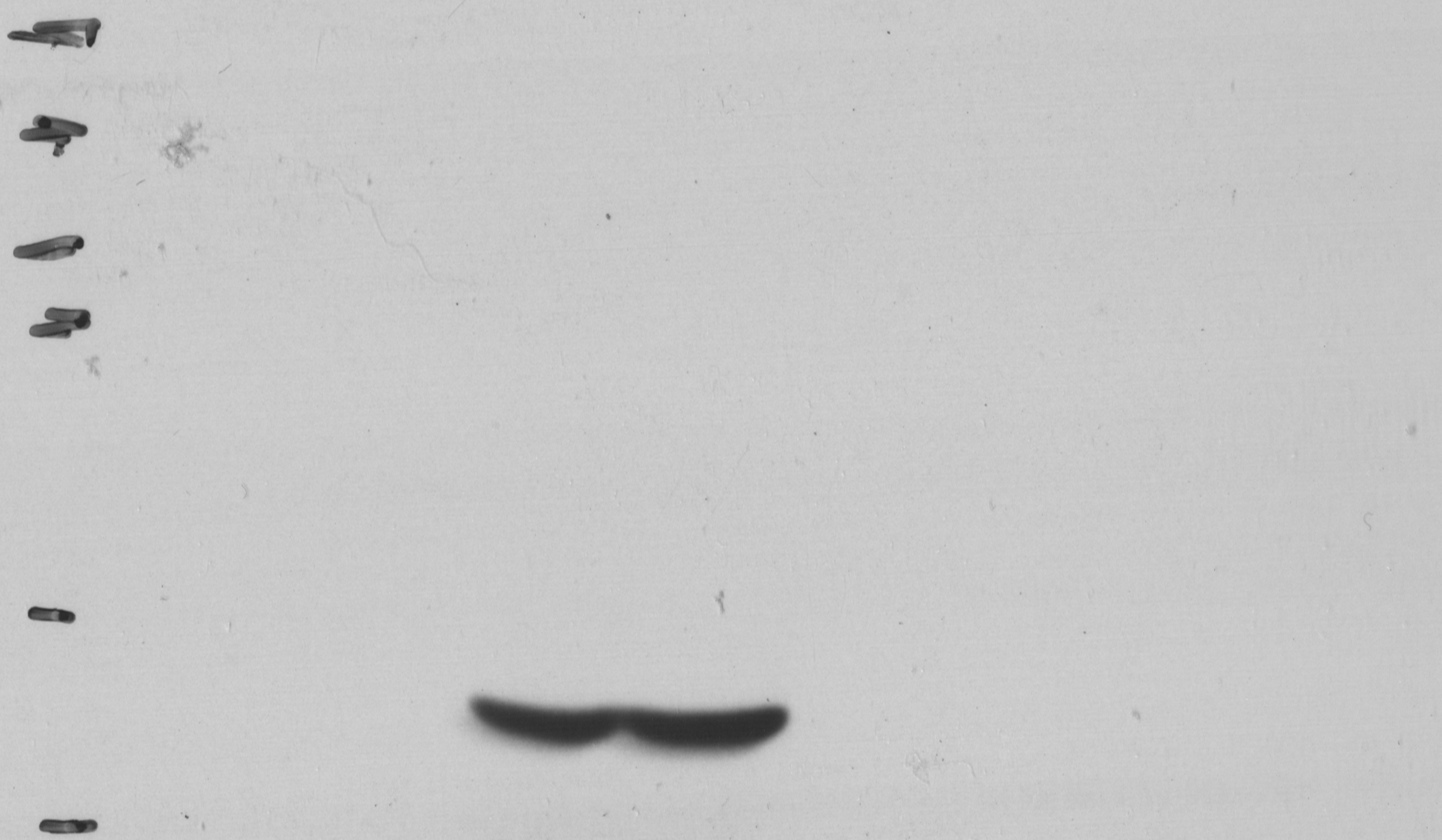

Supplement: Figure 7—source data 2. [file elife-102667-fig7-data2.zip › Figure 7-source data 2/Fig. 7. C4.tif]
